# Construction of Fused Tropone Systems Through Intramolecular Rh(I)-Catalyzed Carbonylative [2+2+2+1] Cycloadditon of Triynes

**DOI:** 10.3389/fchem.2018.00401

**Published:** 2018-09-10

**Authors:** Yu-Han G. Teng, Chih-Wei Chien, Wen-Hua Chiou, Tadashi Honda, Iwao Ojima

**Affiliations:** ^1^Department of Chemistry, Stony Brook University, Stony Brook, NY, United States; ^2^Department of Chemistry, National Chung Hsing University, Taichung, Taiwan

**Keywords:** tropone, [2+2+2+1] cycloaddition, Rh complex catalyst, higher order cycloaddition, carbonylative cycloaddition, triynes

## Abstract

“Tropone” is a non-benzenoid aromatic skeleton that can be found in a variety of natural products. This cyclohepta-2,4,6-trien-1-one skeleton appears simple, but there have been no straightforward ways to construct this molecular architecture. It is conceivable that this molecule can be constructed via a higher order cycloaddition of three acetylene units and CO, but such process was not known until we have discovered that the carbonylative [2+2+2+1] cycloaddition of triynes can take place in the presence of a Rh complex catalyst and CO. However, this highly challenging process is naturally accompanied by ordinary [2+2+2] cyclotrimization products, i.e., benzenes, as side products. A mechanistic study led to two competing processes wherein the critical CO insertion occurs either to a rhodacyclopentadiene intermediate (Path A) or a rhodacycloheptatriene intermediate (Path B). The DFT analysis of those two pathways disclosed that the Path A should be the one that yields the carbonylative [2+2+2+1] cycloaddition products, i.e., fused tricyclic tropones. A further substrate design, inspired by colchicine structure, led to the almost exclusive formation of a fused tetracyclic tropone from a triyne bearing 1,2-disubstituted benzene moiety in a single step and excellent yield.

## Introduction

**Scheme 1:**
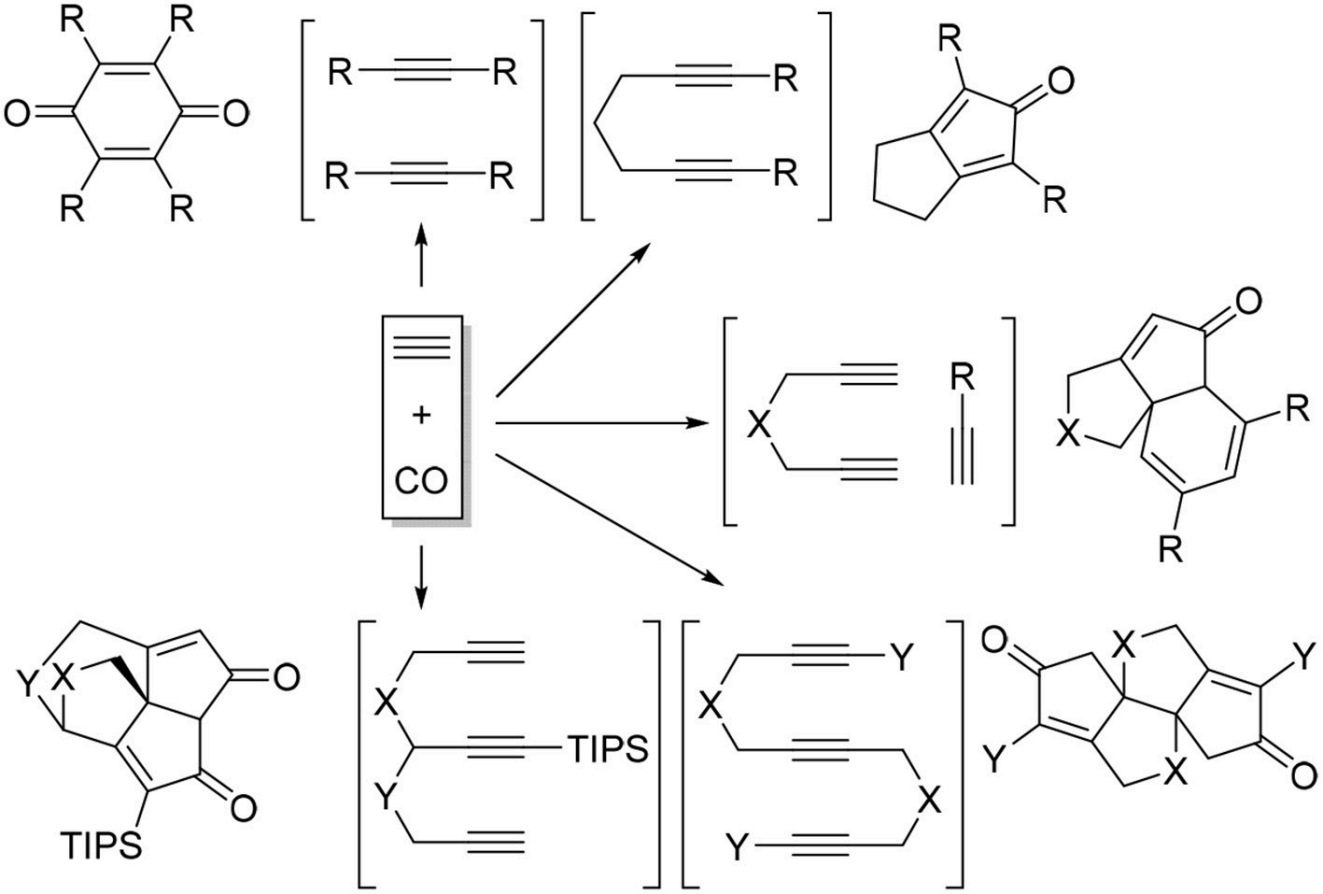
Carbonylative cycloadditions of alkynes.

Transition metal-catalyzed carbocyclization and cycloaddition of unsaturated motifs have proven to be among the most efficient carbon-carbon bond-forming transformations for constructing complex polycyclic systems that are often difficult or impossible to construct by other means (Lautens et al., 1996; Ojima et al., 1996). Among those reactions, cyclotrimerization of alkynes has been the most studied process (Saito and Yamamoto, 2000; Shibata and Tsuchikama, 2008). Inter- and intramolecular alkyne cyclotrimerizations with various transition metal complexes furnished wide varieties of polysubstituted benzene derivatives (Saito and Yamamoto, 2000; Kotha et al., 2005; Chopade and Louie, 2006). When the cycloaddition of alkynes carried out under carbon monoxide atmosphere, a range of interesting carbonylative cycloaddition products were observed instead of benzene formation (Scheme 1) (Gesing et al., 1980; Badrieh et al., 1994; Son et al., 2000a,b, 2001; Shibata et al., 2001, 2002; Sugihara et al., 2001; Huang and Hua, 2007).

**Scheme 2:**
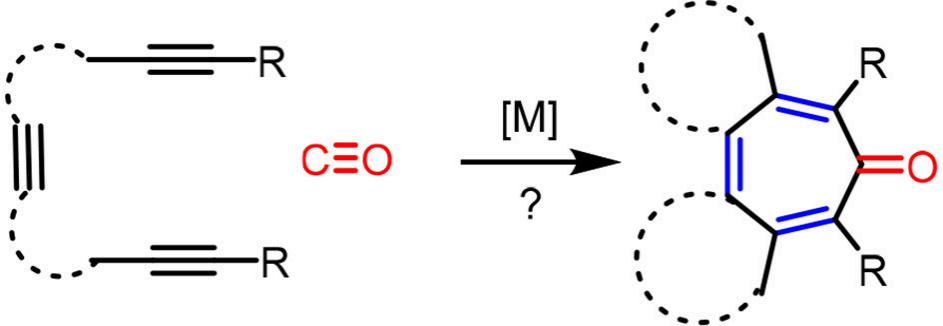
Hypotherical carbonylative [2+2+2+1] cycloaddition route to tropones in one step.

It is clear that cycloaddition of alkynes is not limited to aromatization to benzene derivatives. For examples, challenging compounds such as cyclopentadienone that are anti-aromatic can be prepared by a transition-metal catalyzed cycloaddition of alkynes (Sugihara et al., 2001). We envisioned that other non-benzenoid aromatic skeletons can also be prepared under appropriate conditions. For example, cyclohepta-2,4,6-trien-1-one, generally known as “tropone” (Dewar, 1945), is a non-benzenoid aromatic skeleton that can be found in various biologically active molecules (Erdtman and Gripenberg, 1948; Pauson, 1955; Polonsky et al., 1983; Ginda et al., 1988; Wu et al., 1996; Graening and Schmalz, 2004; Zhao, 2007). Tropone's structure is deceptively simple, but there are no straightforward methods to prepare tropone and its derivatives (Pietra, 1973). Conceptually, the formation of cycloheptatrienone from three alkynes and CO is the most straightforward synthetic route (Scheme 2), but such a process is not known in the literature to date. We report here the discovery of a facile [2+2+2+1] cycloaddition of triynes with CO, catalyzed by Rh-complexes, to form fused tropones in one step.

## Materials and methods

### General experimental procedures

All chemicals were obtained from either Sigma-Aldrich or Acros Organics and used as is, unless otherwise noted. All reactions were performed under Schlenk conditions with oven dried glassware, unless otherwise noted. Dry solvents were degassed under nitrogen and were dried using the PURESOLV system (Inovatative Technologies, Newport, MA). All reactions were monitored by thin layer chromatography (TLC) using E. Merck 60F254 precoated silica gel plates. Flash chromatography was performed with the indicated solvents and using Fisher silica gel (particle size 170–400 Mesh). Yields refer to chromatographically and spectroscopically pure compounds. ^1^H and ^13^C were obtained using either 300 MHz Varian Gemni 2300 (75 MHz ^13^C) spectrometer or the 400 MHz Varian INOVA 400 (100 MHz ^13^C) spectrometer in CDCl_3_ as a solvent. Chemical shifts (δ) are reported in ppm and standardized with solvent as internal standard based on literature reported values (Gottilieb et al., 1997). Melting points were measured with a Thomas Hoover capillary melting point apparatus and are uncorrected.

### Synthesis of triynes

#### 5,5,10,10-tetra(carbethoxy)tetradeca-2,7,12-triyne (1b)


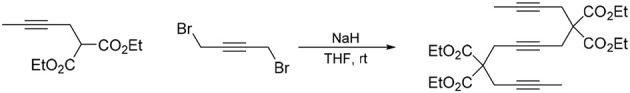


To a suspension of NaH (0.30 g, 7.5 mmol) in THF (20 mL) was added a solution of diethyl 2-(but-2-ynyl)malonate (Bennacer et al., 2005) (1.5 g, 7.1 mmol) in THF (10 mL). The reaction mixture was stirred under N_2_ atmosphere at room temperature for 1 h. To the reaction mixture was added a solution of 1,4-dibromobut-2-yne (0.76 g, 3.6 mmol) in THF (20 mL) dropwise with stirring at room temperature and the reaction mixture was stirred at room temperature for 16 h. The reaction was quenched with addition of water, followed by extraction with Et_2_O. The combined organic layers were washed with brine and dried over MgSO_4_, and concentrated under reduced pressure. The crude product was purified by flash column chromatography (silica gel, EtOAc/hexanes = 0% 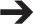 10%) to afford **1b** as a light yellow oil (1.37 g, 81%): ^1^H NMR (300 MHz, CDCl_3_): δ 1.24 (t, 12 H, *J* = 7.2 Hz), 1.74 (t, 6 H, *J* = 2.4 Hz), 2.85 (q, 4 H, *J* = 2.4 Hz), 2.90 (s, 4 H), 4.20 (q, 4 H, *J* = 7.2 Hz); ^13^C NMR (100 MHz, CDCl_3_): δ 3.36, 14.21, 22.99, 56.99, 61.94, 73.48, 77.93, 78.97, 169.27; HRMS (ES) m/z calcd for C_26_H_35_O_8_ (M + H)^+^: 475.2332, found 475.2341 (Δ 1.9 ppm).

#### 5,10-dioxatetradeca-2,7,12-triyne (1c)


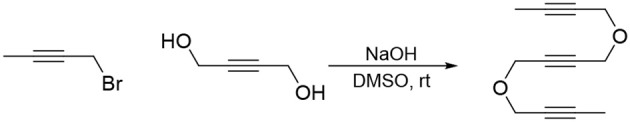


To a solution of NaOH (2.79 g, 69.7 mmol) in DMSO (20 mL) was added a solution of but-2-yne-1,4-diol (2.00 g, 23.2 mmol) in DMSO (10 mL). The reaction mixture was stirred under N_2_ atmosphere at room temperature for 1 h. To the reaction mixture was added a solution of 1-bromobut-2-yne (7.67 g, 58.1 mmol) in DMSO (10 mL) dropwise with stirring at room temperature and the reaction mixture was stirred at room temperature for 16 h. The reaction was quenched with addition of water, followed by extraction with Et_2_O. The combined organic layers were washed with brine and dried over MgSO_4_, and concentrated under reduced pressure. The crude product was purified by flash column chromatography (silica gel, EtOAc/hexanes = 0% 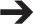 10%) to afford **1c** as a light yellow oil (3.18 g, 72%): ^1^H NMR (300 MHz, CDCl_3_): δ1.84 (t, 6 H, *J* = 2.4 Hz), 4.20 (q, 4 H, *J* = 2.4 Hz), 4.27 (s, 4 H). ^13^C NMR (100 MHz, CDCl_3_): δ 3.41, 56.52, 57.22, 74.21, 82.18, 83.14. All data are in agreement with those reported in the literature (Yamamoto et al., 2003; Geny et al., 2009).

#### 5-(4-methylbenzenesulfonyl)-10,10-di(carbethoxy)-5-azatetradeca-2,7,12-triyne (1d)


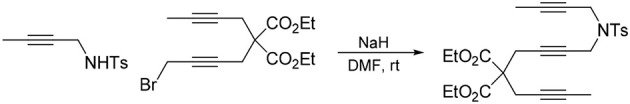


To a suspension of NaH (0.14 g, 3.4 mmol) in DMF (10 mL) was added a solution of *N*-(but-2-ynyl)-*N*-(4-methylbenzenesulfonyl)amine (Bennacer et al., 2005) (0.61 g, 2.7 mmol) in DMF (10 mL) dropwise with stirring at room temperature. The reaction mixture was stirred under nitrogen atmosphere at room temperature for 1 h. To the reaction mixture was added a solution of 1-bromo-5,5-di(carbethoxy)-nona-2,7-diyne (Bennacer et al., 2004, 2005) (0.93 g, 2.7 mmol) in DMF (5 mL) dropwise with stirring at room temperature and the reaction mixture was stirred at room temperature for 16 h. The reaction was quenched with addition of water, followed by extraction with Et_2_O. The combined organic layers were washed with brine, dried over MgSO_4_, and concentrated under reduced pressure. The crude product was purified by flash column chromatography (silica gel, EtOAc/hexanes = 0% 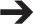 20%) to afford **1d** as a light yellow oil (1.02 g, 77%): ^1^H NMR (300 MHz, CDCl_3_): δ 1.24 (t, 6 H, *J* = 7.2 Hz), 1.63 (t, 3 H, *J* = 2.4 Hz), 1.75 (t, 3 H, *J* = 2.4 Hz), 2.43 (s, 3 H), 2.72 (q, 2 H, *J* = 2.4 Hz), 2.82 (t, 2 H, *J* = 2.1 Hz), 4.04 (q, 2 H, *J* = 2.4 Hz), 4.07 (t, 2 H, *J* = 2.1 Hz), 4.18 (q, 4 H, *J* = 7.2 Hz); 7.30 (d, 2 H, *J* = 8.1 Hz), 7.69 (d, 2 H, *J* = 8.1 Hz); ^13^C NMR (100 MHz, CDCl_3_): δ 3.3, 3.5, 14.0, 21.5, 22.8, 22.9, 36.5, 56.6, 61.8, 71.5, 73.0, 75.8, 79.0, 80.5, 81.7, 127.9, 129.4, 135.5, 143.5, 168.9; HRMS (ES) m/z calcd for C_26_H_31_NO_6_S (M + H)^+^: 486.1950, found 486.1937 (Δ 2.7 ppm).

#### 5,5,10,10-tetra(carbethoxy)pentadeca-2,7,13-triyne (1e)


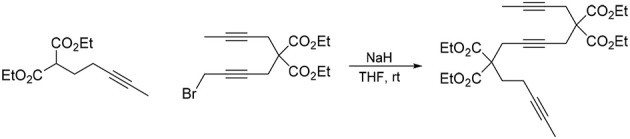


A solution of diethyl 2-(pent-3-ynyl)malonate (Ansell et al., 1968) (0.60 g, 2.65 mmol) in THF (5 mL) was added to a suspension of NaH (60% dispersion in mineral oil, 0.12 g, 3.0 mmol) in THF (20 mL) under nitrogen and the resulting mixture was stirred at room temperature for 30 min. A solution of 1-bromo-5,5-di(carbethoxy)-nona-2,7-diyne (Bennacer et al., 2004, 2005) (0.91 g, 2.65 mmol) in THF (5 mL) was then added dropwise and the reaction mixture was stirred at room temperature overnight. Water was added followed by extraction with Et_2_O. The combined organic layers were washed with brine, dried over MgSO_4_ and concentrated under reduced pressure. The crude was purified by flash chromatography (silica gel, EtOAc/hexanes = 5% 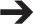
10%) to give **1e** as a colorless oil (1.15 g, 89%): MR (300 MHz, CDCl_3_): δ 1.24 (t, 12 H, *J* = 7.2 Hz), 1.74 (m, 6 H), 2.03–2.22 (m, 4 H), 2.77–2.89 (m, 6 H), 4.20 (m, 8 H, *J* = 7.2 Hz); ^13^C NMR (100 MHz, CDCl_3_): δ3.48, 13.99, 14.02, 14.06, 22.77, 22.80, 22.95, 31.24, 56.39, 56.75, 61.58, 61.75, 73.21, 76.12, 77.23, 77.57, 77.79, 78.81, 169.07, 169.95; HRMS (ES) m/z calcd for C_27_H_40_NO_8_ (M + NH_4_)^+^: 506.2748, found 506.2755 (Δ 1.3 ppm).

#### 1-phenyl-4,4,9,9-tetra(carbethoxy)tetradeca-1,6,12-triyne (1f)


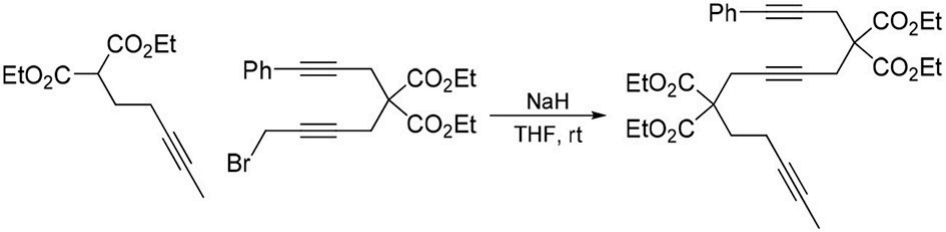


To a suspension of NaH (0.44 g, 60 wt% in mineral oil, 10.9 mmol) in THF (60 mL) was added 1,4-dibromobut-2-yne (7.7 g, 37 mmol) dropwise at 0°C. A solution of diethyl 3-phenylpropargylmalonate (Hicks et al., 1999) (2.5 g, 9.1 mmol) in THF (10 mL) was added to the reaction mixture dropwise. The reaction mixture was stirred at room temperature overnight. The reaction was quenched with addition of water, followed by extraction with Et_2_O. The combined organic layers were washed with water and brine, dried over MgSO_4_, and concentrated under reduced pressure. The excess dibromobutyne was recovered by distillation (100°C /4 mmHg). The crude was purified by flash chromatography (silica gel, EtOAc/hexanes = 5% 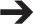 10%) to afford 1-bromo-5,5-di(carbethoxy)-8-phenylocta-2,7-diyne as a light yellow oil (3.10 g, 84%): ^1^H NMR (300 MHz, CDCl_3_): δ 1.28 (t, 6 H, *J* = 7.2 Hz), 3.10(t, 2 H, *J* = 2.4 Hz), 3.17 (s, 2 H), 3.88 (t, 2 H, *J* = 2.4 Hz), 4.25 (q, 4 H, *J* = 7.2 Hz); ^13^C NMR (100 MHz, CDCl_3_): δ14.08, 14.64, 23.24, 23.65, 56.81, 62.06, 78.45, 82.09, 83.76, 83.85, 123.04, 128.05 128.19, 131.65, 168.71.

A solution of diethyl 2-(pent-3-ynyl)malonate (Ansell et al., 1968) (0.56 g, 2.47 mmol) in THF (5 mL) was added to a suspension of NaH (60% dispersion in mineral oil, 0.12 g, 3.0 mmol) in THF (20 mL) under nitrogen and the resulting mixture was stirred at room temperature for 30 min. A solution of 1-bromo-5,5-di(carbethoxy)-8-phenylocta-2,7-diyne (1.0 g, 2.5 mmol) in THF (5 mL) was then added dropwise and the reaction mixture was stirred at room temperature overnight. Water was added followed by extraction with Et_2_O. The combined organic layers were washed with brine, dried over MgSO_4_ and concentrated under reduced pressure. The crude was purified by flash chromatography (silica gel, EtOAc/hexanes = 5% 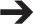
10%) to give **1f** as a colorless oil (1.20 g, 88%): ^1^H NMR (300 MHz, CDCl_3_) δ 1.26 (m, 12 H), 1.74 (t, 3 H, *J* = 2.4 Hz), 2.11 (m, 2 H), 2.25 (t, 2 H, *J* = 2.4 Hz), 2.80 (t, 2 H, *J* = 2.4 Hz), 2.99 (t, 2 H, *J* = 2.4 Hz), 3.15 (s, 2 H), 4.21 (m, 8 H), 7.25–7.38 (m, 5 H)); ^13^C NMR (100 MHz, CDCl_3_): δ 3.44, 13.98, 14.04, 22.96, 23.41, 31.27, 56.39, 56.81, 61.56, 61.87, 76.13, 77.64, 77.73, 77.84, 83.46, 84.20, 123.24, 127.89, 128.14, 131.65, 168.84, 169.91; HRMS (ES) m/z calcd for C_32_H_39_O_8_ (M + H)^+^: 551.2645, found 551.2648 (Δ 0.5 ppm).

#### 1-phenyl-9-(4-methylbenzenesulfonyl)-4,4-di(carbethoxy)-9-azatetradeca-1,6,12-triyne (1g)


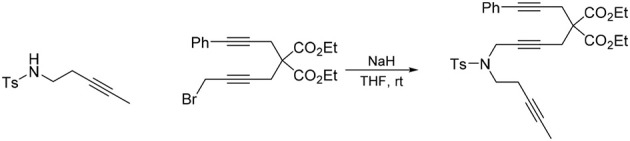


To a suspension of K_2_CO_3_ (0.874 g, 6.3 mmol) in MeCN (5 mL) were added a solution of *N*-(pent-3-ynyl)-*N*-(4-methylbenzenesulfonyl)amine (Yin et al., 2007) (0.50 g, 2.11 mmol) in MeCN (5 mL) and a solution of 1-bromo-5,5-di(carbethoxy)-8-phenylocta-2,7-diyne (0.853 g, 2.11 mmol) in MeCN (5 mL). The reaction mixture was heated at 90°C for overnight. The reaction mixture was diluted with DCM and filtered through Celite®, and the solution was concentrated under reduced pressure. The crude was purified by flash chromatography (silica gel, EtOAc/hexanes = 0% 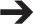 15%) to give **1g** as a light yellow oil (0.876 g, 74%): ^1^NMR (300 MHz, CDCl_3_) δ 1.24 (t, 6 H, *J* = 7.2 Hz), 1.74 (t, 3 H, *J* = 1.8 Hz), 2.36–2.42 (m, 5 H), 2.84 (t, 2 H, *J* = 1.8 Hz), 2.92 (s, 2 H), 3.28 (t, 2 H, *J* = 1.8 Hz), 4.17–4.22 (m, 6 H), 7.27–7.37 (m, 7 H), 7.70 (m, 2 H); ^13^C NMR (100 MHz, CDCl_3_): δ 3.47, 14.08, 19.08, 21.51, 22.87, 23.44, 37.36, 45.62, 56.54, 62.01, 75.52, 76.33, 77.60, 80.35, 83.69, 83.86, 123.05, 127.53, 128.15, 128.29, 129.59, 131.63, 136.12, 143.52, 168.69; HRMS (ES) m/z calcd for C_32_H_39_N_2_O_6_S (M + NH_4_)^+^: 579.2523, found 579.2530 (Δ 1.1 ppm).

#### 7-(4-methylbenzenesulfonyl)-12,12-di(carbethoxy)-7-azahexadeca-2,9,14-triyne (1h)


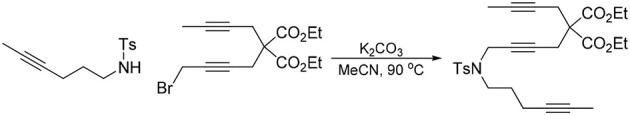


To a suspension of K_2_CO_3_ (0.83 g, 6.0 mmol) in MeCN (15 mL) were added a solution of *N*-(hex-4-ynyl)-*N*-(4-methylbenzenesulfonyl)amine (Luo and Wang, 1992) (0.50 g, 2.0 mmol) in MeCN (5 mL) and a solution of 1-bromo-5,5-di(carbethoxy)-nona-2,7-diyne (0.68 g, 2.0 mmol) in MeCN (5 mL). The reaction mixture was heated at 90°C overnight. The reaction mixture was diluted with DCM, filtered through Celite®, and the filtrate was concentrated under reduced pressure. The crude was purified by flash chromatography (silica gel, EtOAc/hexanes = 0% 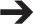 10%) to give **1h** as a light yellow oil (0.91 g, 89%): ^1^NMR (300 MHz, CDCl_3_) δ 1.22 (t, 6 H, *J* = 7.2 Hz), 1.66–1.78 (m, 8 H), 2.17 (m, 2 H), 2.44 (s, 3 H), 2.62 (q, 2 H, *J* = 2.4 Hz), 2.73 (t, 2 H, *J* = 2.1 Hz), 3.20 (t, 2 H, *J* = 7.2 Hz), 4.09 (t, 2 H, *J* = 2.1 Hz), 4.14 (m, 4 H), 7.30 (d, 2 H, *J* = 7.8 Hz), 7.78 (d, 2 H, *J* = 7.8 Hz); ^13^C NMR (100 MHz, CDCl_3_): δ 3.63, 14.17, 14.22, 16.24, 21.76, 22.86, 23.03, 27.44, 37.09, 45.72, 56.67, 61.97, 73.20, 76.17, 76.45, 78.07, 79.10, 80.59, 127.84, 129.71, 136.25, 143.55, 169.03; HRMS (ES) m/z calcd for C_28_H_36_NO_6_S (M + H)^+^: 514.2263, found 514.2257 (Δ 1.2 ppm).

#### 1-phenyl-9-(4-methylbenzenesulfonyl)-4,4-di(carbethoxy)-9-azapentadeca-1,6,13-triyne (1i)


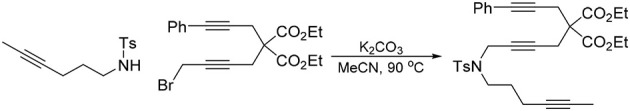


To a suspension of K_2_CO_3_ (0.66 g, 4.8 mmol) in MeCN (5 mL) were added a solution of *N*-(hex-4-ynyl)-*N*-(4-methylbenzenesulfonyl)amine (Luo and Wang, 1992) (0.40 g, 1.59 mmol) in MeCN (5 mL) and a solution of 1-bromo-5,5-di(carbethoxy)-8-phenylocta-2,7-diyne (0.65 g, 1.6 mmol) in MeCN (5 mL). The reaction mixture was heated at 90°C for overnight. The reaction mixture was diluted with DCM and filtered through Celite®, and the solution was concentrated under reduced pressure. The crude was purified by flash chromatography (silica gel, EtOAc/hexanes = 0% 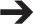 15%) to give **1i** as a light yellow oil (0.653 g, 71%): ^1^NMR (300 MHz, CDCl_3_) δ 1.22 (t, 6 H, *J* = 7.2 Hz), 1.70–1.78 (m, 5 H), 2.17 (m, 2 H), 2.35 (s, 3 H), 2.80 (t, 2 H, *J* = 1.8 Hz), 2.90 (s, 2 H), 3.22 (t, 2 H, *J* = 7.2 Hz), 4.11 (t, 2 H, *J* = 1.8 Hz), 4.14 (m, 4 H), 7.30 (m, 5 H), 7.70 (d, 2 H, *J* = 7.8 Hz); ^13^C NMR (100 MHz, CDCl_3_): δ 3.61, 14.25, 16.29, 21.72, 23.07, 23.64, 27.48, 37.11, 45.76, 56.75, 62.20, 76.46, 76.51, 78.09, 80.45, 83.90, 84.08, 123.26, 127.79, 127.86, 128.48, 129.69, 129.75, 131.82, 136.25, 143.63, 168.87; HRMS (ES) m/z calcd for C_33_H_38_NO_6_S (M + H)^+^: 576.2420, found 576.2423 (Δ 0.5 ppm).

#### Diethyl 2-(but-2-ynyl)-2-(3-(2-((but-2-ynyloxy)methyl)phenyl)prop-2-ynyl)malonate (4)


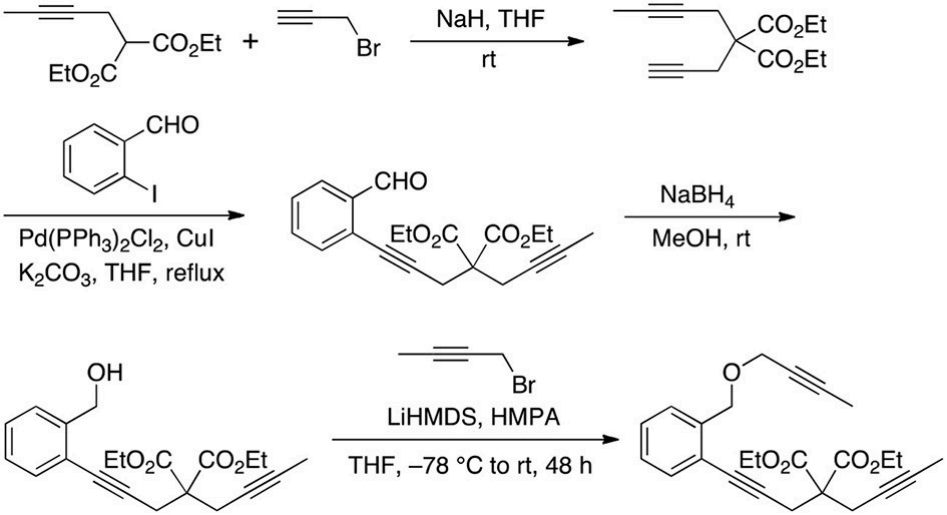


To a mixture of Pd(PPh_3_)_2_Cl_2_ (0.14 g, 0.20 mmol), CuI (76 mg, 0.40 mmol), and K_2_CO_3_ (4.15 g, 30 mmol) in THF (40 mL) were added 2-iodobenzaldehyde (Yoon and Pak, 1973; Grissom et al., 1997) (2.32 g, 10.0 mmol) and a solution of 2-(but-2-ynyl)-2-(prop-2-ynyl)malonate (Murai et al., 2017) (3.75 g, 15.0 mmol) in THF (40 mL). The reaction mixture was stirred at reflux under nitrogen atmosphere overnight. The reaction mixture was filtered through Celite®, and the filtrate was concentrated *in vacuo*. The crude reaction mixture was purified by flash chromatography (silica gel, EtOAc/hexanes = 0% 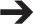
15%) to give diethyl 2-(but-2-ynyl)-2-(3-(2-formylphenyl)prop-2-ynyl)malonate as a light yellow oil (3.18 g, 90%): ^1^H NMR (300 MHz, CDCl_3_) δ 1.25 (t, 6 H, *J* = 7.2 Hz), 1.77 (t,3 H, *J* = 2.4 Hz), 2.97 (q, 2 H, *J* = 2.4 Hz), 3.27 (s, 2 H), 4.24 (q, 4 H, *J* = 7.2 Hz), 7.38–7.55 (m, 3 H), 7.87–7.90 (m, 1 H), 10.45 (d, 1 H, *J* = 0.9 Hz); ^13^C NMR (100 MHz, CDCl_3_) δ 3.72, 14.28, 23.55, 24.04, 57.06, 62.21, 73.10, 79.41, 79.63, 92.20, 127.06, 127.21, 128.62, 133.79, 133.86, 136.40, 169.21, 192.05.

To a solution of diethyl 2-(but-2-ynyl)-2-(3-(2-formylphenyl)prop-2-ynyl)malonate (3.00 g, 8.47 mmol) in MeOH (85 mL) was added sodium borohydride (0.64 g, 16.9 mmol) in one portion, and the reaction mixture was stirred for 1 h until gas evolution stopped. The reaction was quenched with addition of 1 M HCl (85 mL), and the solvent was removed *in vacuo*. The crude reaction mixture was extracted with Et_2_O (50 mL × 3). The combined organic layers were washed with water (50 mL) and brine (50 mL), dried with MgSO_4_, and concentrated *in vacuo*. The crude was purified by flash chromatography (silica gel, EtOAc/hexanes = 10% 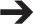
25%) to give diethyl 2-(but-2-ynyl)-2-(3-(2-(hydroxymethyl)phenyl)prop-2-ynyl)malonate as a colorless oil (2.77 g, 92%): ^1^H NMR (300 MHz, CDCl_3_) δ 1.26 (t, 6 H, *J* = 7.2 Hz), 1.77 (t,3 H, *J* = 2.4 Hz), 2.97 (q, 2 H, *J* = 2.4 Hz), 3.22 (s, 2 H), 4.24 (q, 4 H, *J* = 7.2 Hz), 4.73 (s, 2 H), 7.19–7.41 (m, 4H); ^13^C NMR (125 MHz, CDCl_3_) δ 3.70, 14.25, 23.51, 24.07, 57.15, 62.21, 64.23, 73.15, 79.53, 81.46, 89.22, 121.90, 127.64, 128.00, 128.62, 132.67,142.87, 169.56.

A solution of 2-(but-2-ynyl)-2-(3-(2-(hydroxymethyl)phenyl)prop-2-ynyl)malonate (0.71 g, 2.00 mmol) in THF (20 mL) was cooled to−78°C. To this solution, LiHMDS (1 M in hexanes, 2.20 mL, 2.20 mmol) was added dropwise, then HMPA (1.79 g, 10.0 mmol) was added dropwise to the reaction mixture, which was stirred at −78°C for 1 h. 1-Bromobut-2-yne (0.40 g, 6.4 mmol) was added dropwise to the reaction mixture. The reaction mixture was stirred at −78°C for 2 h and warmed to room temperature and stirred for 48 h. The reaction was quenched with addition saturated NH_4_Cl_(aq)_ (20 mL), and the reaction mixture was extracted with Et_2_O (20 mL × 3). The combined organic layers were washed with water (30 mL) and brine (30 mL), dried with MgSO_4_, and concentrated *in vacuo*. The crude was purified by flash chromatography (silica gel, EtOAc/hexanes = 0% 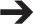 15%) to give **4** as a light yellow oil (0.60 g, 73%): ^1^H NMR (300 MHz, CDCl_3_) δ 1.26 (t, 6 H, *J* = 7.2 Hz), 1.77 (t,3 H, *J* = 2.4 Hz), 1.88 (t,3 H, *J* = 2.4 Hz), 2.98 (q, 2 H, *J* = 2.4 Hz), 3.23 (s, 2 H), 4.19–4.27 (m, 6 H), 4.68 (s, 2 H), 7.16–7.46 (m, 4H); ^13^C NMR (125 MHz, CDCl_3_) δ 3.72, 3.87, 14.29, 23.35, 23.94, 57.17, 58.57, 62.06, 69.91, 73.39, 75.46, 79.31, 81.27, 82.78, 89.16, 121.98, 127.38, 127.68, 128.39, 132.45, 139.85, 169.33; HRMS (ES) m/z calcd for C_25_H_29_O_5_ (M + H)^+^: 409.2015, found 409.2018 (Δ 0.7 ppm).

### Rh-catalyzed [2 + 2 + 2 + 1] cyclocarbonylation reactions

Typical procedures are described here for the reaction of triyne **1b** and **1e**. Other reactions were carried out by using either method as noted.

1. **Procedure A:** Triyne **1b** (0.20 mmol) was introduced to a Schlenck flask, followed by Cl(CH_2_)_2_Cl (2.0 mL, 0.1 M) under nitrogen atmosphere, and then CO was bubbled into the solution at room temperature (**Caution!! Must be done in a well-ventilated hood**). After 15 min, [Rh(COD)Cl]_2_ (0.010 mmol, 5 mol%) was added under CO atmosphere and the resulting mixture was stirred at room temperature for an additional 5 min. Then, the reaction mixture was heated at 50°C with stirring and kept for 16 h under CO (ambient pressure, bubbled into the solution). The reaction mixture was cooled to room temperature and concentrated *in vacuo*. The crude product was purified by flash chromatography (silica gel, EtOAc/hexanes = 10% 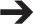
40%) to give **2b** and **3b** (**2b**/**3b** = 20/80) in 95% total yield. The reactions of **1c** and **1d** were carried out under the same conditions.
2. **Procedure B:** Triyne **1f** (0.20 mmol) was introduced to a small round bottomed flask, followed by Cl(CH_2_)_2_Cl (2.0 mL, 0.1 M) under nitrogen atmosphere, and then [Rh(CO)_2_Cl]_2_ (0.010 mmol, 5 mol%) was added. The reaction vessel was placed in a Parr reactor; the Parr reactor was purged and fill with CO gas (2 atm) (**Caution!! Must be done in a well-ventilated hood**). Then, the Parr reactor was heated at 50°C with stirring and kept for 20 h under CO. Upon completion the reaction mixture was cooled to room temperature and concentrated *in vacuo*. The crude product was purified by flash chromatography (silica gel, EtOAc/hexanes = 10% 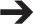 40%) affording **2e** and **3e** (**2f**/**3f** = 70/30) in 96% total yield. The reactions of **1e**, **1g**–**1i** and **4** were carried out under the same conditions.

#### 4,6-dimethyl-2,2,8,8,tetra(carbethoxy)-1,3,7,8-tetrahydro-5-oxo-cyclopenta[*e*]azulene (2b)

Yellow solid; m.p. 78–81°C; ^1^H NMR (300 MHz, CDCl_3_): δ 1.26 (t, 12 H, *J* = 7.2 Hz), 2.23 (s, 6 H), 3.42 (s, 4 H), 3.53 (s, 4 H), 4.21 (q, 8 H, *J* = 7.2 Hz); ^13^C NMR (100 MHz, CDCl_3_): δ 14.0, 19.1, 29.7, 42.3, 43.2, 57.5, 62.1, 138.8, 141.7, 146.6, 170.8, 184.4; HRMS (ES) m/z calcd for C_27_H_35_O_9_ (M + H)^+^: 503.2281, found 503.2271 (Δ 2.0 ppm).

#### 4,5-dimethyl-2,2,7,7-tetra(carbethoxy)-1,3,6,8-tetrahydro-*as*-indacene (3b)

White solid; m.p. 96–98°C; ^1^H NMR (300 MHz, CDCl_3_): δ 1.26 (t, 12 H, *J* = 7.2 Hz), 2.13 (s, 6 H), 3.49 (s, 4 H), 3.50 (s, 4 H), 4.20 (q, 8 H, *J* = 7.2 Hz); ^13^C NMR (100 MHz, CDCl_3_): δ 14.0, 15.7, 39.1, 40.0, 60.0, 61.6, 130.6, 132.2, 138.1, 171.9; HRMS (ES) m/z calcd for C_26_H_35_O_8_ (M + H)^+^: 475.2332, found 475.2338 (Δ 1.3 ppm).

#### 4,6-dimethyl-1,3,7,8-tetrahydro-5-oxo-2,8-dioxa-cyclopenta[*e*]azulene (2c)

Yellow solid; turn brown at ~ 200°C m.p. 229–231°C; ^1^H NMR (300 MHz, CDCl_3_): δ 2.17 (s, 6 H), 4.85 (s, 4 H), 5.02 (s, 4 H); ^13^C NMR (100 MHz, CDCl_3_): δ 18.1, 73.6, 75.0, 135.0, 140.0, 146.2, 182.9; HRMS (ES) m/z calcd for C_13_H_15_O_3_ (M + H)^+^: 219.1021, found 219.1013 (Δ 3.7 ppm).

#### 4,5-dimethyl-1,3,6,8-tetrahydro-2,7-dioxa-*as*-indacene (3c)

Yellow solid; turn brown at ~ 200°C m.p. 212–214°C; ^1^H NMR (300 MHz, CDCl_3_): δ 2.15 (s, 6 H), 5.04 (s, 4 H), 5.09 (s, 4 H); ^13^C NMR (100 MHz, CDCl_3_): δ 15.4, 72.9, 73.4, 128.6, 128.8, 138.2; HRMS (ES) m/z calcd for C_13_H_15_O_2_ (M + H)^+^: 191.1072, found 191.1066 (Δ 3.1 ppm). All data are in agreement with those reported in the literature (Yamamoto et al., 2003; Geny et al., 2009).

#### 4,6-dimethyl-8,8-di(carbethoxy)-1,3,7,8-tetrahydro-2-(4-methylbenzenesulfonyl)-5-oxo-2-aza-cyclopenta[*e*]azulene (2d)

Yellow solid; m.p. 82–85°C; ^1^H NMR (300 MHz, CDCl_3_): δ 1.26 (t, 6 H, *J* = 7.2 Hz), 2.11 (s, 3 H), 2.22 (s, 3 H), 2.42 (s, 3 H), 3.29 (s, 2 H), 3.50 (s, 2 H), 4.21 (q, 4 H, *J* = 7.2 Hz), 4.29 (s, 2 H), 4.46 (s, 2 H), 7. 35 (d, 2 H, *J* = 8.1 Hz), 7.76 (d, 2 H, *J* = 8.1 Hz); ^13^C NMR (100 MHz, CDCl_3_): δ 14.0, 18.4, 19.2, 21.5, 41.6, 43.1, 54.6, 55.3, 57.6, 62.2, 127.7, 130.0, 132.7, 134.3, 138.4, 140.7, 142.4, 143.0, 144.3, 146.7, 170.5, 183.6; HRMS (ES) m/z calcd for C_27_H_32_NO_7_S (M + H)^+^: 514.1899, found 514.1897 (Δ 0.4 ppm).

#### 4,5-dimethyl-7,7-di(carbethoxy)-1,3,6,8-tetrahydro-2-(4-methylbenzenesulfonyl)-2-aza-*as*-indacene (3d)

White solid; turn brown at ~ 138°C m.p. 144–146°C; ^1^H NMR (300 MHz, CDCl_3_): δ 1.25 (t, 6 H, *J* = 7.2 Hz), 2.06 (s, 3 H), 2.12 (s, 3 H), 2.40 (s, 3 H), 3.40 (s, 2 H), 3.48 (s, 2 H), 4.19 (q, 4 H, *J* = 7.2 Hz), 4.52 (bs, 4 H), 7. 31 (d, 2 H, *J* = 8.1 Hz), 7.77 (d, 2 H, *J* = 8.1 Hz); ^13^C NMR (100 MHz, CDCl_3_): δ 14.0, 15.6, 21.5, 38.8, 39.8, 53.0, 53.6, 60.0, 61.8, 127.5, 128.5, 129.5, 129.8, 131.0, 131.7, 133.9, 134.5, 139.2, 143.6, 171.5; HRMS (ES) m/z calcd for C_26_H_32_NO_6_S (M + H)^+^: 486.1950, found 486.1947 (Δ 0.6 ppm).

#### 4,6-dimethyl-2,2,9,9-tetra(carbethoxy)-1,3,7,8,9-pentahydro-5-oxo-benzo[*e*]azulene (2e)

Light yellow oil; ^1^H NMR (300 MHz, CDCl_3_): δ 1.21–1.28 (m, 14H), 2.19–2.23 (m, 8H), 2.69 (t, 2H, *J* = 6.6 Hz), 3.04 (s, 2H), 3.45 (s, 2H), 3.61 (s, 2H), 4.15–4.24 (m, 8H); ^13^C NMR (100 MHz, CDCl_3_) δ 13.97, 18.61, 18.68, 27.83, 28.36, 35.84, 41.98, 42.20, 53.42, 56.71, 61.70, 61.94, 134.58, 139.61, 140.84, 141.12, 143.1, 144.66, 170.95, 171.30, 188.19. HRMS (ES) m/z calcd for C_28_H_37_O_9_ (M + H)^+^: 517.2432, found 517.2440 (Δ 1.5 ppm).

#### 4,5-dimethyl-2,2,8,8-tetra(carbethoxy)-3,6,7,8,9-pentahydro-1*H*-cyclopenta[*a*]naphthalene (3e)

Light yellow oil; ^1^H NMR (300 MHz, CDCl_3_): δ 1.21–1.28 (m, 12 H), 2.07 (s, 3 H), 2.15 (s, 3 H), 2.31 (t, 2 H, *J* = 6.6 Hz), 2.67 (t, 2 H, *J* = 6.6 Hz), 3.11 (s, 2 H), 3.53 (s, 2 H), 3.54 (s, 2 H), 4.14–4.24 (m, 8 H); ^13^C NMR (100 MHz, CDCl_3_) δ 14.01, 14.7, 16.29, 24.44, 28.17, 32.51, 39.3, 40.16, 52.92, 59.44, 61.36, 61.62, 126.55, 129.56, 131.62, 133.18, 135.19, 136.37, 171.40, 171.92; HRMS (ES) m/z calcd for C_27_H_40_NO_8_ (M + NH_4_)^+^: 506.2748, found 506.2753 (Δ 0.8 ppm).

#### 4-phenyl-6-methyl-2,2,9,9-tetra(carbethoxy)-1,3,7,8,9-pentahydro-5-oxo-benzo[*e*]azulene (2f)

Light yellow oil; ^1^H NMR (300 MHz, CDCl_3_): δ 1.23 (m, 12 H), 2.20 (s, 3 H), 2.26 (t, 2 H, *J* = 6.3 Hz), 2.71 (t, 2 H, *J* = 6.3 Hz), 3.08 (s, 2 H), 3.13 (s, 2 H), 3.60 (s, 2 H), 4.18 (m, 8 H), 7.24–7.40 (m, 5 H); ^13^C NMR (100 MHz, CDCl_3_): δ 13.90, 13.98, 18.47, 27.77, 28.18, 35.80, 41.73, 42.98, 53.47, 57.08, 61.75, 61.83, 127.40, 128.03, 129.10, 135.82, 138.81, 14.046, 141.61, 142.76, 143.01, 144.93, 170.71, 171.27, 187.14; HRMS (ES) m/z calcd for C_33_H_39_O_9_ (M + H)^+^: 579.2589, found 579.2593 (Δ 0.7 ppm).

#### 4-phenyl-5-methyl-2,2,8,8-tetra(carbethoxy)-3,6,7,8,9-pentahydro-1*H*-cyclopenta[*a*]naphthalene (3f)

Light yellow oil; ^1^H NMR (300 MHz, CDCl_3_): δ 1.23 (m, 12 H), 1.92 (s, 3 H), 2.36 (t, 2 H, *J* = 6.6 Hz), 2.68 (t, 2 H, *J* = 6.6 Hz), 3.18 (s, 2 H), 3.27 (s, 2 H), 3.57 (s, 2 H), 4.18 (m, 8 H), 7.16–7.19 (m, 2 H), 7.31–7.43 (m, 3 H); ^13^C NMR (100 MHz, CDCl_3_): δ 13.98, 14.02, 16.32, 24.37, 28.05, 32.59, 39.35, 40.47, 52.96, 59.72, 61.44, 61.56, 126.61, 128.25, 128.38, 129.23, 132.08, 132.64, 135.35, 136.11, 136.18, 140.52, 171.41, 171.75; HRMS (ES) m/z calcd for C_32_H_42_NO_8_ (M + NH_4_)^+^: 568.2905, found 568.2912 (Δ 1.3 ppm).

#### 4-phenyl-6-methyl-2,2-di(carbethoxy)-9-(4-methylbenzenesulfonyl)-1,3,7,8,9-pentahydro-5-oxo-9-aza-benzo[*e*]azulene (2 g)

Light yellow oil; ^1^H NMR (300 MHz, CDCl_3_): δ 1.21 (t, 6 H, *J* = 7.2 Hz), 2.04 (s, 2 H), 2.10 (s, 3 H), 2.43 (s, 3 H), 2.77 (t, 2 H, *J* = 6.3 Hz), 3.12 (s, 2 H), 3.38–3.42 (m, 4 H), 4.17 (q, 4 H, *J* = 7.2 Hz), 7.22–7.25 (m, 2 H), 7.28–7.42 (m, 5 H), 7.65–7.69 (m, 2 H); ^13^C NMR (100 MHz, CDCl_3_) δ 14.27, 18.83, 21.86, 30.51, 41.08, 43.10, 43.56, 48.72, 57.65, 62.39, 127.87, 128.03, 128.40, 129.45, 130.12, 133.81, 134.04, 138.45, 138.55, 139.23, 142.29, 144.1, 144.58, 144.72, 170.79, 187.07; HRMS (ES) m/z calcd for C_33_H_36_NO_7_S (M + H)^+^: 590.2212, found 590.2201 (Δ 1.9 ppm).

#### 4-phenyl-5-methyl-2,2-di(carbethoxy)-8-(4-methylbenzenesulfonyl)-3,6,7,8,9-pentahydro-8-aza-1*H*-cyclopenta[*a*]naphthalene (3g)

Light yellow oil; ^1^H NMR (300 MHz, CDCl_3_): δ 1.22 (t, 6 H, *J* = 7.2 Hz), 1.91 (s, 3 H), 2.44 (s, 3 H), 2.81 (t, 2 H, *J* = 5.7 Hz), 3.24 (s, 2 H), 3.37 (t, 2 H, *J* = 5.7 Hz), 3.45 (s, 2 H), 4.13 (s, 2 H), 4.16 (q, 4 H, *J* = 7.2 Hz), 7.10–7.14 (m, 2 H), 7.26–7.43 (m, 5 H), 7.74–7.76 (m, 2 H). ^13^C NMR (100 MHz, CDCl_3_) δ 13.98, 16.12, 21.51, 27.36, 38.59, 40.20, 43.69, 46.06, 59.84, 61.75, 126.61, 126.90, 127.83, 128.41, 129.02, 129.70, 130.76, 133.00, 133.56, 136.84, 137.02, 140.00, 143.9, 171.53; HRMS (ES) m/z calcd for C_32_H_39_N_2_O_6_S (M + NH_4_)^+^: 579.2523, found 579.2527 (Δ 0.6 ppm).

#### 10,10-di(carbethoxy)-6,8-dimethyl-7-oxo-2-(4-methylbenzenesulfonyl)-2,3,4,5,9,11-hexahydro-1*H*-azuleno[4,5-*c*]azepine (2h)

Light yellow oil; ^1^H NMR (300 MHz, CDCl_3_): δ 1.26 (t, 6 H, *J* = 7.2 Hz), 1.62 (m, 2 H), 1.97 (s, 3 H), 2.14 (s, 3 H), 2.37 (s, 3 H), 2.61 (m, 2 H), 3.38 (s, 2 H), 3.57 (t, 2 H, *J* = 5.7 Hz), 3.62 (s, 2 H), 4.20 (q, 4 H, *J* = 7.2 Hz), 4.48 (s, 2 H), 7.07 (d, 2 H, *J* = 8.1 Hz), 7.76 (d, 2 H, *J* = 8.1 Hz); ^13^C NMR (100 MHz, CDCl_3_) δ 14.15, 17.96, 18.78, 21.58, 26.55, 33.07, 41.38, 41.84, 50.61, 52.29, 57.09, 62.24, 126.81, 129.76, 136.71, 136.78, 140.07, 140.11, 141.89, 143.74, 144.37, 145.69, 171.01, 189.41; HRMS (ES) m/z calcd for C_29_H_36_NO_7_S (M + H)^+^: 542.2212, found 542.2215 (Δ 0.6 ppm).

#### 6,7-dimethyl-9,9-di(carbethoxy)-2-(4-methylbenzenesulfonyl)-2,3,4,5,8,10-hexahydroindeno[4,5-*c*]azepine (3h)

Light yellow oil; ^1^H NMR (300 MHz, CDCl_3_): δ 1.27 (t, 6 H, *J* = 7.2 Hz), 1.62 (m, 2 H), 2.11 (s, 3 H), 2.18 (s, 3 H), 2.90 (s, 3 H), 2.80 (m, 2 H), 3.46 (t, 2 H, *J* = 5.7 Hz), 3.55 (s, 2 H), 3.75 (s, 2 H), 4.20 (q, 4 H, *J* = 7.2 Hz), 4.36 (s, 2 H), 7.19 (d, 2 H, *J* = 8.4 Hz), 7.52 (d, 2 H, *J* = 8.4 Hz); ^13^C NMR (100 MHz, CDCl_3_) δ 14.20, 15.76, 17.29, 21.66, 26.88, 28.71, 39.61, 40.49, 49.39, 51.35, 59.61, 61.92; HRMS (ES) m/z calcd for C_28_H_36_NO_6_S (M + H)^+^: 514.2263, found 514.2260 (Δ 0.6 ppm).

#### 10,10-di(carbethoxy)-6-methyl-8-phenyl-7-oxo-2-(4-methylbenzenesulfonyl)-2,3,4,5,9,11-hexahydro-1*H*-azuleno[4,5-*c*]azepine (2i)

Light yellow oil; ^1^H NMR (300 MHz, CDCl_3_): δ 1.23 (t, 6 H, *J* = 7.2 Hz), 1.67 (m, 2 H), 1.94 (s, 3 H), 2.40 (s, 3 H), 2.65 (m, 2 H), 3.09 (s, 2 H), 3.64 (t, 2 H, *J* = 5.7 Hz), 3.68 (s, 2 H), 4.18 (q, 4 H, *J* = 7.2 Hz), 4.54 (s, 2 H), 7.13 (d, 2 H, *J* = 7.8 Hz),7.26–7.42 (m, 7 H); ^13^C NMR (100 MHz, CDCl_3_) δ 14.17, 17.85, 21.70, 26.66, 33.04, 41.13, 42.80, 50.94, 52.13, 57.42, 62.17, 126.85, 127.84, 128.28, 129.38, 129.87, 136.88, 137.86, 138.55, 139.65, 142.11, 143.70, 143.93, 144.55, 145.19, 170.89, 188.152; HRMS (ES) m/z calcd for C_34_H_38_NO_7_S (M + H)^+^: 604.2369, found 604.2368 (Δ 0.2 ppm).

#### 6-methyl-7-phenyl-9,9-di(carbethoxy)-2-(4-methylbenzenesulfonyl)-2,3,4,5,8,10-hexahydroindeno[4,5-*c*]azepine (3i)

Light yellow oil; ^1^H NMR (300 MHz, CDCl_3_): δ 1.22 (t, 6 H, *J* = 7.2 Hz), 1.66 (m, 2 H), 1.93 (s, 3 H), 2.40 (s, 3 H), 2.84 (m, 2 H), 3.25 (s, 2 H), 3.54 (t, 2 H, *J* = 5.7 Hz), 3.82 (s, 2 H), 4.16 (q, 4 H, *J* = 7.2 Hz), 4.44 (s, 2 H), 7.15–7.24 (m, 4 H), 7.27–7.55 (m, 5 H); ^13^C NMR (100 MHz, CDCl_3_) δ14.20, 17.32, 21.70, 26.63, 28.98, 39.64, 40.79, 49.40, 51.76, 59.93, 61.89, 127.03, 127.40, 128.65, 129.19, 129.67, 132.07, 132.49, 136.28, 136.82, 137.21, 138.66, 139.45, 140.99, 143.14, 171.94; HRMS (ES) m/z calcd for C_33_H_38_NO_6_S (M + H)^+^: 576.2420, found 576.2424 (Δ 0.7 ppm).

#### 8,8-di(carbethoxy)-4,6-dimethyl-5-oxo-2,3,7,9-tetrahydro-1*H*-azuleno[5,4-*c*]benzo[*e*]oxepine (5)

Off-white solid: mp 142.5–143.5°C; ^1^H NMR (300 MHz, CDCl_3_) δ 1.12–1.35 (m, 6 H), 2.26(s, 3H), 2.37 (s, 3 H), 2.94 (bs, 1 H), 3.37 (bs, 1 H), 3.49 (s, 2 H), 3.88 (bs, 1 H), 4.02–4.26 (m, 4 H), 4.55 (s, 2 H), 4.70 (bs, 1 H), 7.11–7.16 (m, 1 H), 7.31–7.43 (m, 3 H); ^13^C NMR (100 MHz, CDCl_3_) δ 14.13, 18.59, 19.79, 42.36, 44.82, 57.96, 62.13, 65.28, 68.11, 128.75, 128.95, 129.05, 129.23, 132.70, 138.07, 140.01, 140.29, 141.17, 142.01, 142.06, 145.68, 170.80, 190.85; LR-MS *m/z* calcd for C_26_H_28_O_6_ (M^+^) 436.5, found (M+ 1): 437.1; HRMS (ES) m/z calcd for C_26_H_29_O_6_ (M + H)^+^: 437.1964, found 437.1966 (Δ 0.5 ppm).

#### 11,11-di(carbethoxy)-8,9-dimethyl-7,10,11,12-tetrahydro-5*H*-benzo[*e*]indeno[5,4-*c*]oxepine (6)

White solid: mp 125.0–126.0°C; ^1^H NMR (300 MHz, CDCl_3_) δ 1.17 (t, 3 H, *J* = 7.2 Hz), 1.30 (t, 3 H, *J* = 7.2 Hz), 2.29 (s, 3 H), 2.38 (s, 3 H), 3.32 (d, 1 H, *J* = 16.5 Hz), 3.57 (d, 1 H, *J* = 16.2 Hz), 3.71 (d, 1 H, *J* = 16.5 Hz), 3.83 (d, 1 H, *J* = 11.7 Hz), 4.02–4.30 (m, 6 H), 4.45 (d, 1 H, *J* = 11.1 Hz), 4.82 (d, 1 H, *J* = 11.7 Hz), 7.36–7.57 (m, 4 H); ^13^C NMR (100 MHz, CDCl_3_) δ 14.15, 14.31, 15.71, 17.14, 40.49, 40.74, 60.10, 61.87, 62.02, 62.88, 67.51, 128.09, 128.21, 128.34, 129.71, 132.67, 132.71, 134.31, 134.46, 134.82, 135.37, 140.03, 140.25, 171.79, 172.08; LR-MS *m/z* calcd for C_25_H_28_O_5_ (M^+^) 408.5, found (M+ 1): 409.2; HRMS (ES) m/z calcd for C_25_H_29_O_5_ (M + H)^+^: 409.2015, found 409.2015 (Δ 0.0 ppm).

## Results and discussion

**Scheme 3:**
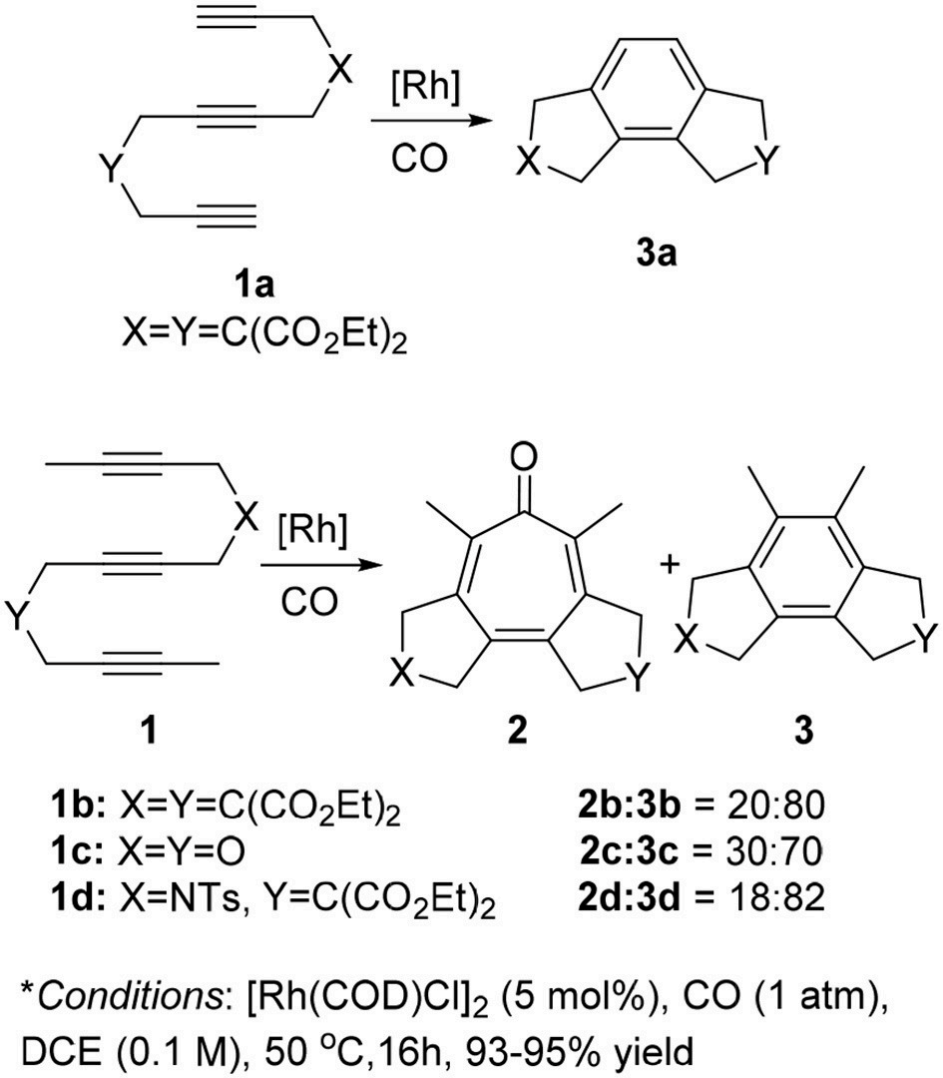
First carbonylative [2+2+2+1] cycloaddition of triynes **1a–d** and CO, forming tropones **2b–d**.

First, we investigated the reaction of 1,6,11-triynes **1a–d** with CO under the typical conditions for the [2+2+2+1] cycloaddition of enediynes (Bennacer et al., 2004, 2005) i.e., [Rh(COD)Cl]_2_ catalyst, 1 atm of CO in dichloroethane (DCE) at 50°C (Scheme 3). At first, the terminal triyne **1a** was subjected to cycloaddition, but the formation of tropone **2a** was not observed at all, while tricyclic benzene derivative **3a** was obtained exclusively.

Gratifyingly, the reaction of internal triynes **1b–d** gave tricyclic tropones **2b–d** as minor products through carbonylative [2+2+2+1] cycloaddition together with anticipated tricyclic benzene derivatives **3b–d** through [2+2+2] cycloaddition as major products. Following up this encouraging result, we attempted to increase the selectivity of the tropone formation through optimization of reaction variables (i.e., solvents, CO pressure, use of Mn(CO)_6_, etc.), as well as terminal and internal substituents in the 1,6,11-triynes, but without success.

**Scheme 4:**
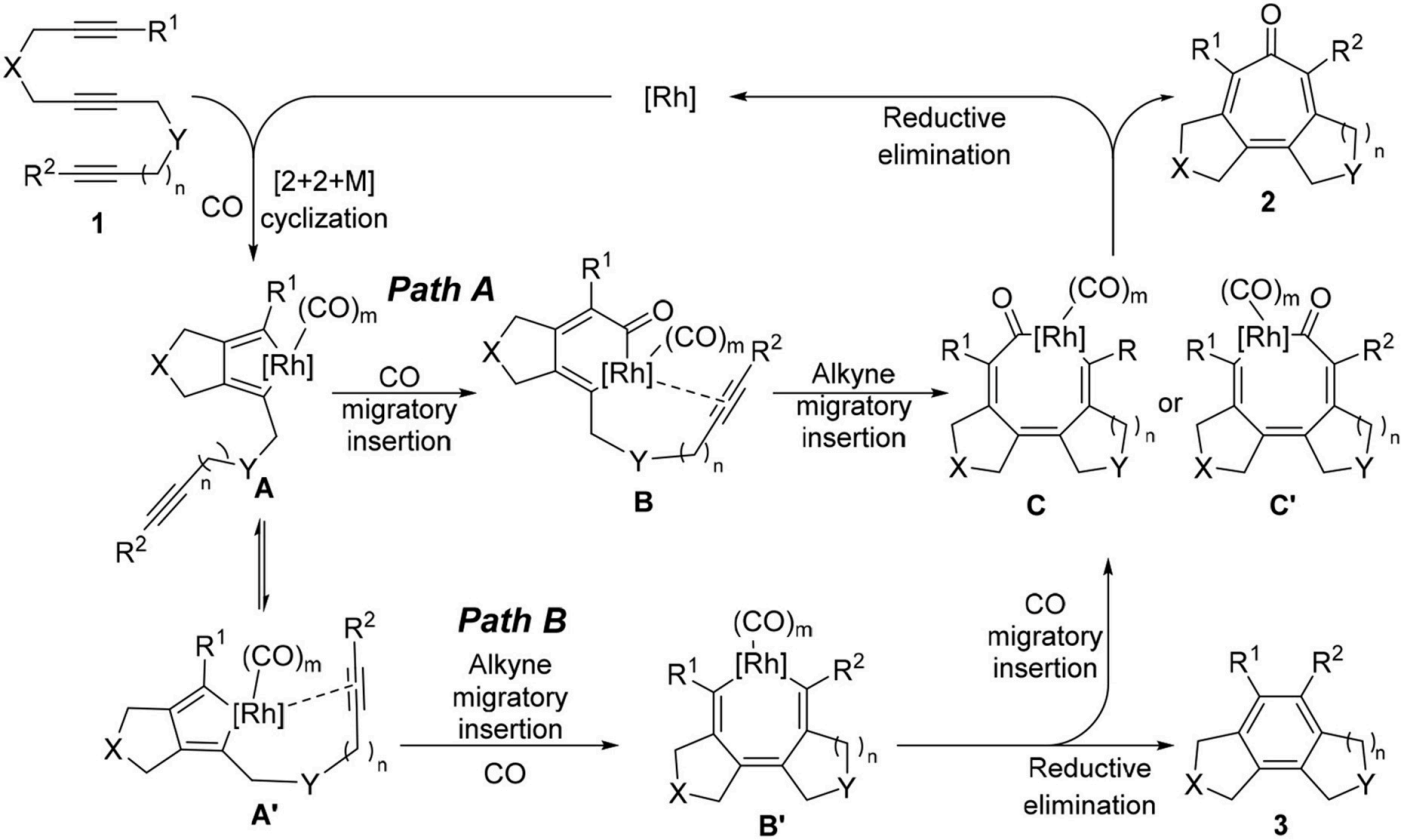
Proposed carbonylative [2+2+2+1] cycloaddition mechanism.

Therefore, we turned our attention to the mechanistic analysis the reaction pathways based on the proposed mechanism for the [2+2+2+1] cycloaddition of 1,6,11-enediynes (Scheme 4) (Bennacer et al., 2004, 2005). If we follow the previously proposed [2+2+2+1] mechanism for *enediynes*, the cycloaddition proceeds through ***Path B***: first, oxidative cyclization to form metalacyclopentadiene **A** or **A'**, followed by the second cyclization to give metalacycle **B'**. However, in this case, i.e., *triynes*, the formation of tricyclic benzene **3** is obviously favored through facile reductive elimination from metalacycle **B'** rather than CO insertion to metalacycle **B**' to give metalacyclooctatrienone **C** or **C'**.

Thus, we have hypothesized an alternative mechanism, which involves metalacyclohexadienone **B** as the key intermediate through ***Path A***, prior to the insertion of the third acetylene moiety. It is reasonable to assume that the introduction of a longer tether between the second and third acetylene moieties, equilibrium between rhodacyclopentadiene **A** and **A**′ may favor the CO insertion to rhodacyclopentadiene **A** to form **B**, leading to the specific formation of **C**, which should lead to the formation of tropone product **2**.

**Figure 1:**
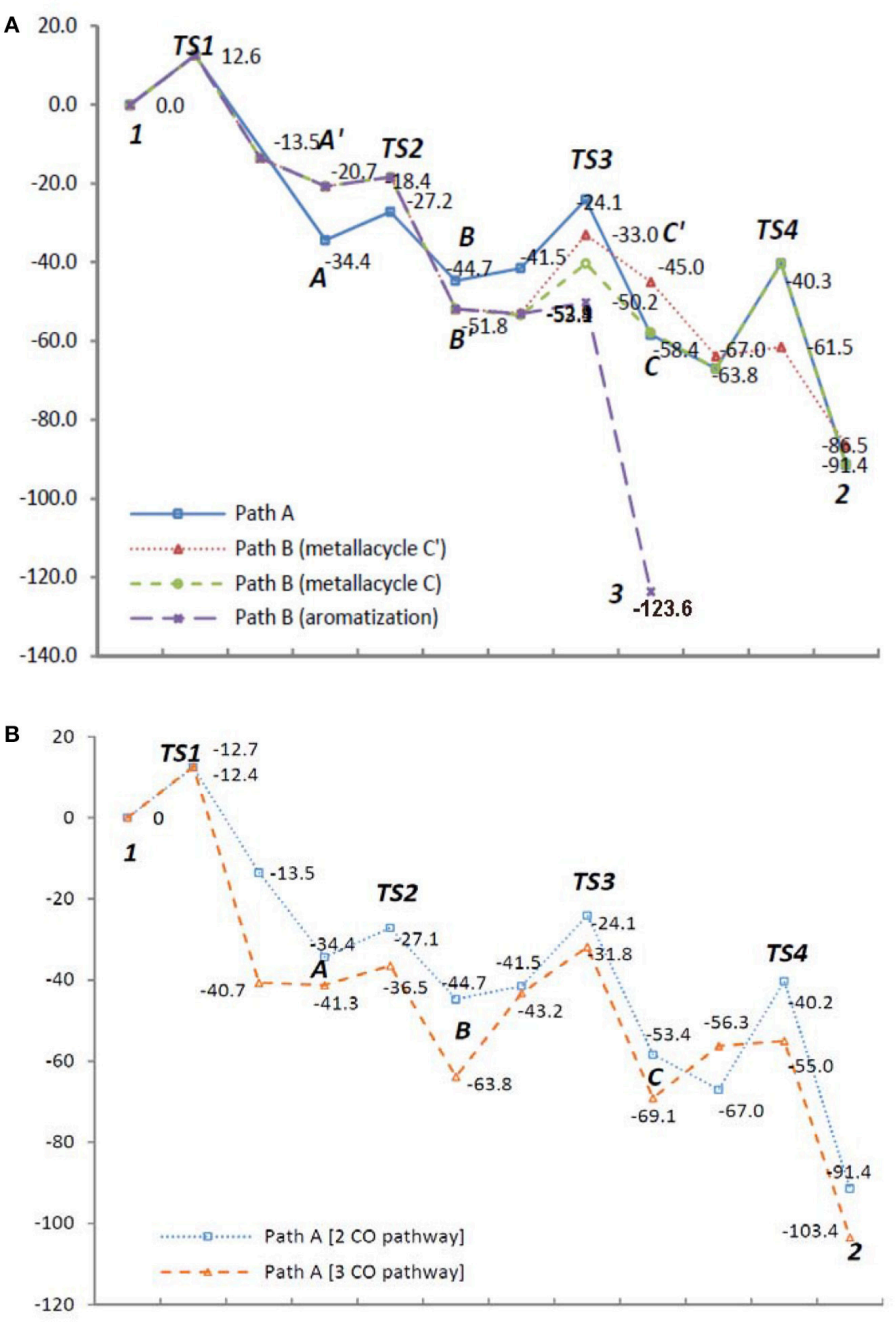
Energy diagrams (Kcal/mol) of **(A)** reaction Path A and B with Rh(CO)_2_ species; **(B)** reaction Path A with Rh(CO)_2_ and Rh(CO)_3_ species.

To examine the feasibility of the proposed mechanism, all proposed pathways ***A*** and ***B*** were analyzed by DFT calculations (Gaussian 09, B3LYP, base sets: LANL2DZ for metal atom, 6-31++G^**^ for non-metal atoms) for 1,6,12-triyne **1** (*n* = 2) involving Rh(CO)_2_ species in each Rh intermediate for consistency (See Supplementary Data Sheets 1, 2). Results are shown in Figure 1 (energy unit: Kcal/mol).

Intermediates **A** and **A**′ are conformers; from the transition state *TS1* if the reaction proceeds via intermediate A′, it's clear that it would greatly favor the formation of the benzene product **3**. However, if *TS1* gives more stable intermediate **A**, it would proceed via CO insertion to give intermediate **B** rather than isomerizes to **A**′ due to less activation energy, and ultimately gives tropone product **2** (Figure 1A). When the Rh(CO)_3_ species are introduced to the reaction ***Path A***, its DFT energy profile is more favorable than that of the Rh(CO)_2_ species in the same pathway (Figure 1B). For the DFT analysis of ***Path A*** and ***Path B*** with chemical structures of the intermediates and transition states as well as their coordinates, see Supporting Information.

To confirm the prediction based on the DFT calculations, we prepared 1,6,12-triynes (*n* = 2) **1e–g** as well as 1,6,13-triynes (*n* = 3) **1h** and **1i** and subjected them to the reaction conditions using [Rh(CO)_2_Cl]_2_ as the catalyst at 50°C in dichloroethane (DCE) under 2 atm of CO. Results are summarized in Table 1. As Table 1 shows, the selectivity for tropone formation via carbonylative [2+2+2+1] cycloaddition was indeed substantially improved and thus tropones **2e–i** became the major products in these reactions.

**Table 1:** Higher-order cycloaddition of 1,6,12- and 1,6,13-triynes.

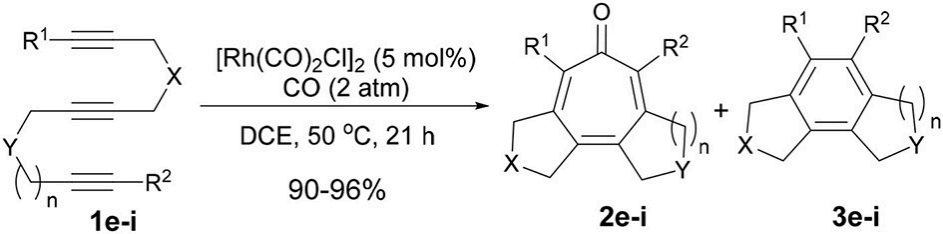								
**Entry**	**Triyne**	**X**	**Y**	***R***^1^	***R***^2^	***n***	**2:3**	**2:3 (Isolated yield)**
1	**1e**	C(CO_2_Et)_2_	C(CO_2_Et)_2_	Me	Me	2	50:50	45%: 45%
2	**1f**	C(CO_2_Et)_2_	C(CO_2_Et)_2_	Ph	Me	2	70:30	67%: 29%
3	**1g**	C(CO_2_Et)_2_	NTs	Ph	Me	2	58:42	54%: 39%
4	**1h**	C(CO_2_Et)_2_	NTs	Me	Me	3	58:42	53%: 38%
5	**1i**	C(CO_2_Et)_2_	NTs	Ph	Me	3	67:33	62%: 30%

Introduction of a phenyl group as R^1^ has a favorable effect on the carbonylative [2+2+2+1] cycloaddition, but there is no difference between these two tether lengths (*n* = 2 vs. *n* = 3). It is noteworthy that 5-7-7 fused tricyclic products, **2h** and **2i**, were formed in fairly good isolated yields. The reaction that affords a 7-7 fused ring system in one-step is hereto unknown in the literature. Thus, this is the first reaction that achieved such a challenging process. At this point, we envisioned that the insertion of a 1,2-disubstituted benzene unit to the triyne substrate might introduce a tether with more rigid constraints than triynes **1h** and **1i** to favor the ***Path A***, hence the formation of tropone.

**Scheme 5:**
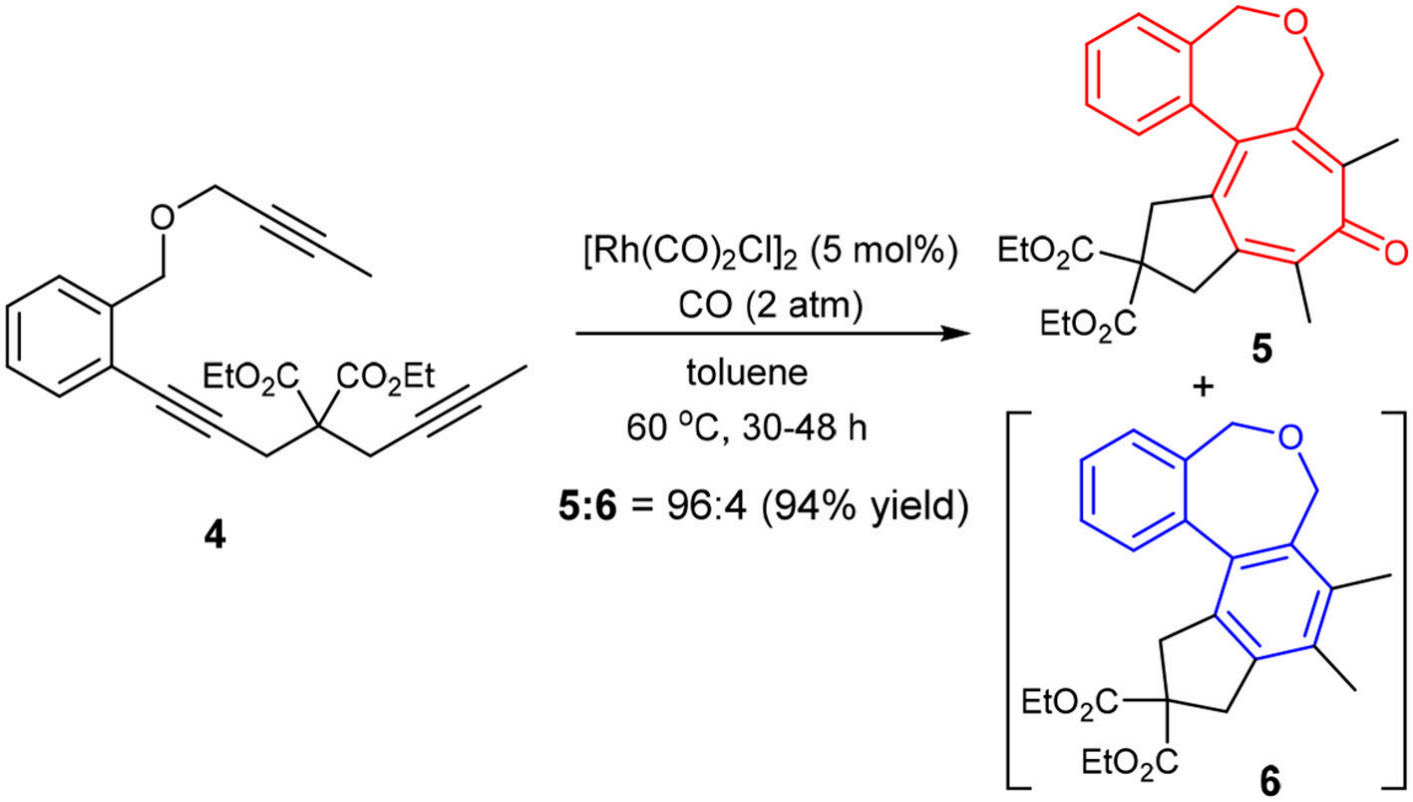
Fused tetracyclic tropone synthesis through carbonylative [2+2+2+1] cycloaddition of **4**.

Thus, we prepared triynes **4** and subjected to the Rh-catalyzed higher-order cycloaddition conditions, using [Rh(CO)_2_Cl]_2_ (5 mol%) as the catalyst under 2 atm of CO. The reaction of **4** in toluene at 60 °C for 48 h gave the corresponding 6-7-7-5 fused tetracyclic cycloaddition product **5** with 96% selectivity accompanied by only 4% of **6** in 94% combined yield (Scheme 5).

It is rational to ascribe the observed unexpectedly high selectivity for [2+2+2+1] cycloaddition to the rotational restriction by the introduction of a 1,2-disubstituted benzene unit to the tether connecting the second and third acetylene moieties, which disfavored the ***Path B*** and favored the ***Path A*** (see Scheme 4).

**Figure 2:**
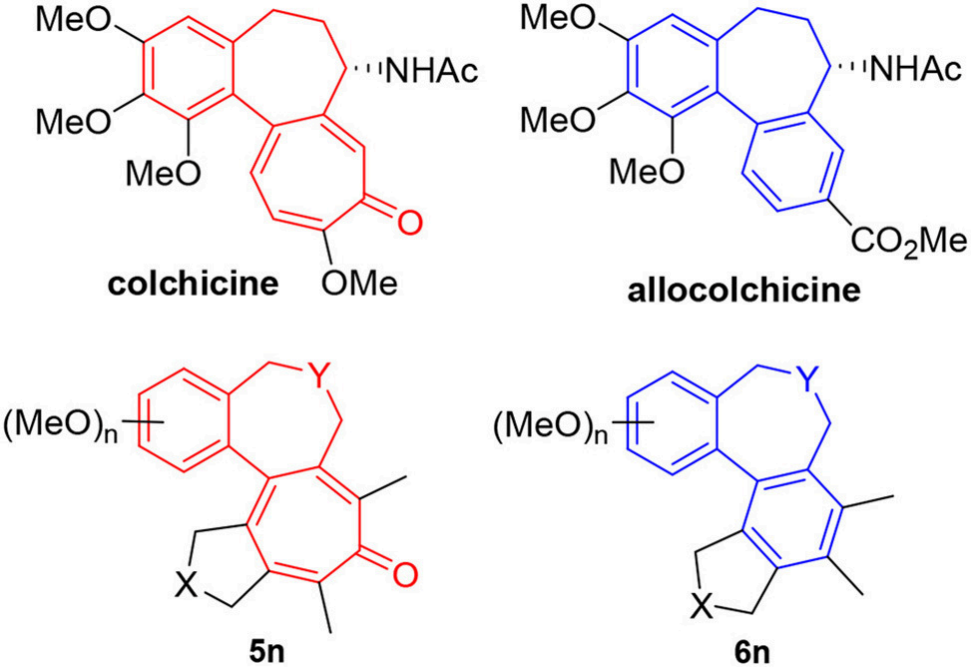
Colchicine, allocolchicine and colchicinoids.

We recognized that the fused tetracyclic products **5** and **6** mimic the colchicine and allocolchicine skeletons, respectively (Figure 2). It is worthy of note that the rapid construction of colchicinoid skeleton is realized through novel [2+2+2+1] cycloaddition of triynes and CO in one-step. Further investigations into the scope and limitation of the [2+2+2+1] and [2+2+2] cycloaddition of triynes are actively underway in our laboratory and will be published in due course.

## Conclusions

The first carbonylative [2+2+2+1] cycloaddition of triynes of 1,6,n-triynes (*n* = 11–13) with CO was achieved by the catalysis of a Rh complex. This [2+2+2+1] cycloaddition process (**Path B**) should be energetically unfavorable than the competing [2+2+2] cycloisomerization process if the CO insertion occurs after the formation of metalacyclooctatriene intermediate since a simple reductive elimination gives the corresponding aromatized product, i.e., benzene derivative. Thus, the CO insertion step in the [2+2+2+1] cycloaddition process should involve a carbonylated diene species prior to the reaction with the third acetylene moiety. This analysis led to the proposal of a feasible mechanism for this novel [2+2+2+1] cycloaddition process, involving a rhodacyclpentadienone species **B** as the key intermediate (**Path A**). The DFT calculations of all key intermediates and transition states clearly supported the proposed mechanism. Based on this mechanism, a triyne substrate **4** was designed, in part inspired by the framework of colchicine, a naturally occurring bioactive tropone. The introduction of a 1,2-disubstitute benzene as a tether to the third acetylene unit should slow down the coordination of the acetylene and favor the CO insertion to the metalacyclopentadiene intermediate **A** to form the key intermediate **B**, leading to the formation of tropone **2**. In fact, the reaction of **4** afforded the corresponding fused tetracyclic tropone **5** in 94% yield and 96% selectivity. Since this novel process is applicable to the design and synthesis of various colchicinoids, further studies on this process and applications are actively underway in our laboratory.

## Author contributions

Y-HT designed and performed major experiments, as well as collected characterization data and carried out preliminary DFT calculations. C-WC also performed experiments and collected characterization data. W-HC carried out DFT calculations and validated results. TH organized manuscripts and validated data. IO oversaw all aspects of the research, including experimental designs, analysis of mechanism and overall organization of the manuscript.

### Conflict of interest statement

The authors declare that the research was conducted in the absence of any commercial or financial relationships that could be construed as a potential conflict of interest.

## Supplementary material

Supplementary Data Sheet 1 Energy diagrams based on DFT calculations with chemical structures for intermediates and transition states, ^1^H and ^13^C NMR spectra of triynes and fused tropone and benzene products.

Supplementary Data Sheet 2 Coordinates for all key intermediates involved in the energy diagrams.
