# Supplementary material for: Construction of Fused Tropone Systems Through Intramolecular Rh(I)-Catalyzed Carbonylative [2+2+2+1] Cycloadditon of Triynes
Source: Front Chem. 2018 Sep 10;6:401. doi: 10.3389/fchem.2018.00401 (PMC6139344; doi:10.3389/fchem.2018.00401)

Supporting Information

Rapid Construction of Fused Tropone Systems Through Intramolecular Rh(I)-Catalyzed Carbonylative [2+2+2+1] Cycloaddition of Triyens

Yu-Han G. Teng,[1] Chih-Wei Chien,[1] Wen-Hua Chiou,[2] Tadashi Honda[1] and Iwao Ojima[1,*]

[1]Department of Chemistry, Stony Brook University, Stony Brook, NY 11794-3400, USA
[2]Department of Chemistry, National Chung Hsing University, Taichung 402, Taiwan

iwaoojima@stonybrpook.edu

Contents

DFT Calculations S1
Figure S1, Figure S2 .... S2
Figure S3, Figure S4 .... S3
Figure S5 .... S4
NMR Spectra S5-S56

DFT Calculations

All calculations were performed at the level of B3LYP, and using the LanL2DZ basis set for the metal atom and the $631^{++}G^{**}$ for nonmetal atom in *Gaussian* 09. Stationary points, i.e. reactants and products, were achieved by IRC calculations of the corresponding transition states, followed by geometry optimization. All geometries have been confirmed as either stationary points or saddle points by vibrational analyses at the same level. Thermal corrections were calculated at 1 atm 298.15 K in the gas phase.

The coordinates for all chemical structures for intermediates and transition states for Figure 1A (Figure S1-S3) and Figure 1B (Figure S4 and S5) are provided in two additional and separate Supporting Information files.

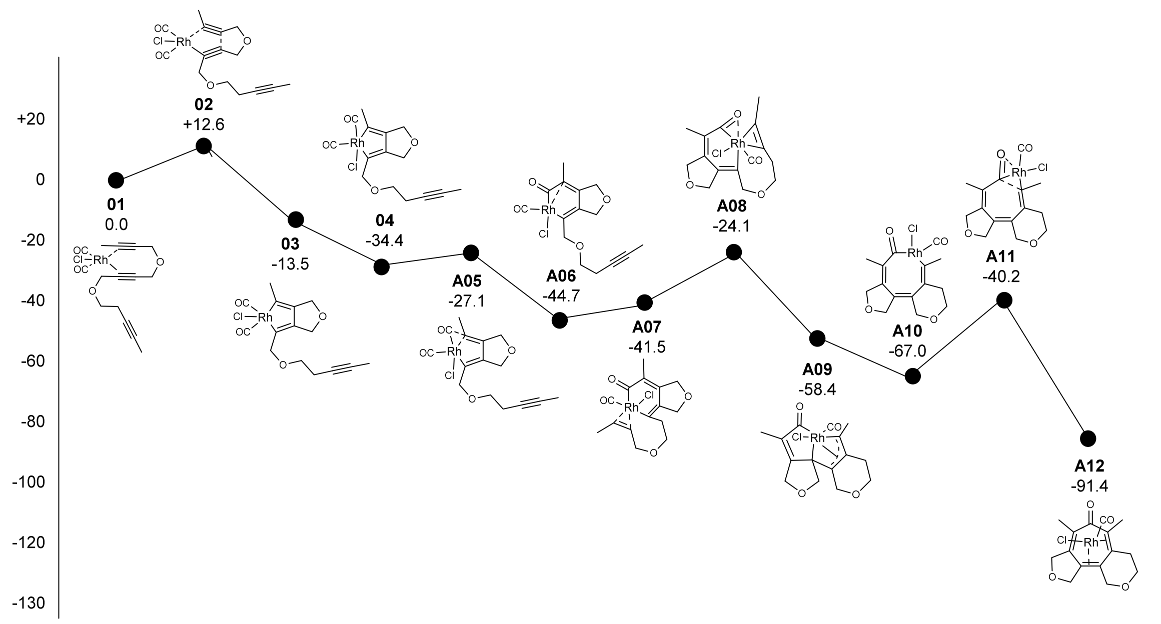

Figure S1. ***Path A***: Rhodacyclohexadienone 2CO Route

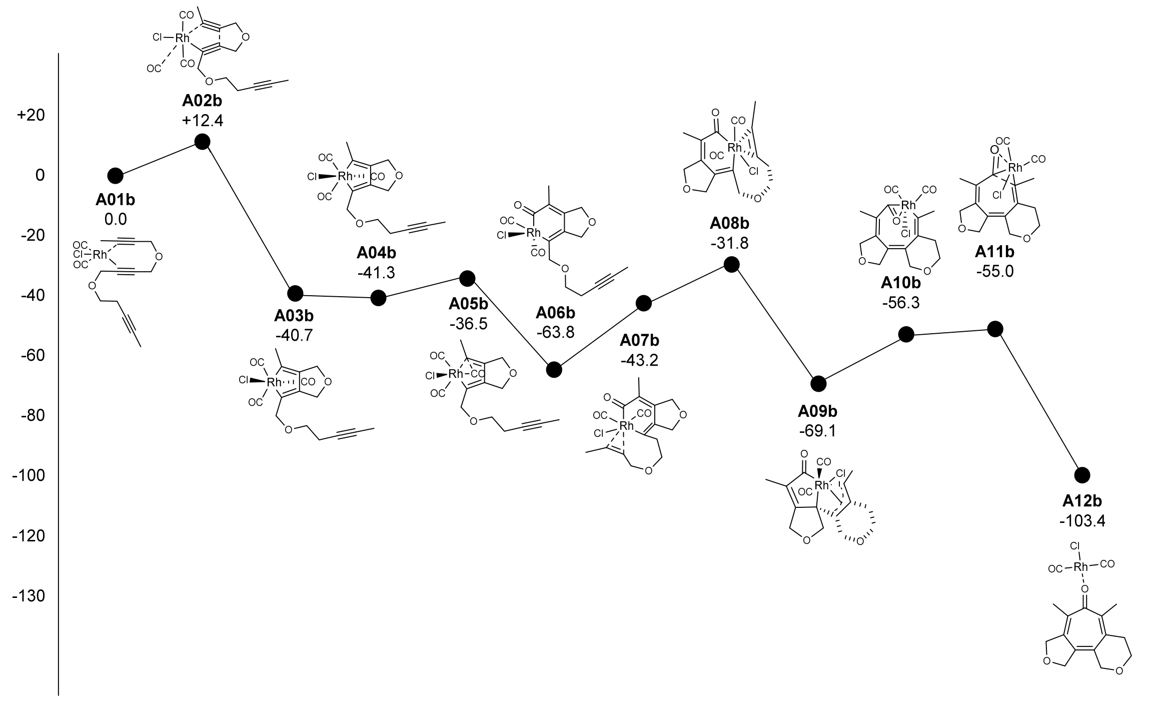

Figure S2. ***Path A***: Rhodacyclohexadienone 3CO Route

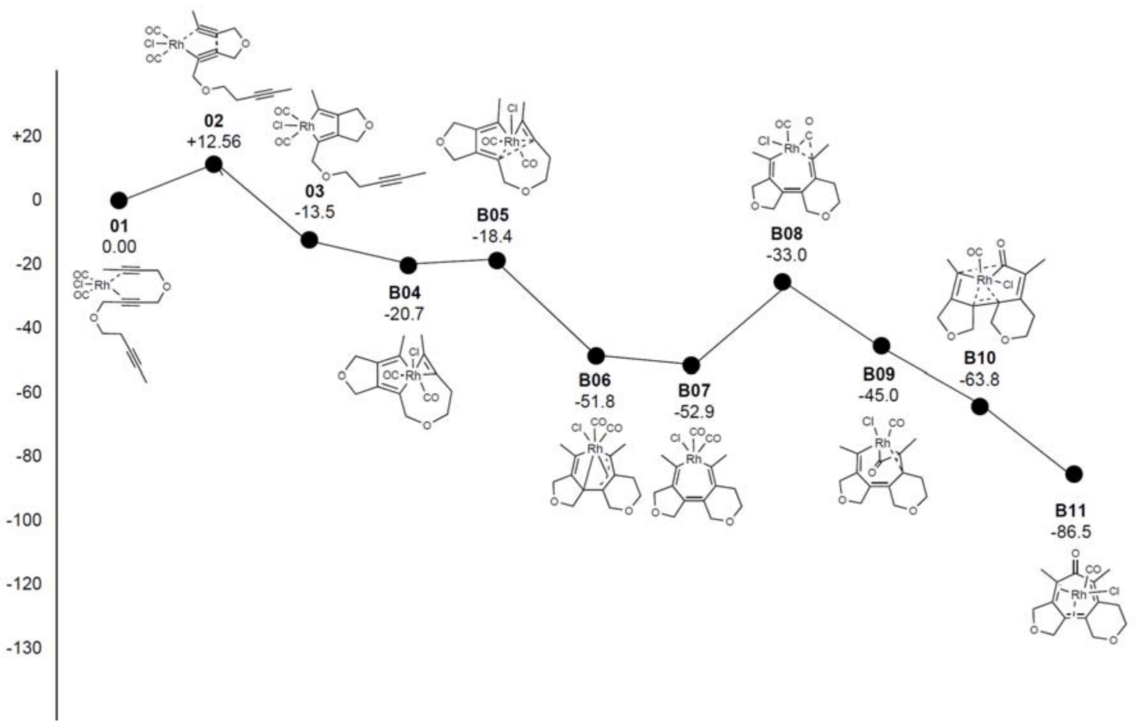

Figure S3. ***Path B***: Rhodacycloheptatriene Route 1

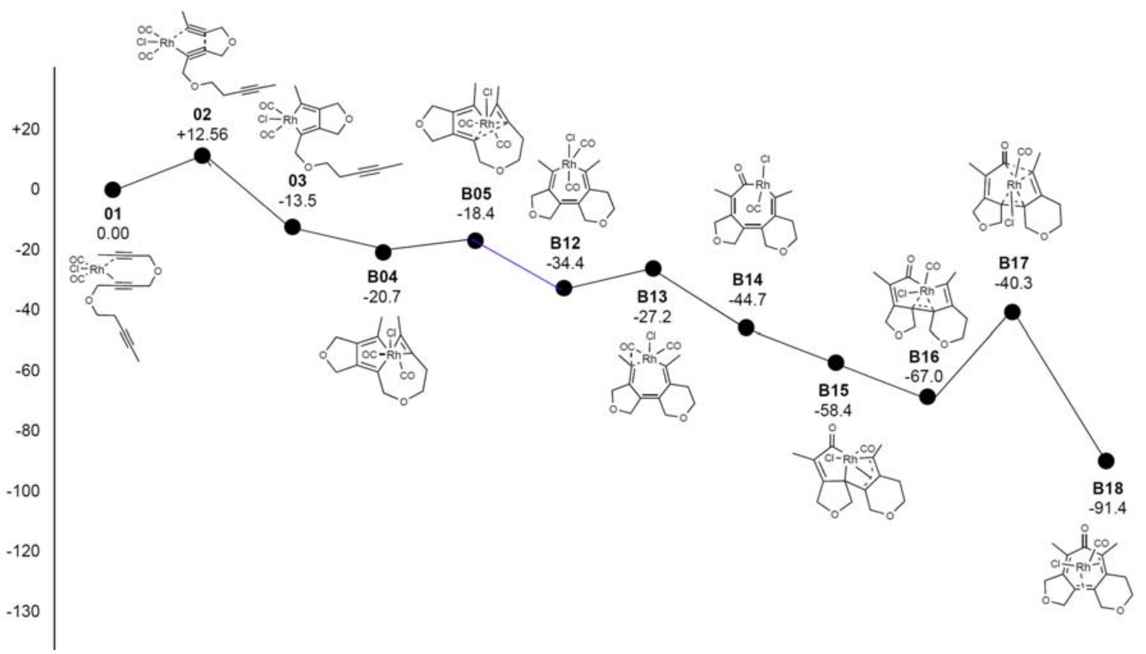

Figure S4. ***Path B***: Rhodacycloheptatriene Route 2

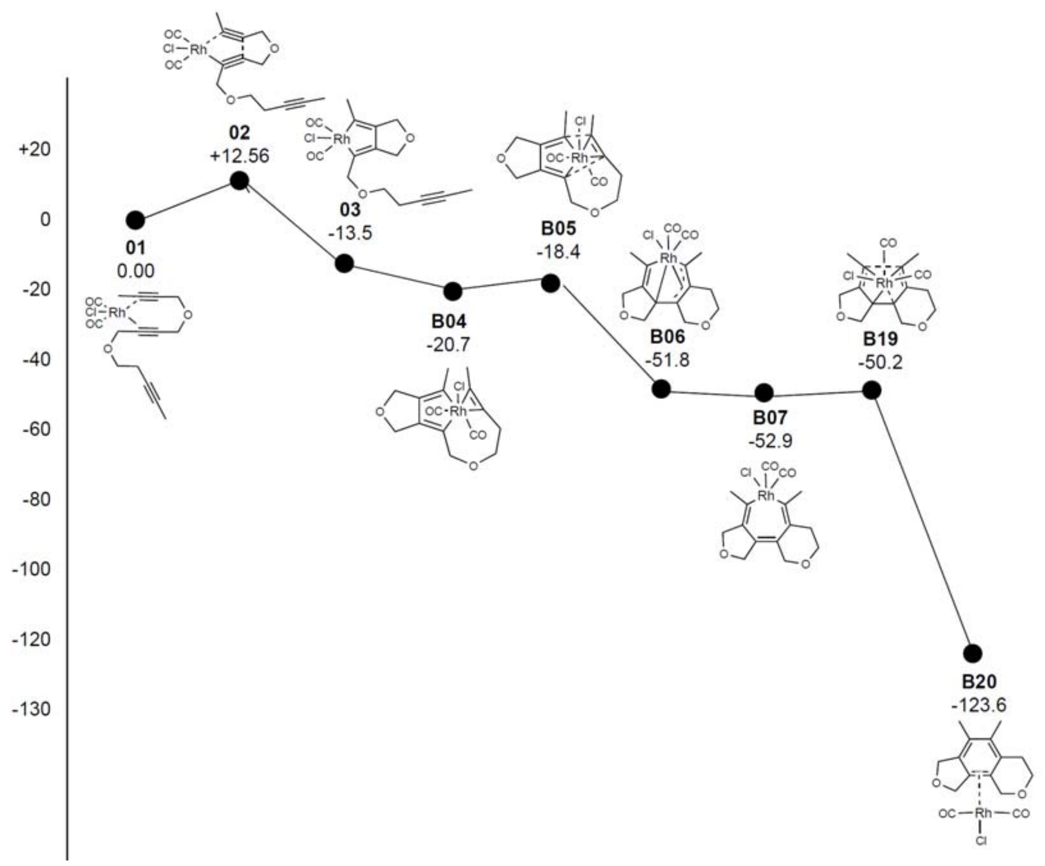

Figure S5. ***Path B***: Route 3, Rhodacycloheptatriene-Aromatization Route

5,5,10,10-Tetra(carbethoxy)tetradeca-2,7,12-triyne (1b)

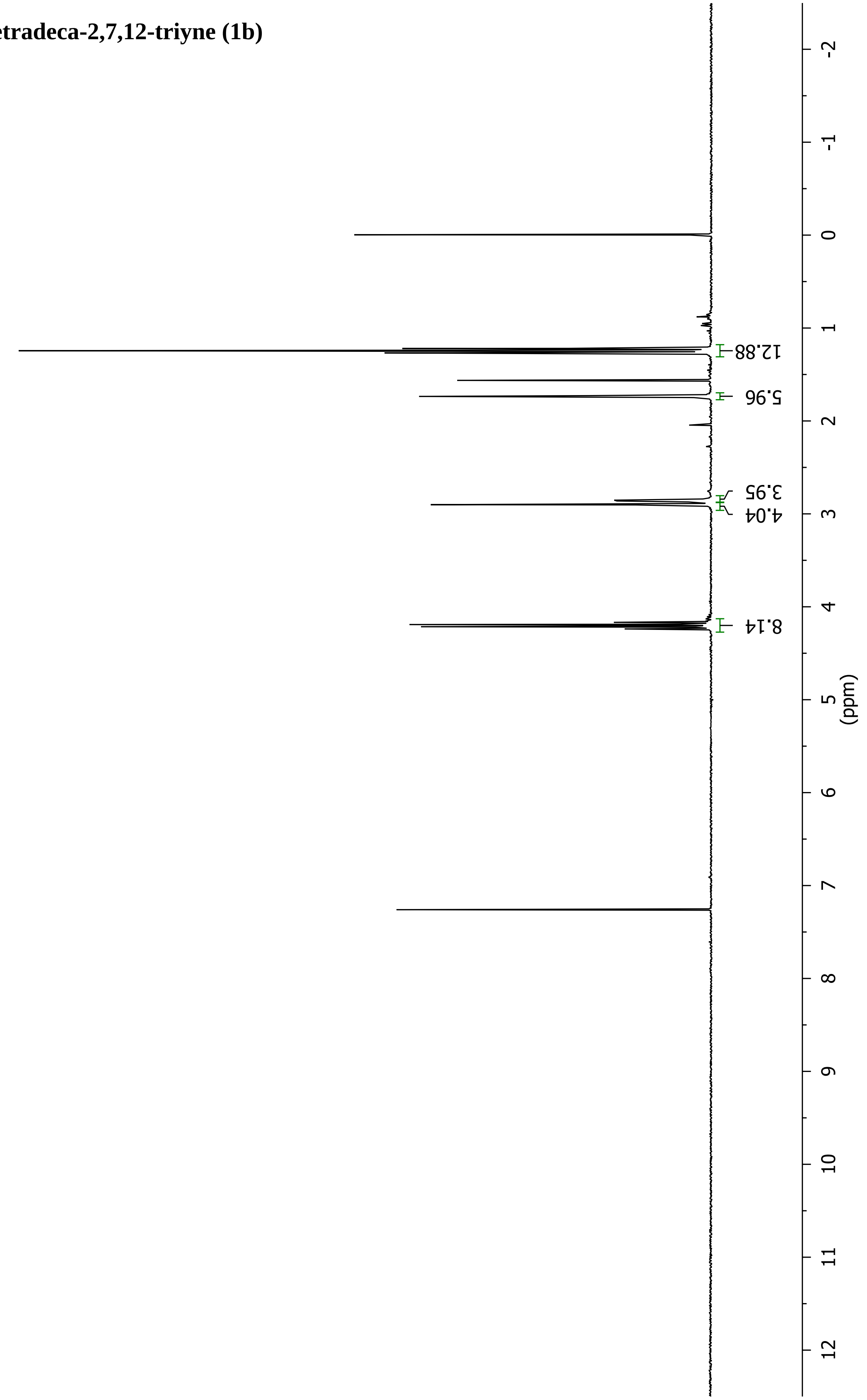

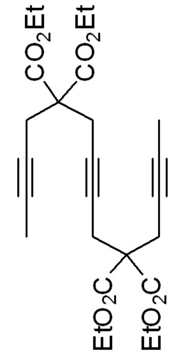

5,5,10,10-Tetra(carbethoxy)tetradeca-2,7,12-triyne (1b)

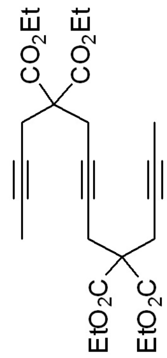

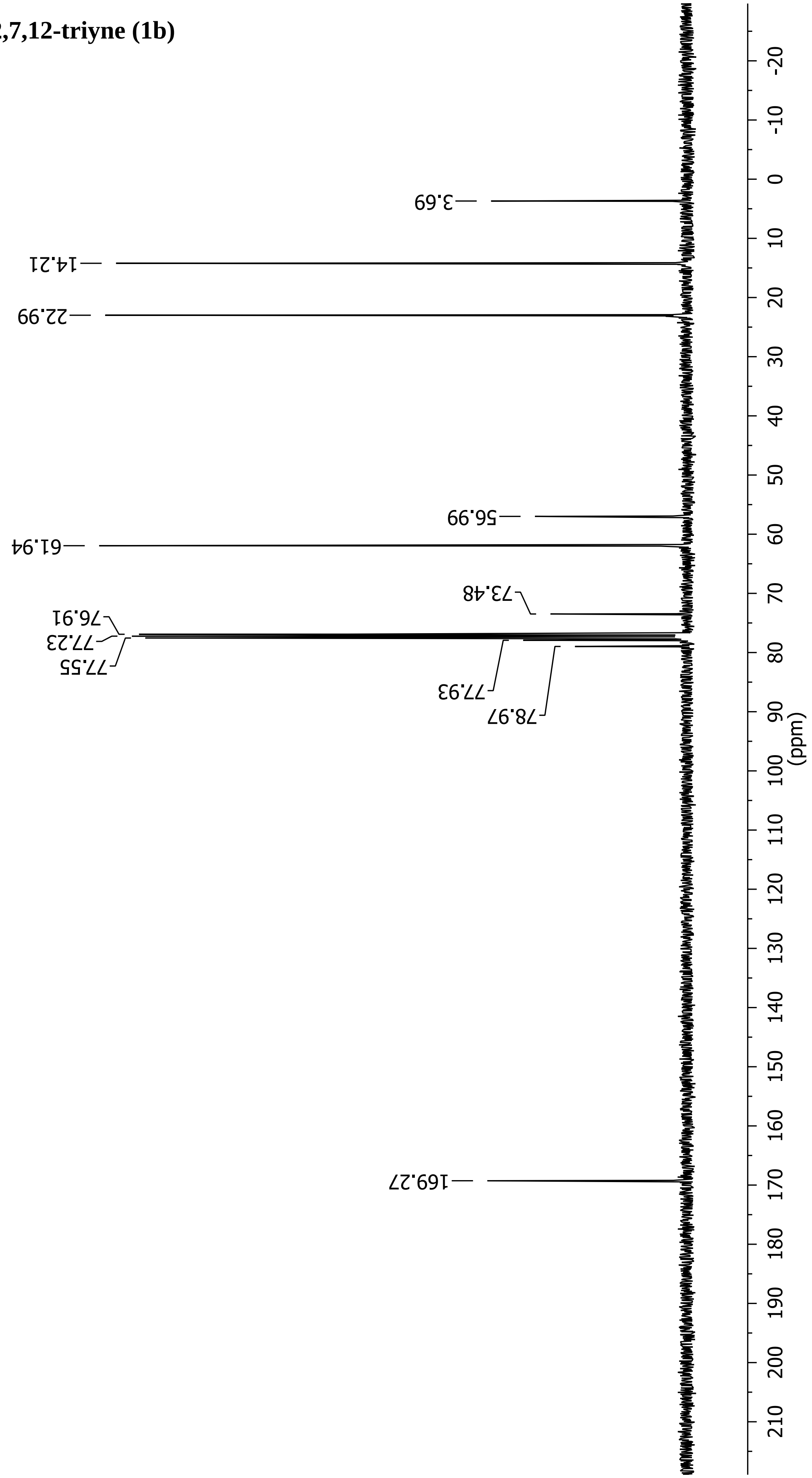

5-(4-Methylbenzenesulfonyl)-10,10-di(carbethoxy)-5-azatetradeca-2,7,12-triyne (1d)

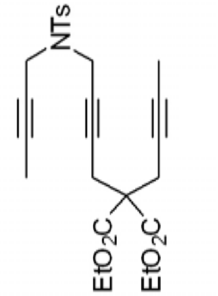

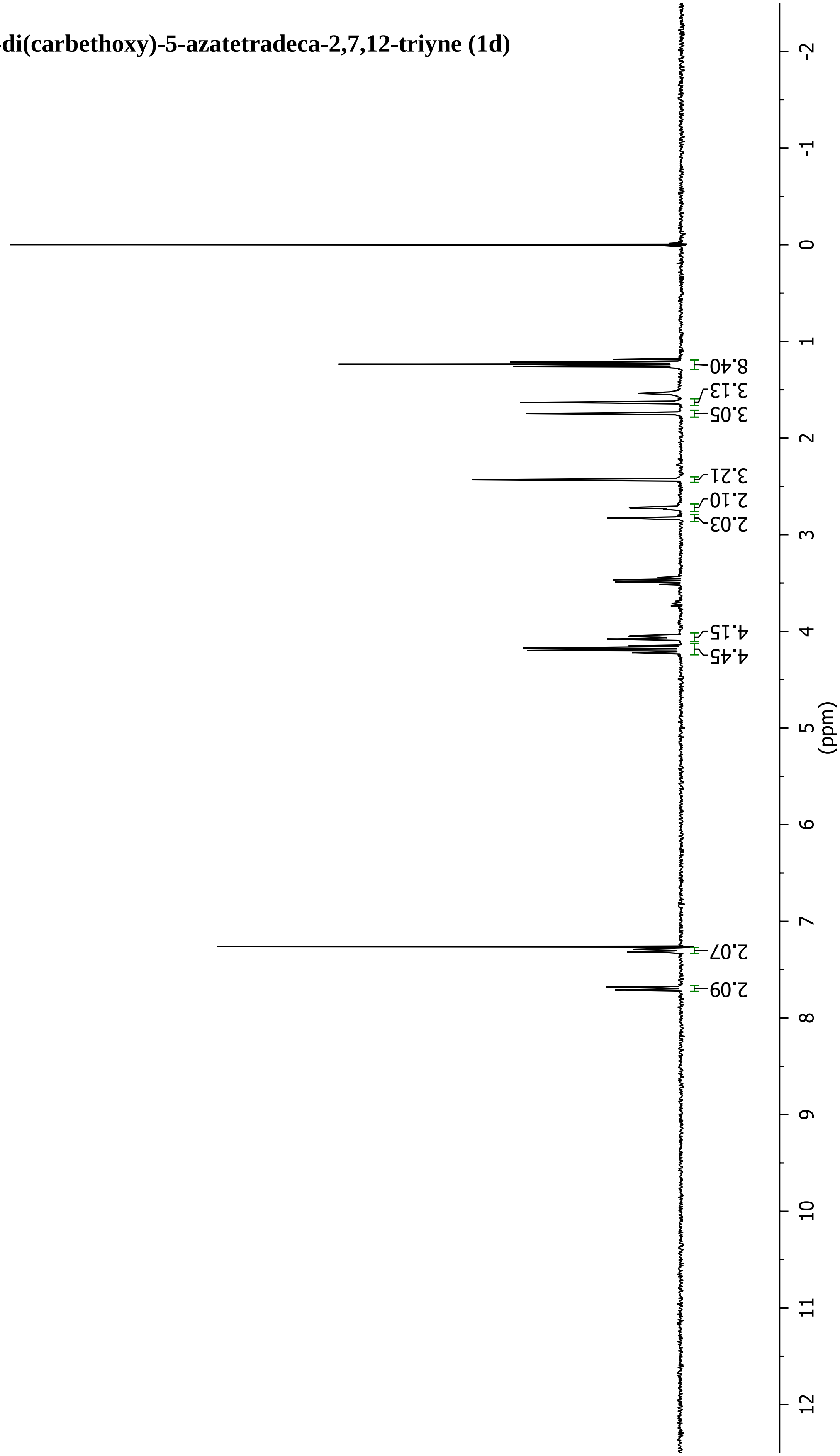

5-(4-Methylbenzenesulfonyl)-10,10-di(carbethoxy)-5-azatetradeca-2,7,12-triyne (1d)

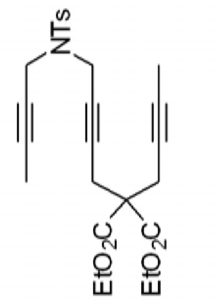

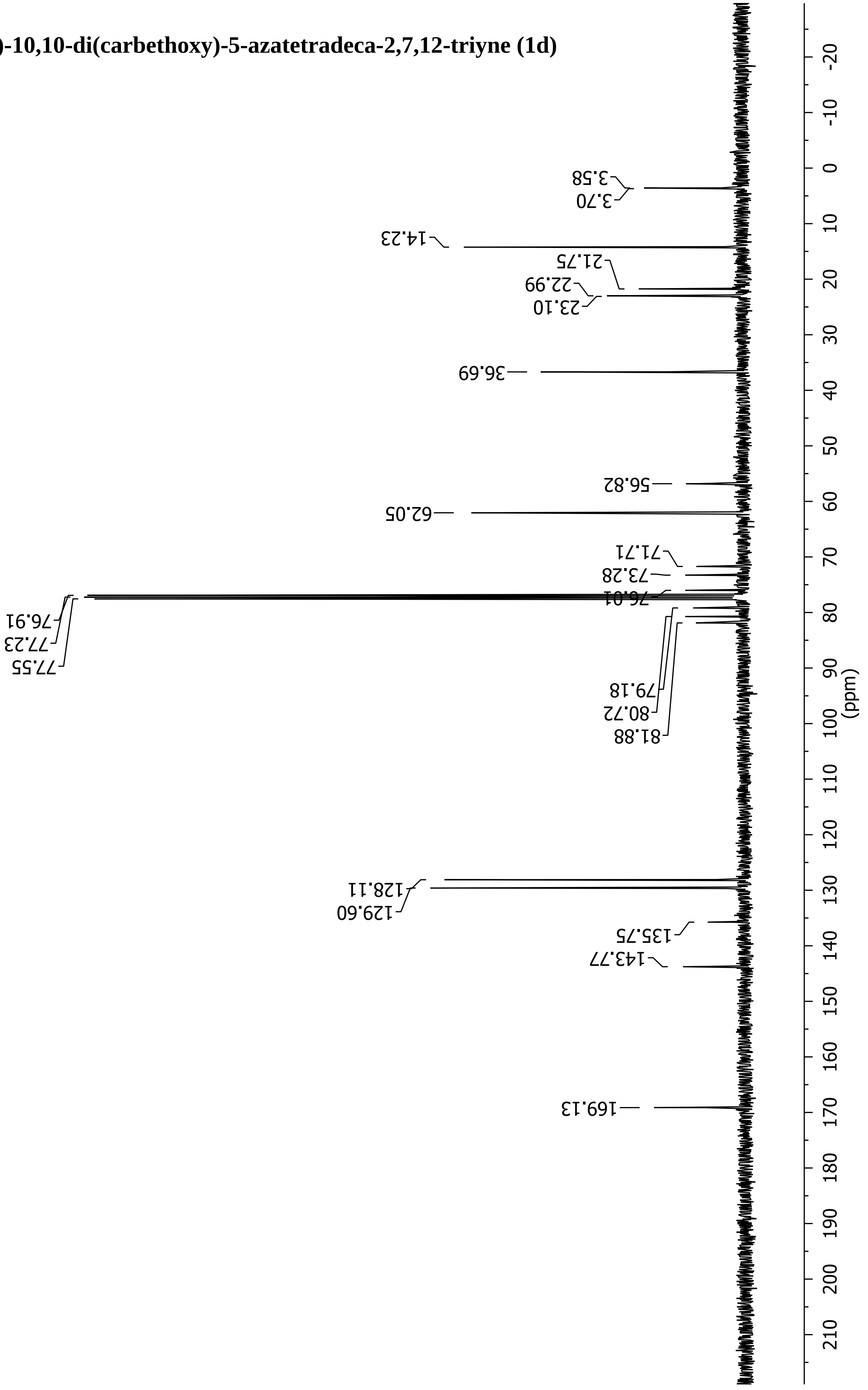

5,5,10,10-Tetra(carbethoxy)pentadeca-2,7,13-triyne (1e)

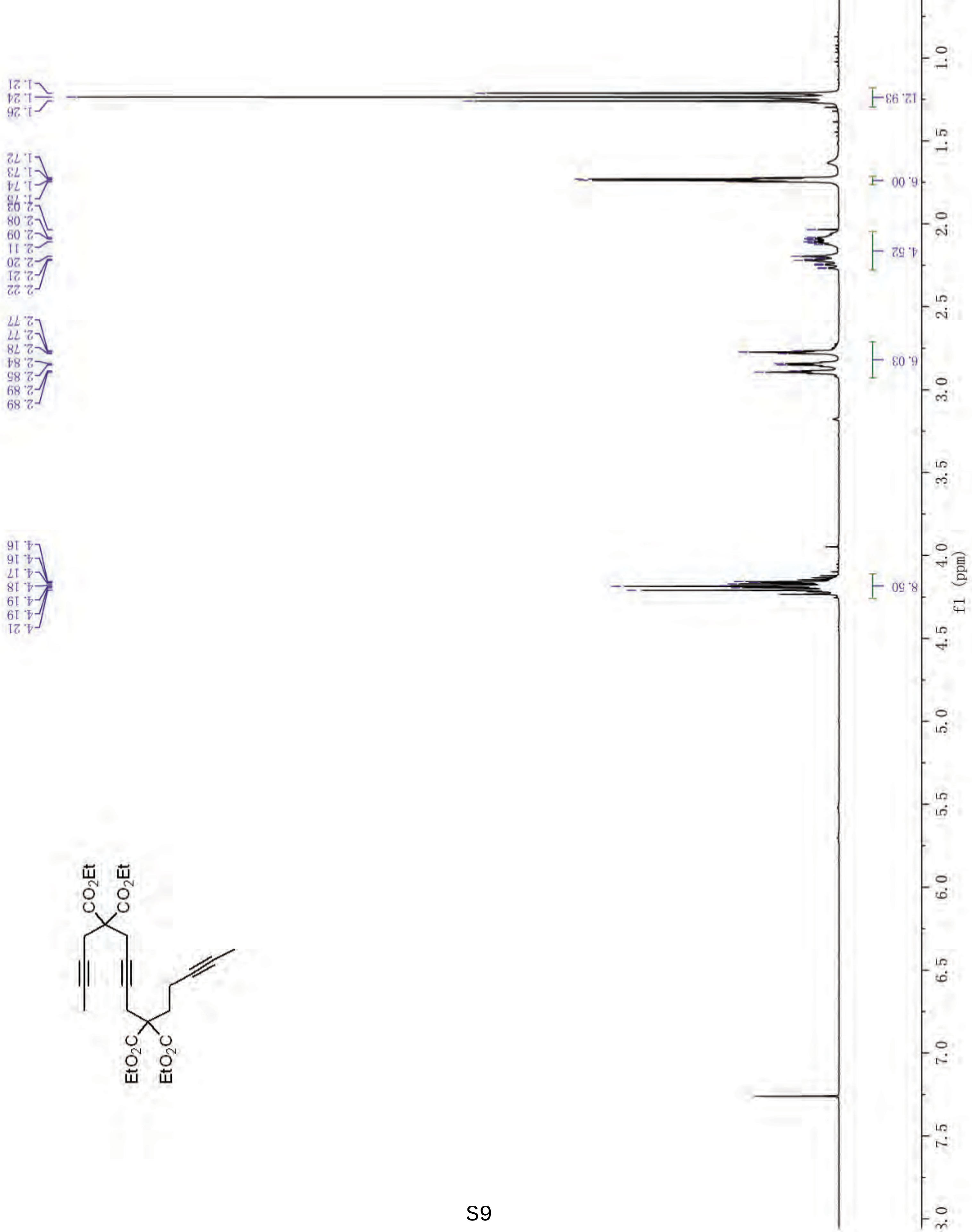

5,5,10,10-Tetra(carbethoxy)pentadeca-2,7,13-triyne (1e)

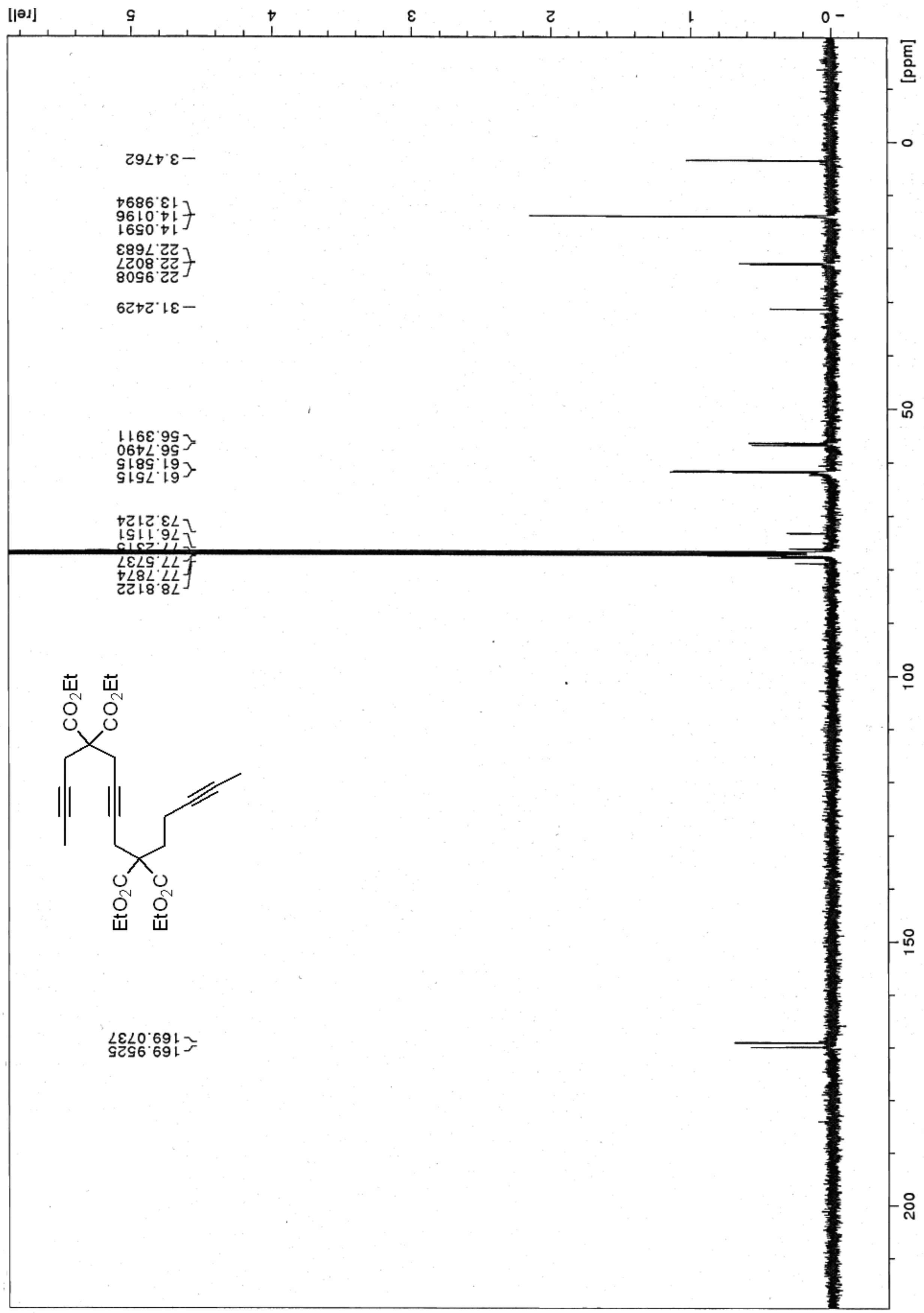

1-Phenyl-4,4,9,9-tetra(carbethoxy)tetradeca-1,6,12-triyne (1f)

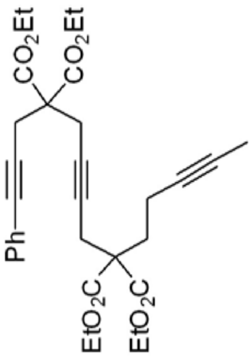

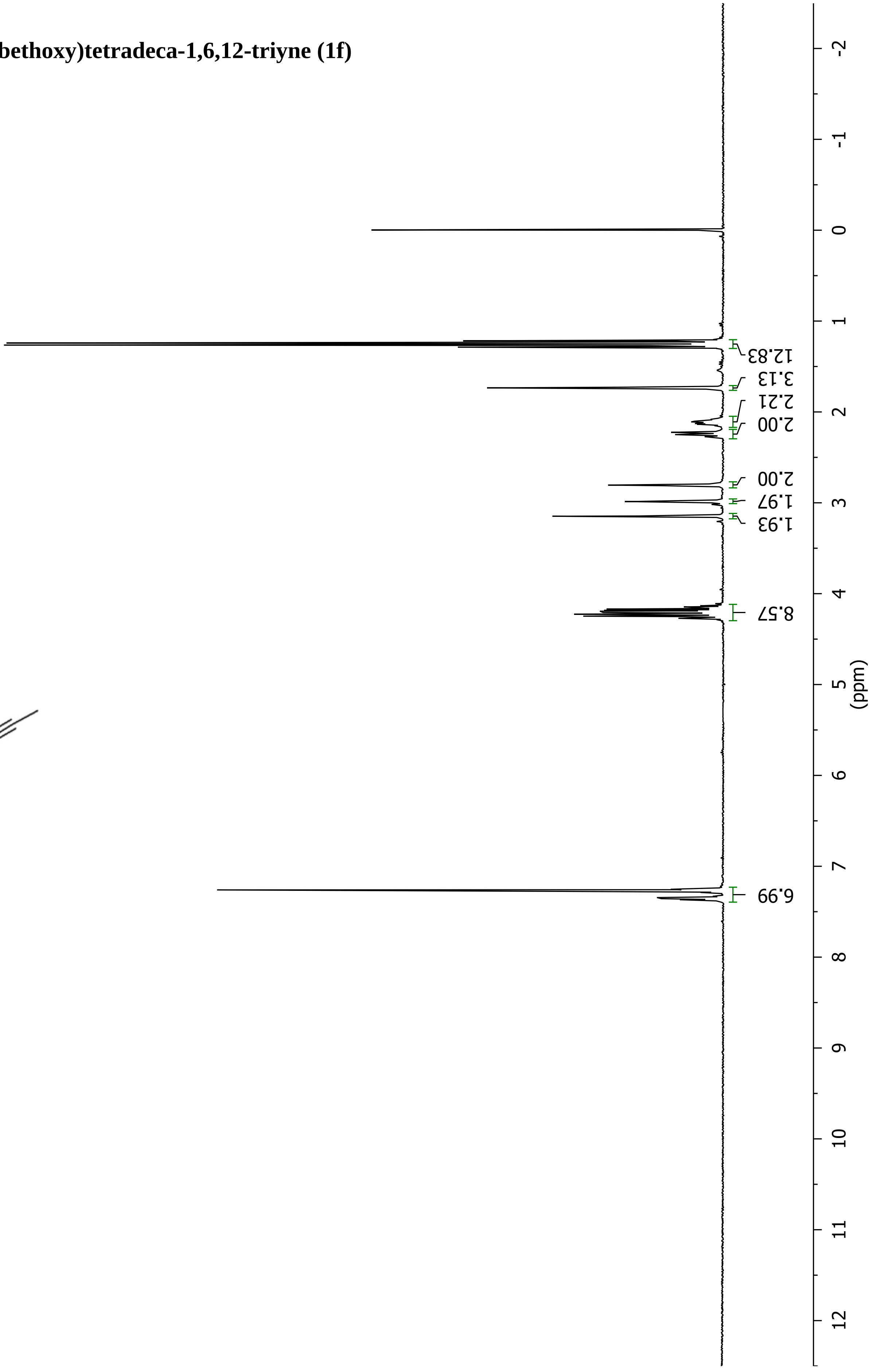

1-Phenyl-4,4,9,9-tetra(carbethoxy)tetradeca-1,6,12-triyne (1f)

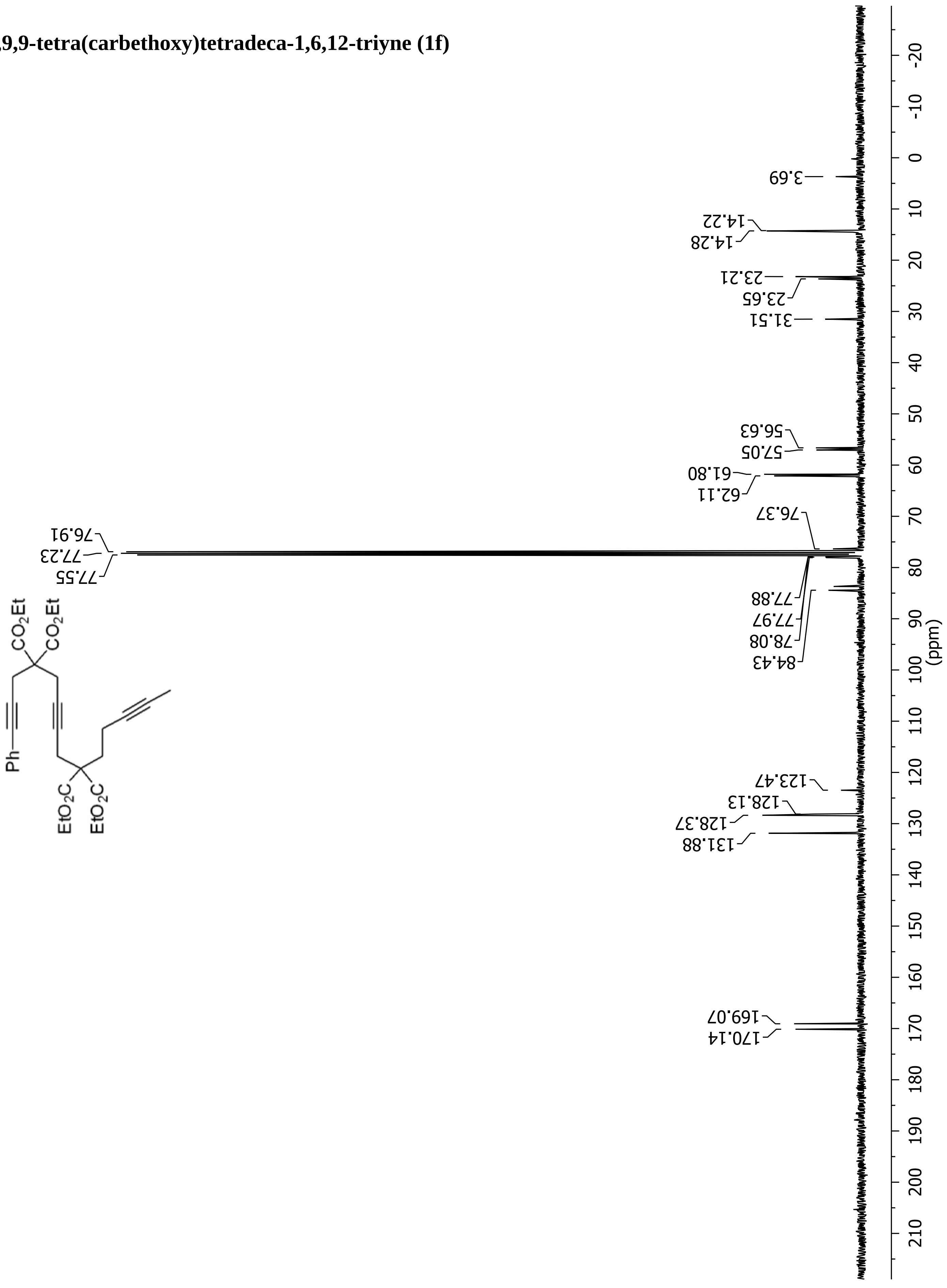

1-Phenyl-9-(4-methylbenzenesulfonyl)-4,4-di(carbethoxy)-9-azatetradeca-1,6,12-triyne (1g)

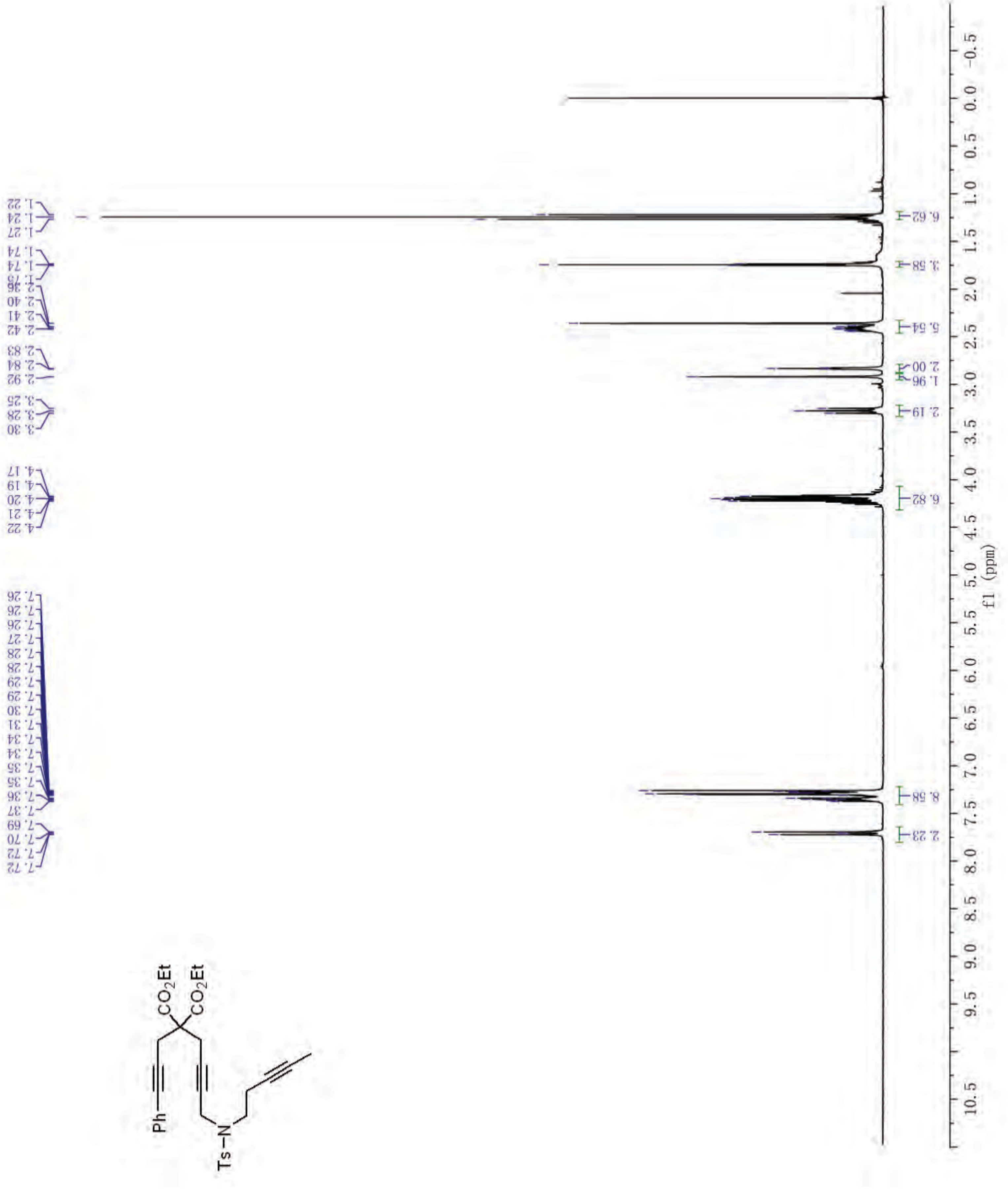

1-Phenyl-9-(4-methylbenzenesulfonyl)-4,4-di(carbethoxy)-9-azatetradeca-1,6,12-triyne (1g)

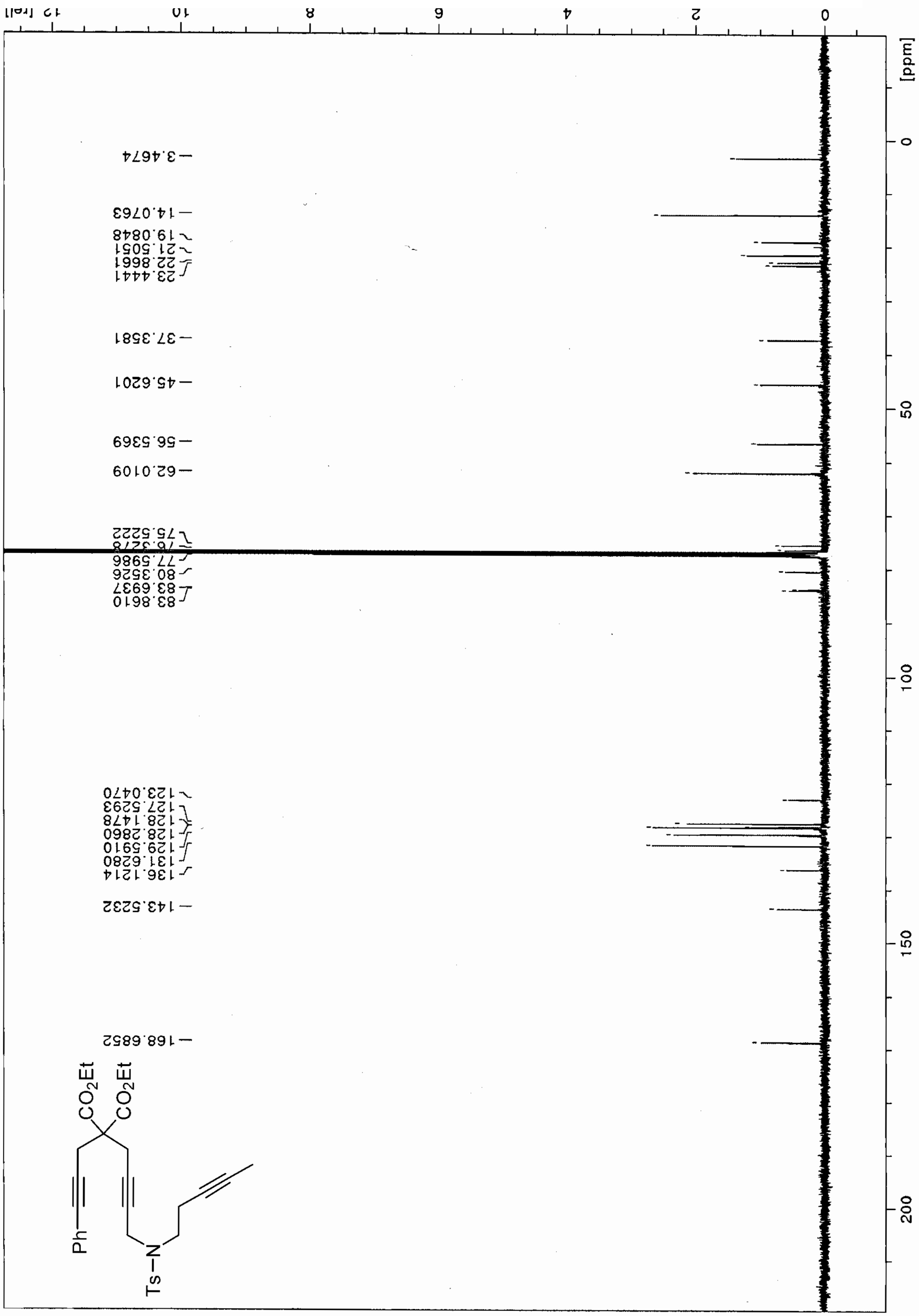

7-(4-Methylbenzenesulfonyl)-12,12-di(carbethoxy)-7-azahexadeca-2,9,14-triyne (1h)

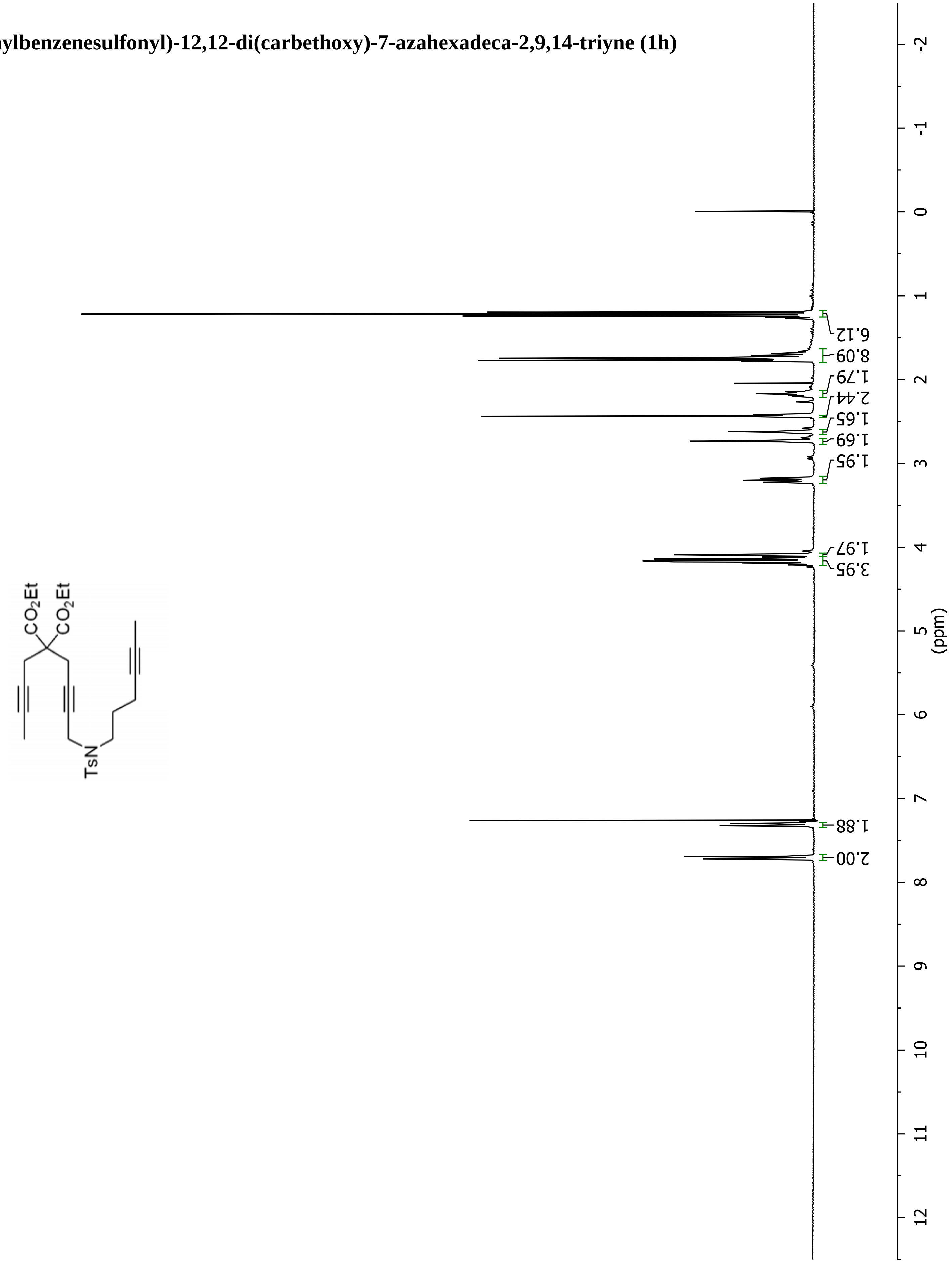

7-(4-Methylbenzenesulfonyl)-12,12-di(carbethoxy)-7-azahexadeca-2,9,14-triyne (1h)

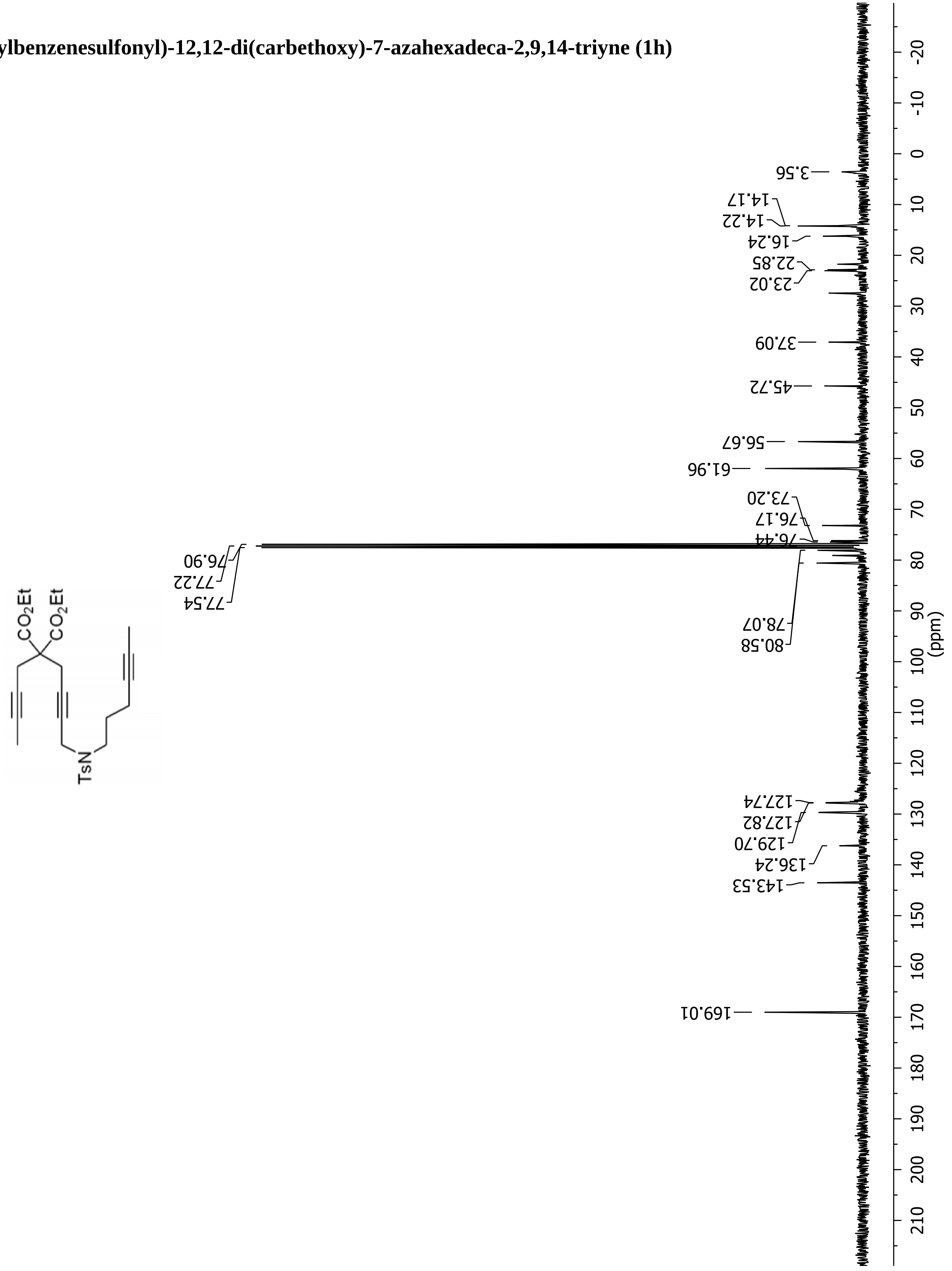

1-Phenyl-9-(4-Methylbenzenesulfonyl)-4,4-di(carbethoxy)-9-azapentadeca-1,6,13-triyne (1i)

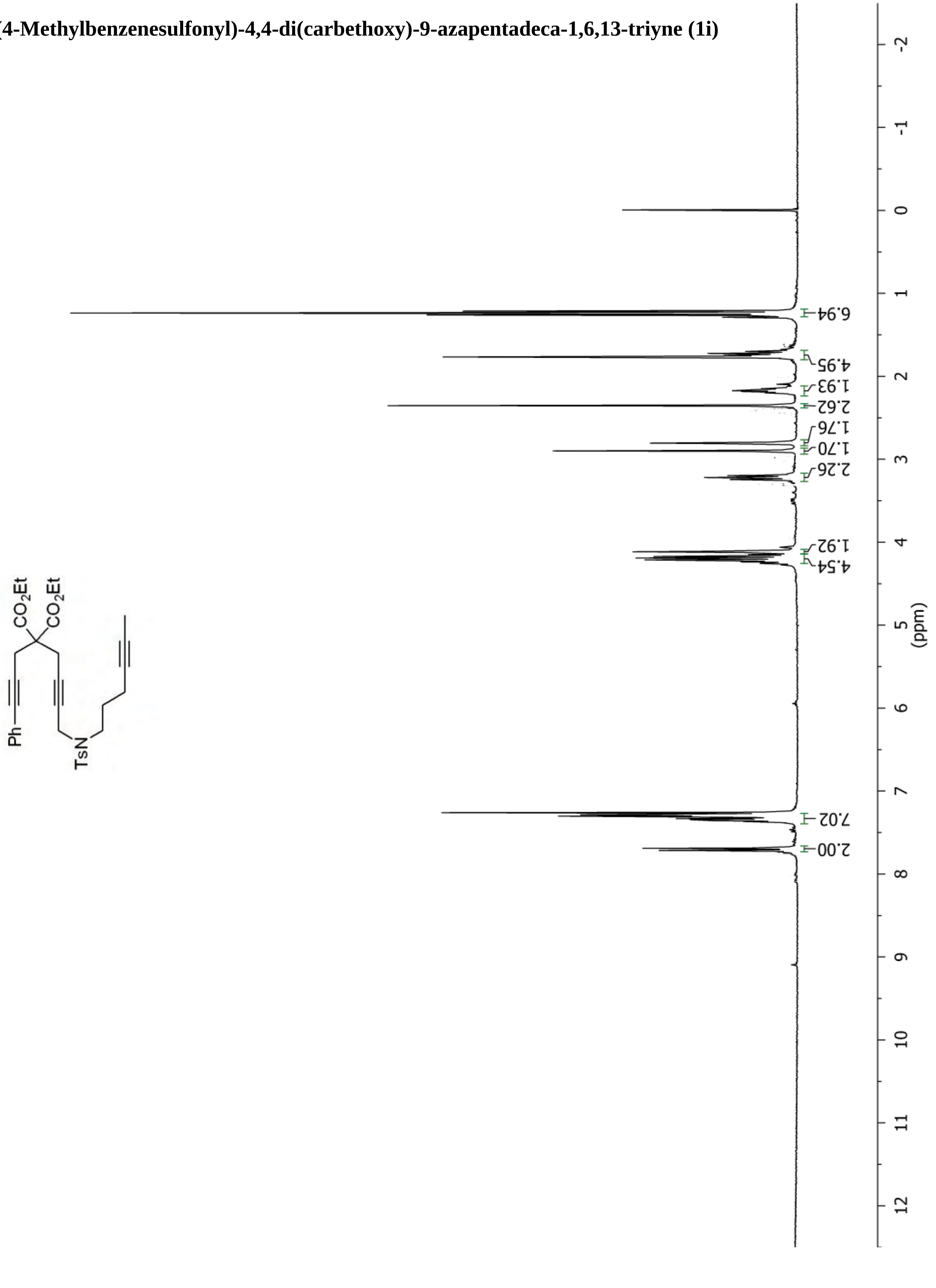

1-Phenyl-9-(4-Methylbenzenesulfonyl)-4,4-di(carbethoxy)-9-azapentadeca-1,6,13-triyne (1i)

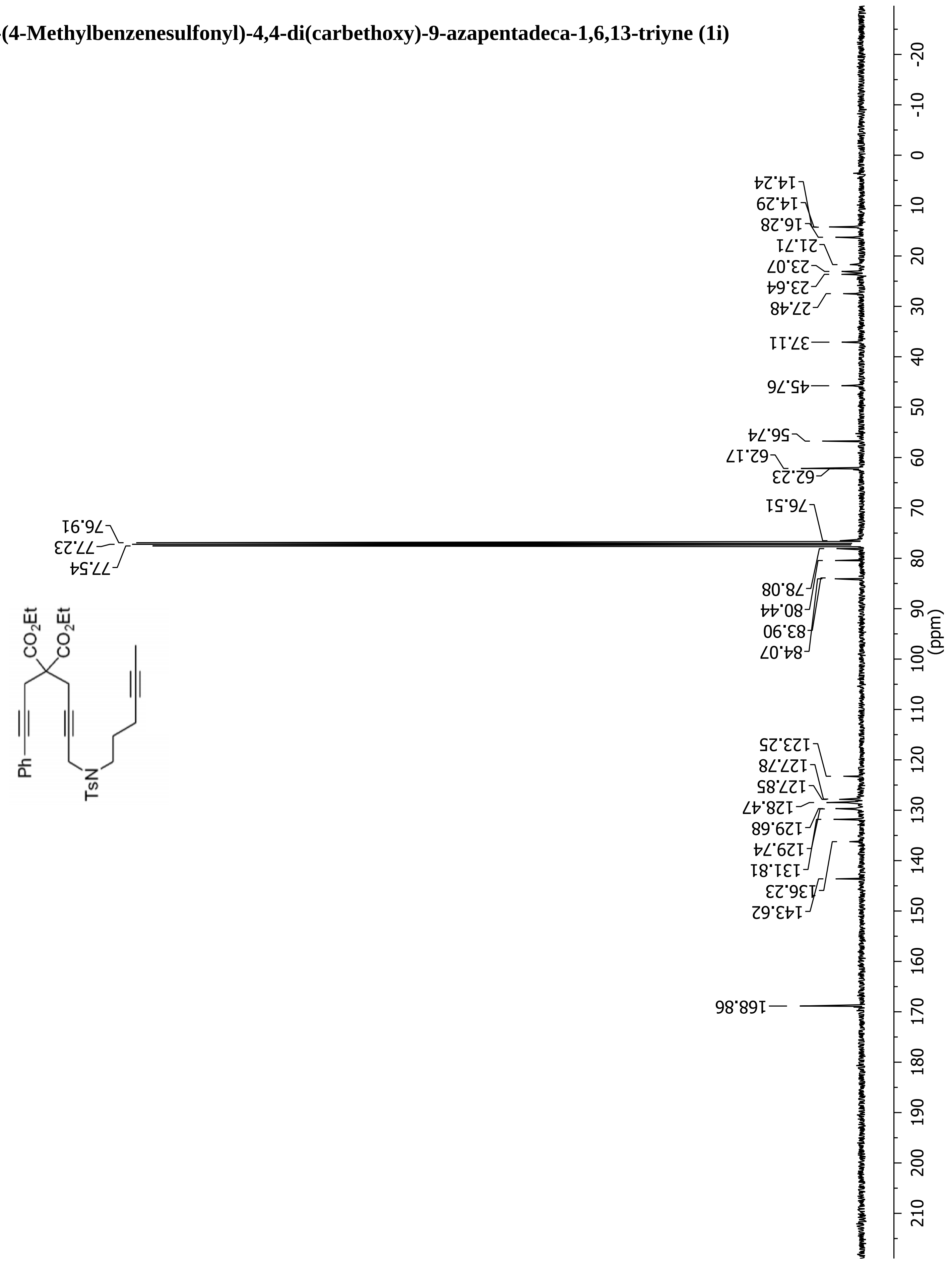

4,6-Dimethyl-2,2,8,8,tetra(carbethoxy)-1,3,7,8-tetrahydro-5-oxo-cyclopenta[*e*]azulene (2b)

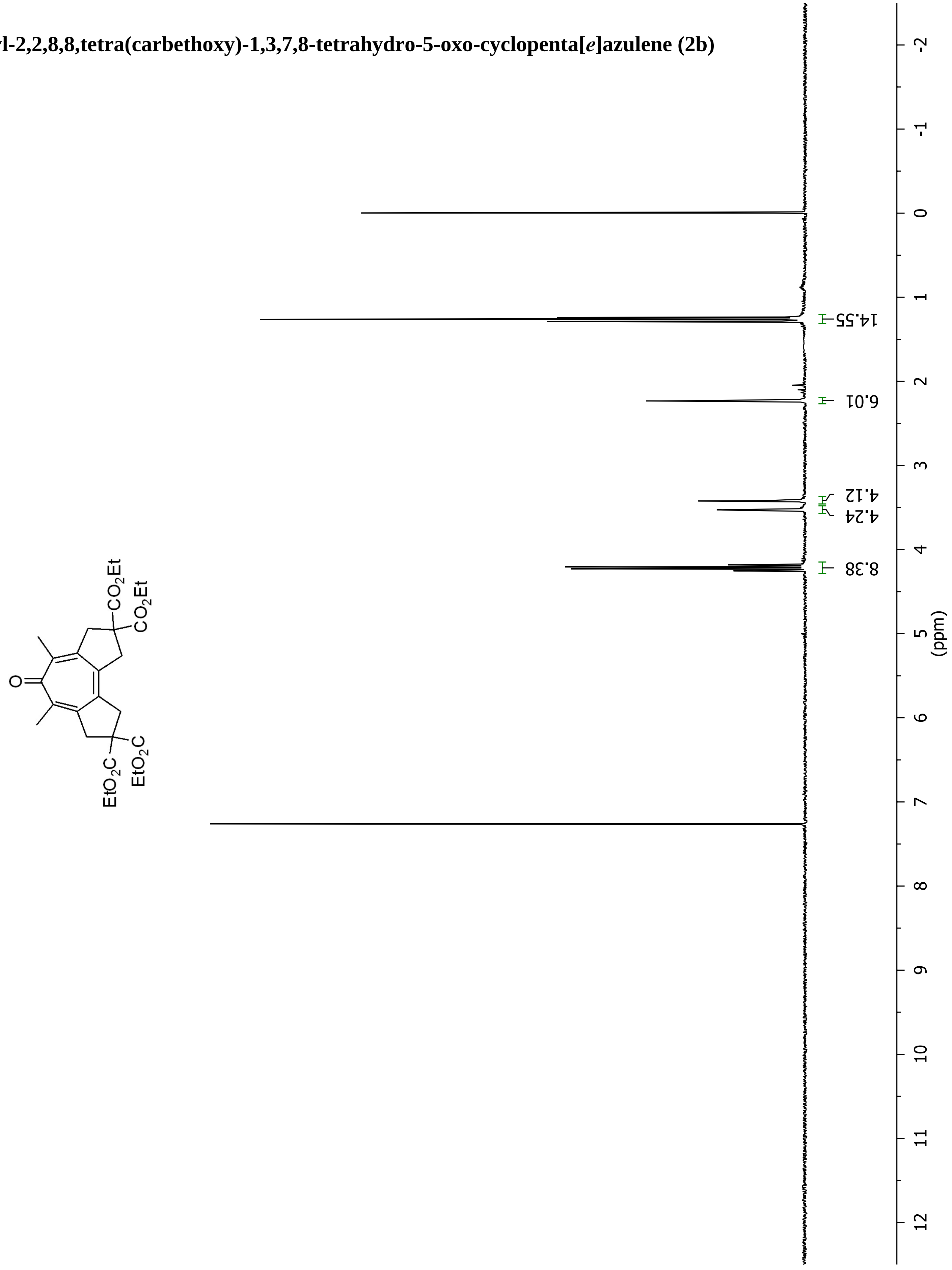

4,6-Dimethyl-2,2,8,8,tetra(carbethoxy)-1,3,7,8-tetrahydro-5-oxo-cyclopenta[*e*]azulene (2b)

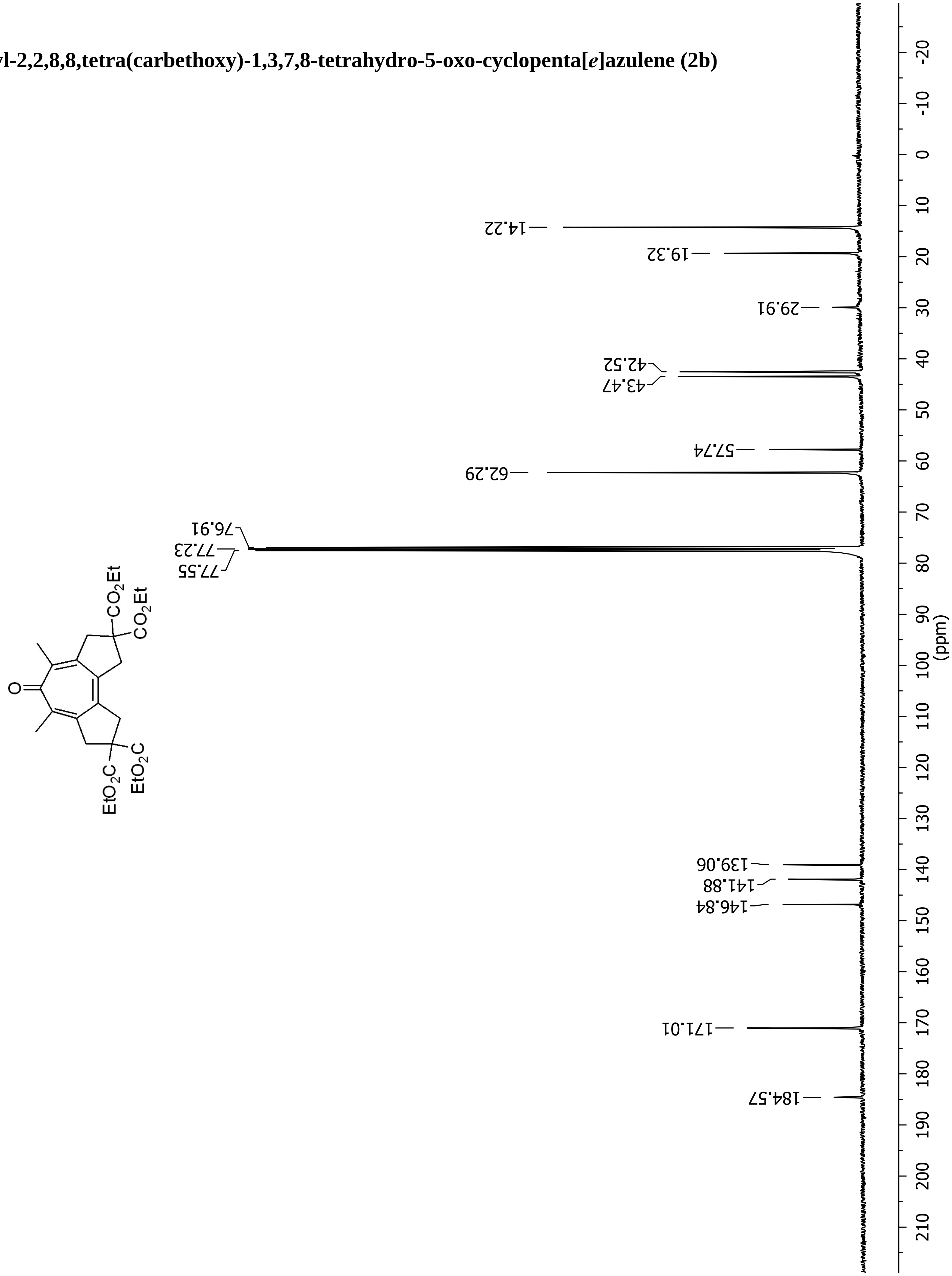

4,6-Dimethyl-1,3,7,8-tetrahydro-5-oxo-2,8-dioxa-cyclopenta[*e*]azulene (2c)

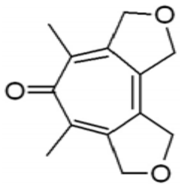

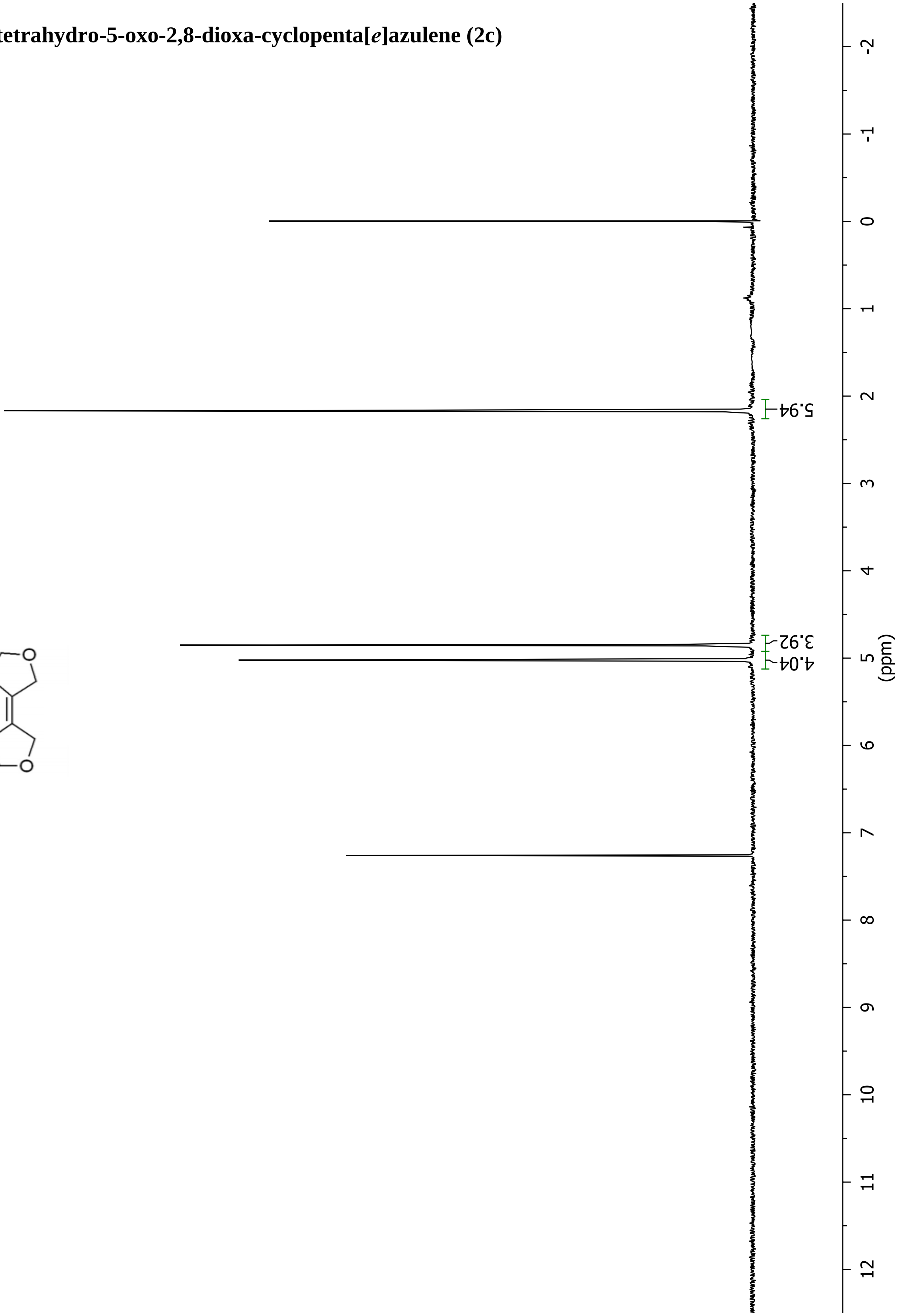

4,6-Dimethyl-1,3,7,8-tetrahydro-5-oxo-2,8-dioxa-cyclopenta[*e*]azulene (2c)

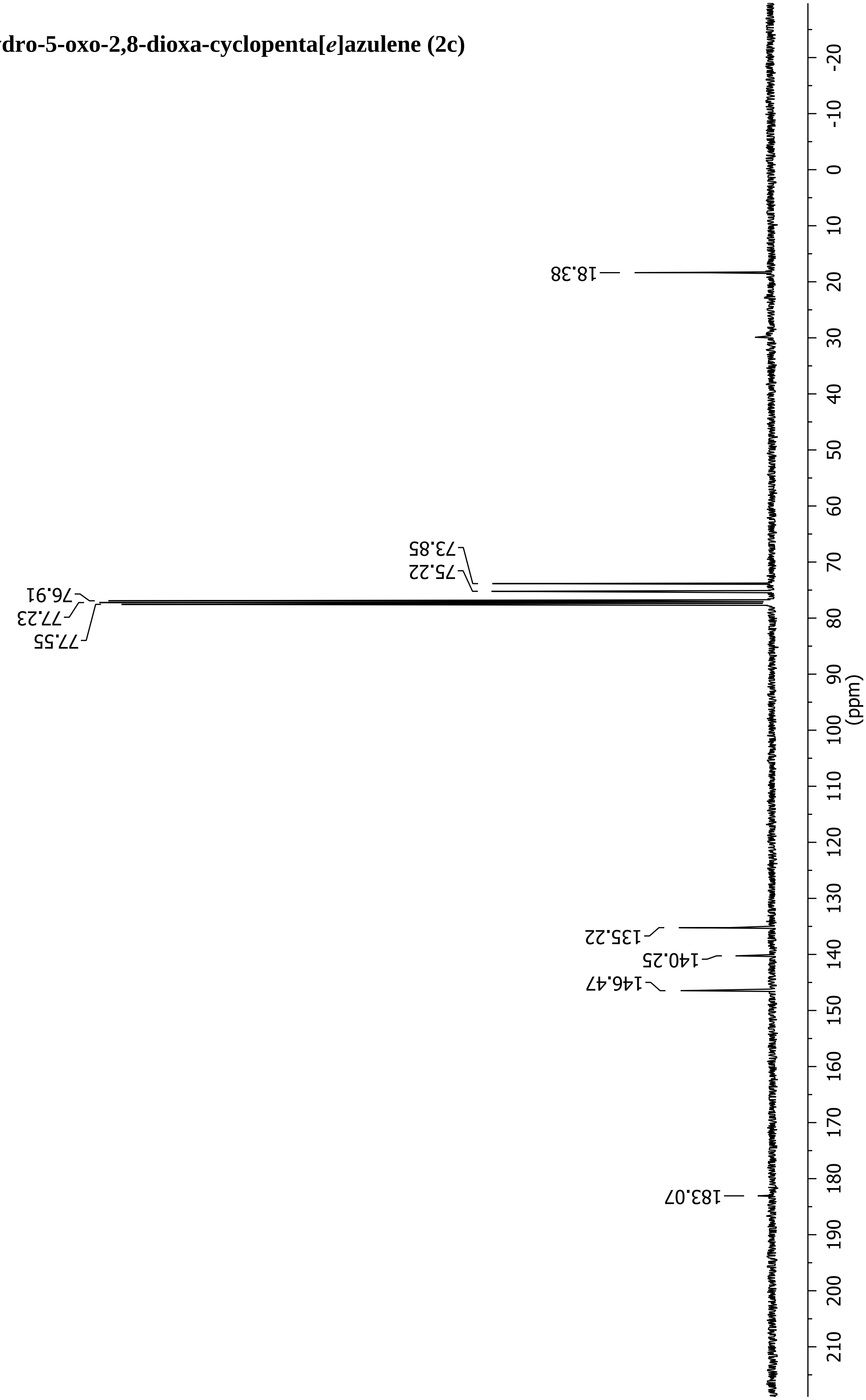

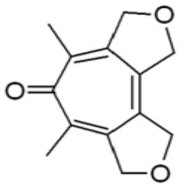

4,6-Dimethyl-8,8-di(carbethoxy)-1,3,7,8-tetrahydro-2-(4-Methylbenzenesulfonyl)-5-oxo-2-aza-cyclopenta[*e*]azulene (2d)

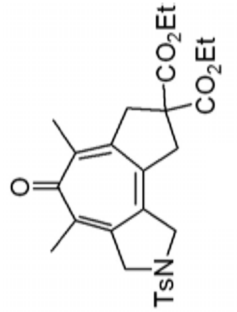

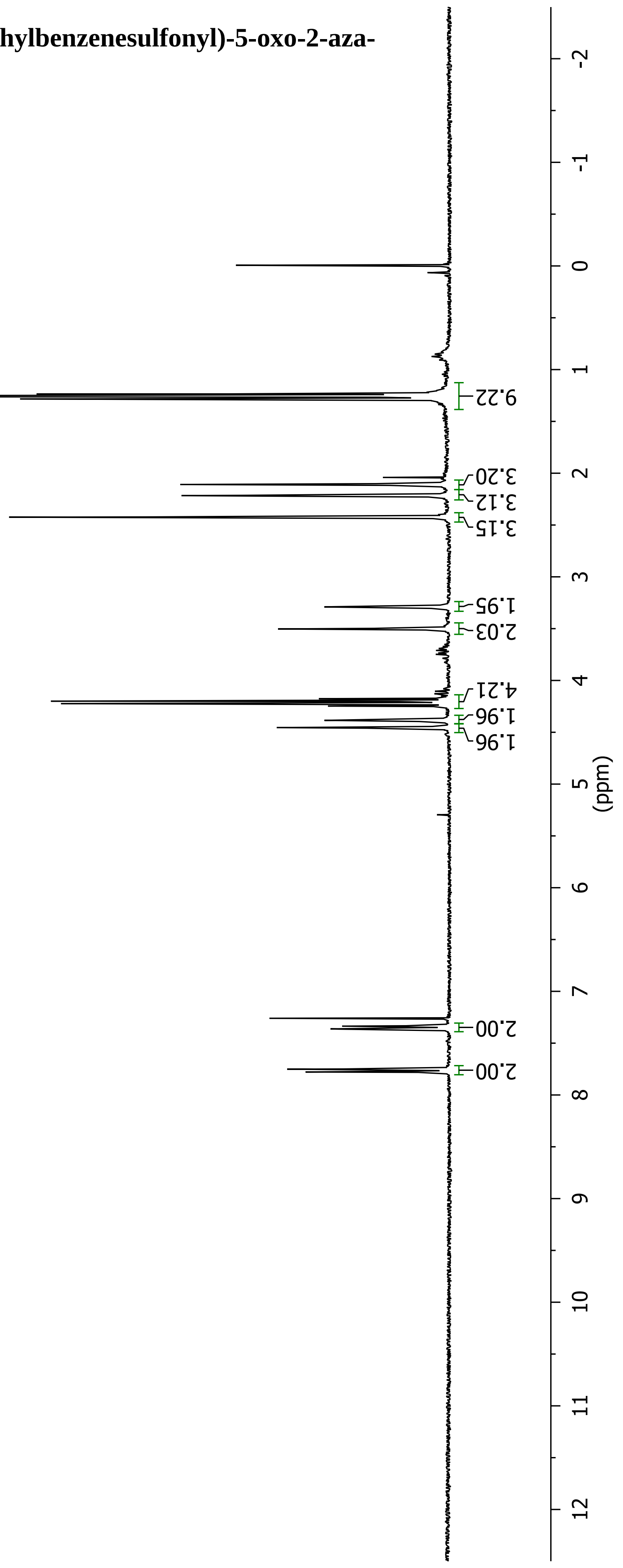

4,6-Dimethyl-8,8-di(carbethoxy)-1,3,7,8-tetrahydro-2-(4-Methylbenzenesulfonyl)-5-oxo-2-aza-cyclopenta[*e*]azulene (2d)

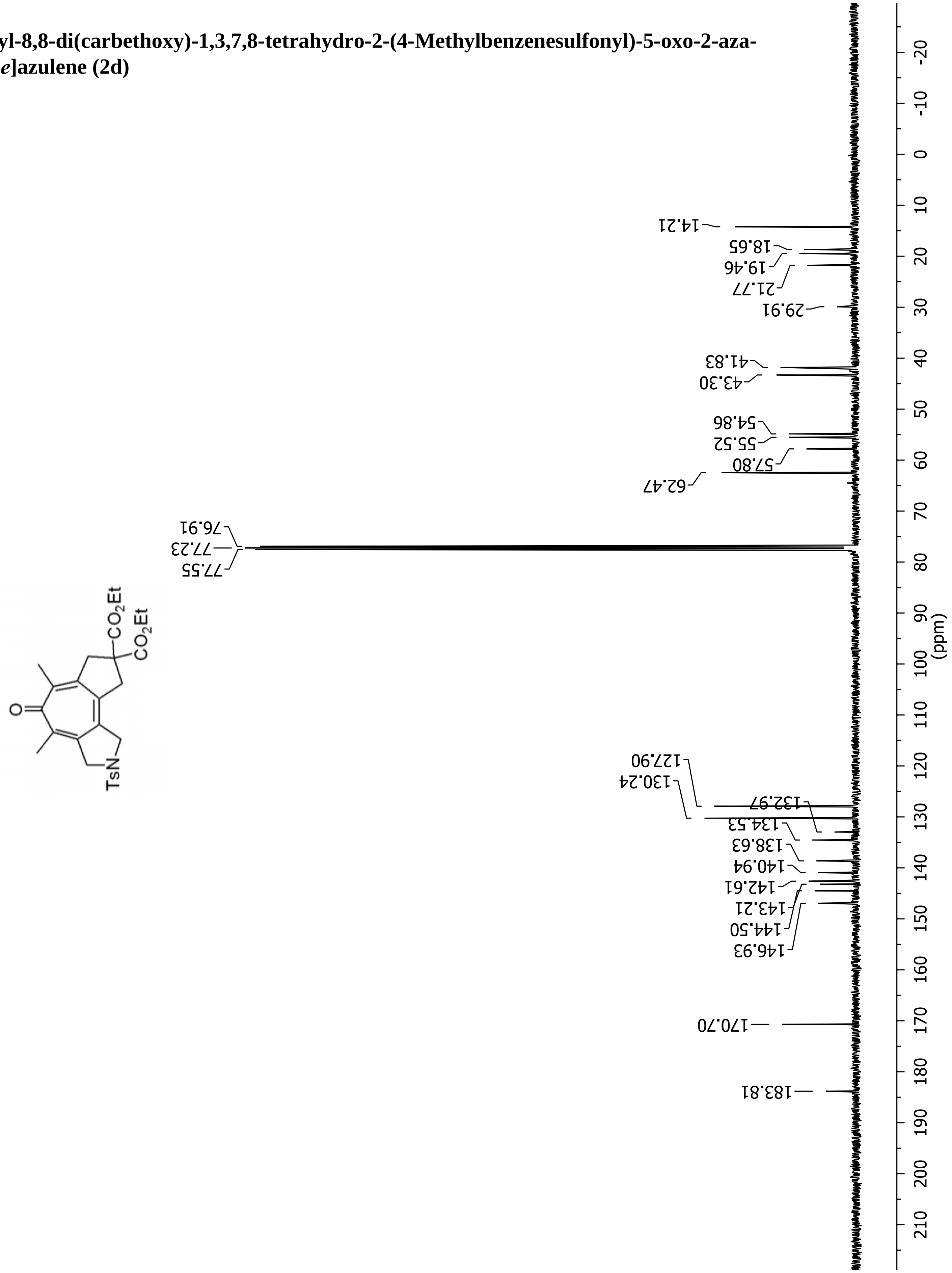

4,6-Dimethyl-2,2,9,9-tetra(carbethoxy)-1,3,7,8,9-pentahydro-5-oxo-benzo[*e*]azulene (2e)

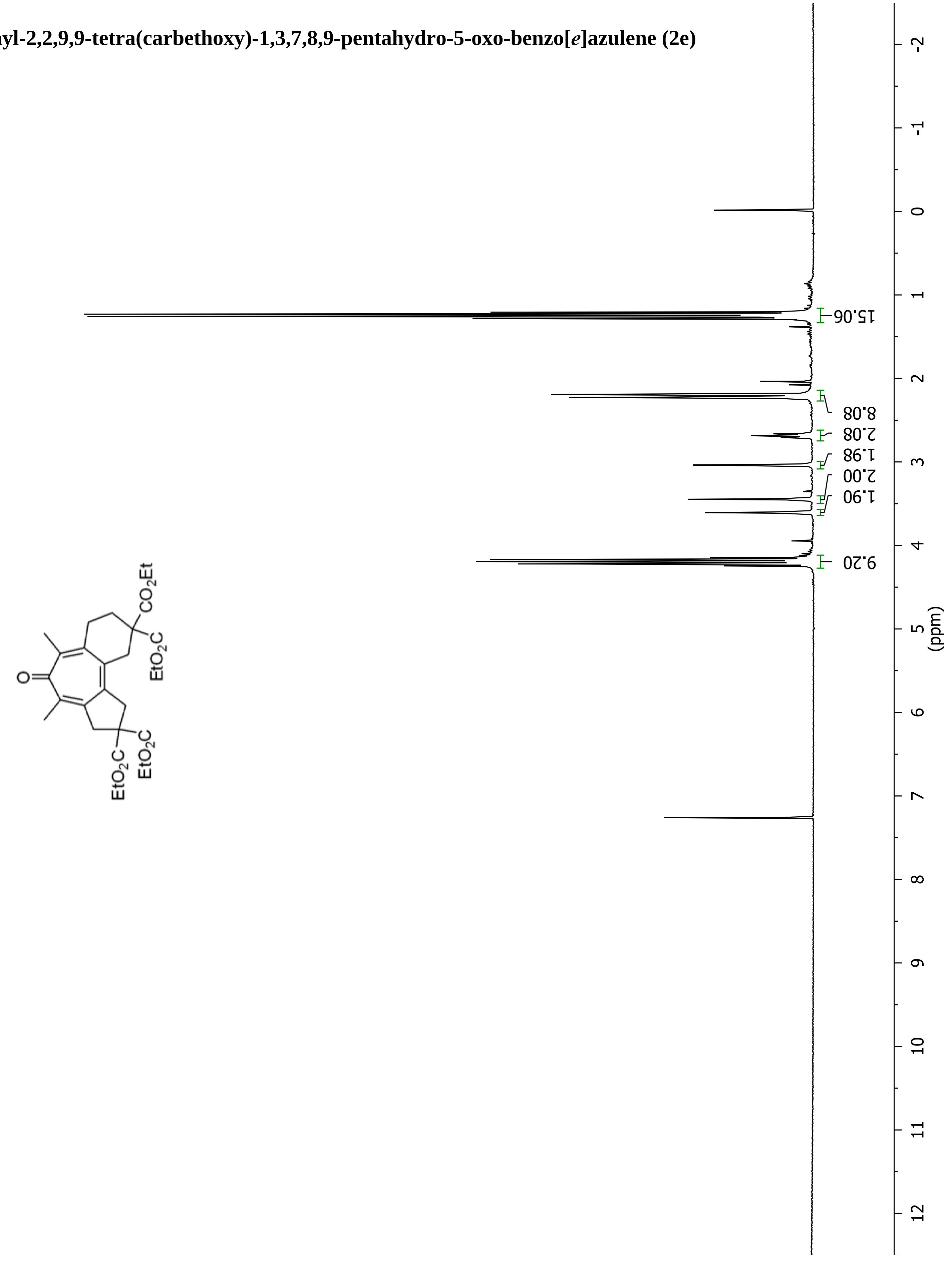

4,6-Dimethyl-2,2,9,9-tetra(carbethoxy)-1,3,7,8,9-pentahydro-5-oxo-benzo[*e*]azulene (2e)

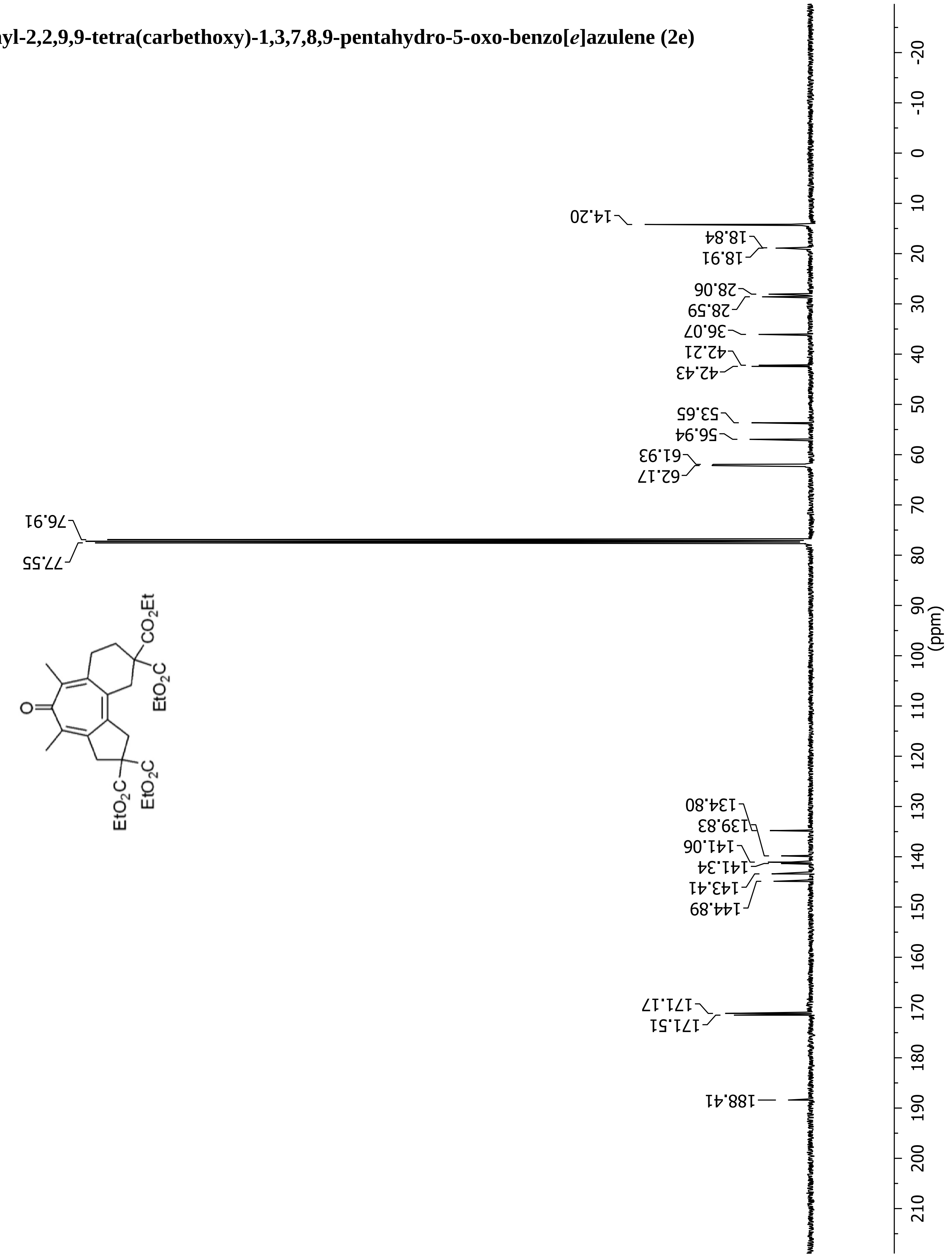

4-Phenyl-6-methyl-2,2,9,9-tetra(carbethoxy)-1,3,7,8,9-pentahydro-5-oxo-benzo[*e*]azulene (2f)

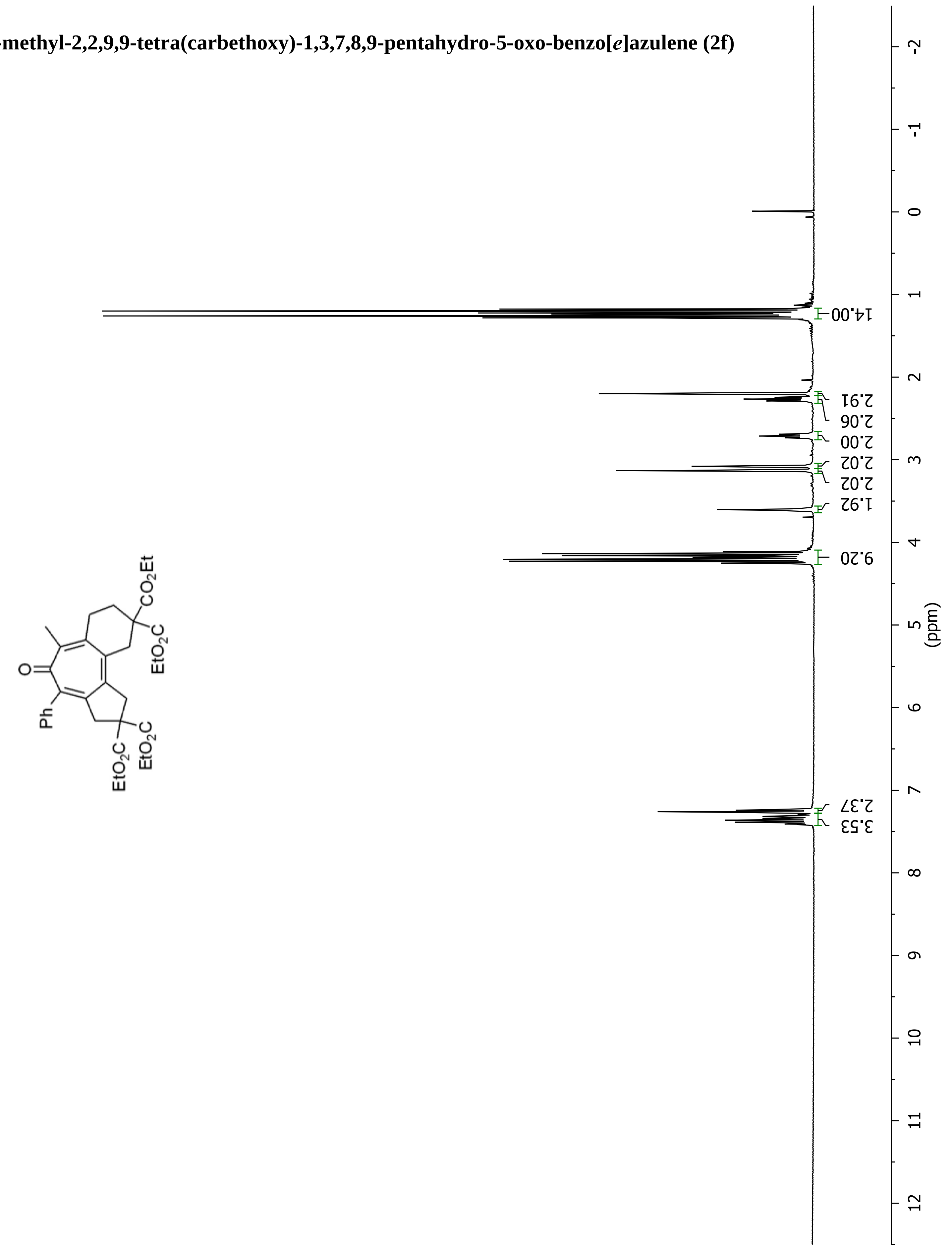

4-Phenyl-6-methyl-2,2,9,9-tetra(carbethoxy)-1,3,7,8,9-pentahydro-5-oxo-benzo[*e*]azulene (2f)

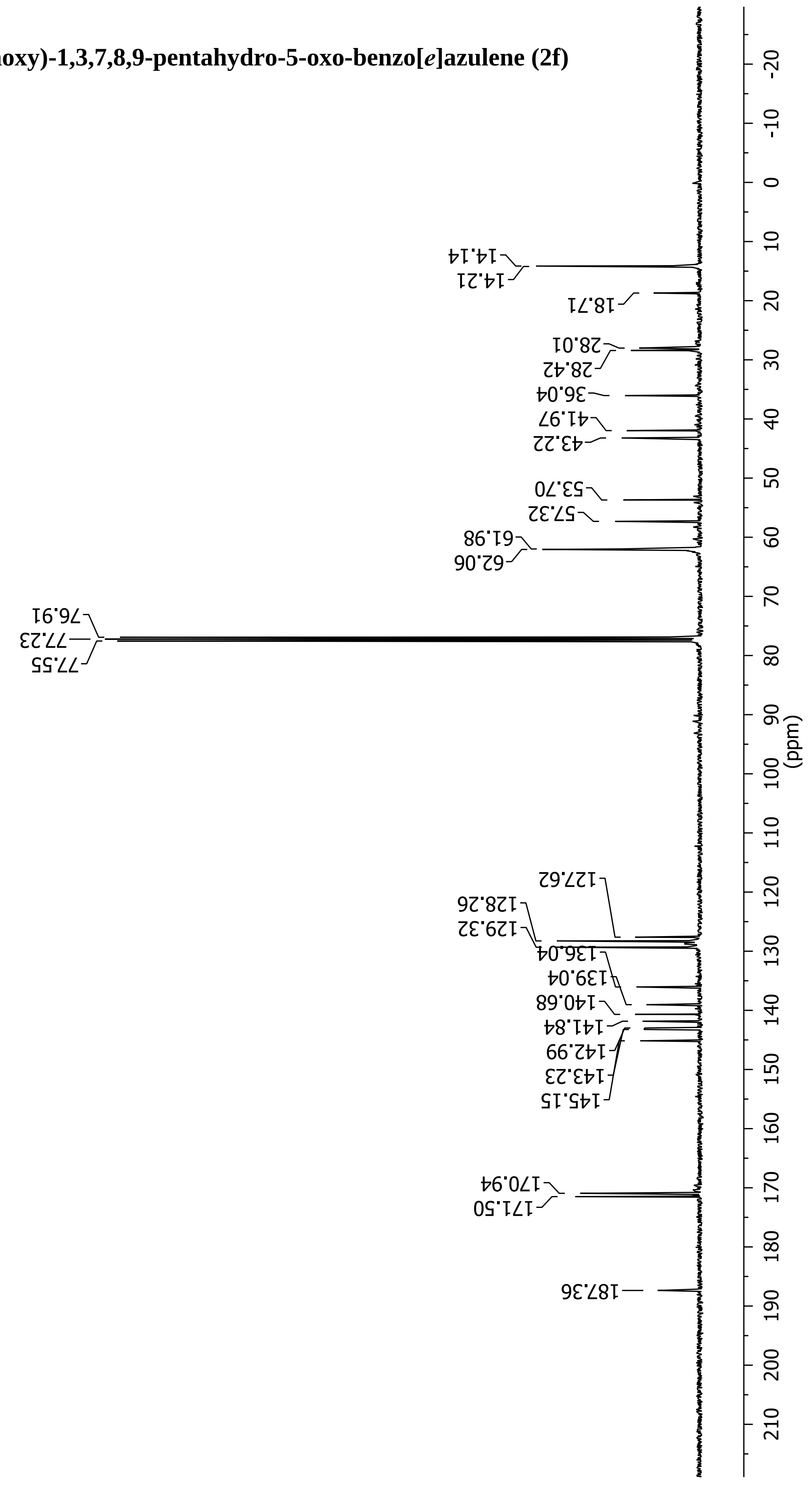

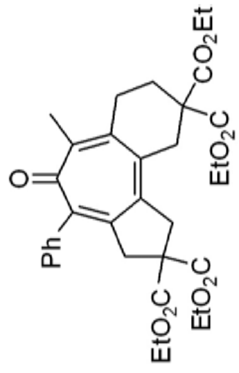

4-Phenyl-6-methyl-2,2-di(carbethoxy)-9-(4-methylbenzenesulfonyl)-1,3,7,8,9-pentahydro-5-oxo-9-aza-benzo[*e*]azulene (2g)

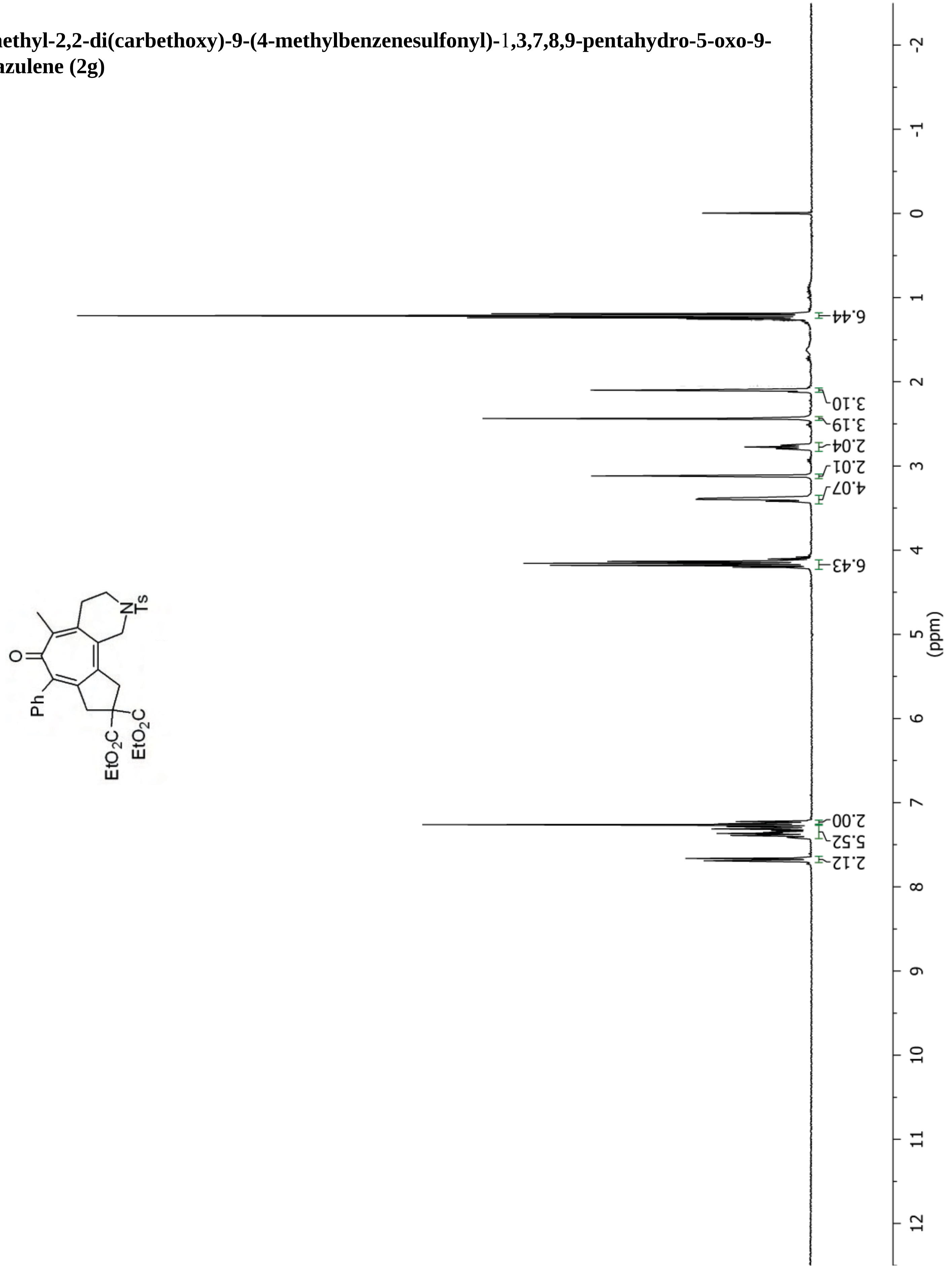

4-Phenyl-6-methyl-2,2-di(carbethoxy)-9-(4-methylbenzenesulfonyl)-1,3,7,8,9-pentahydro-5-oxo-9-aza-benzo[*e*]azulene (2g)

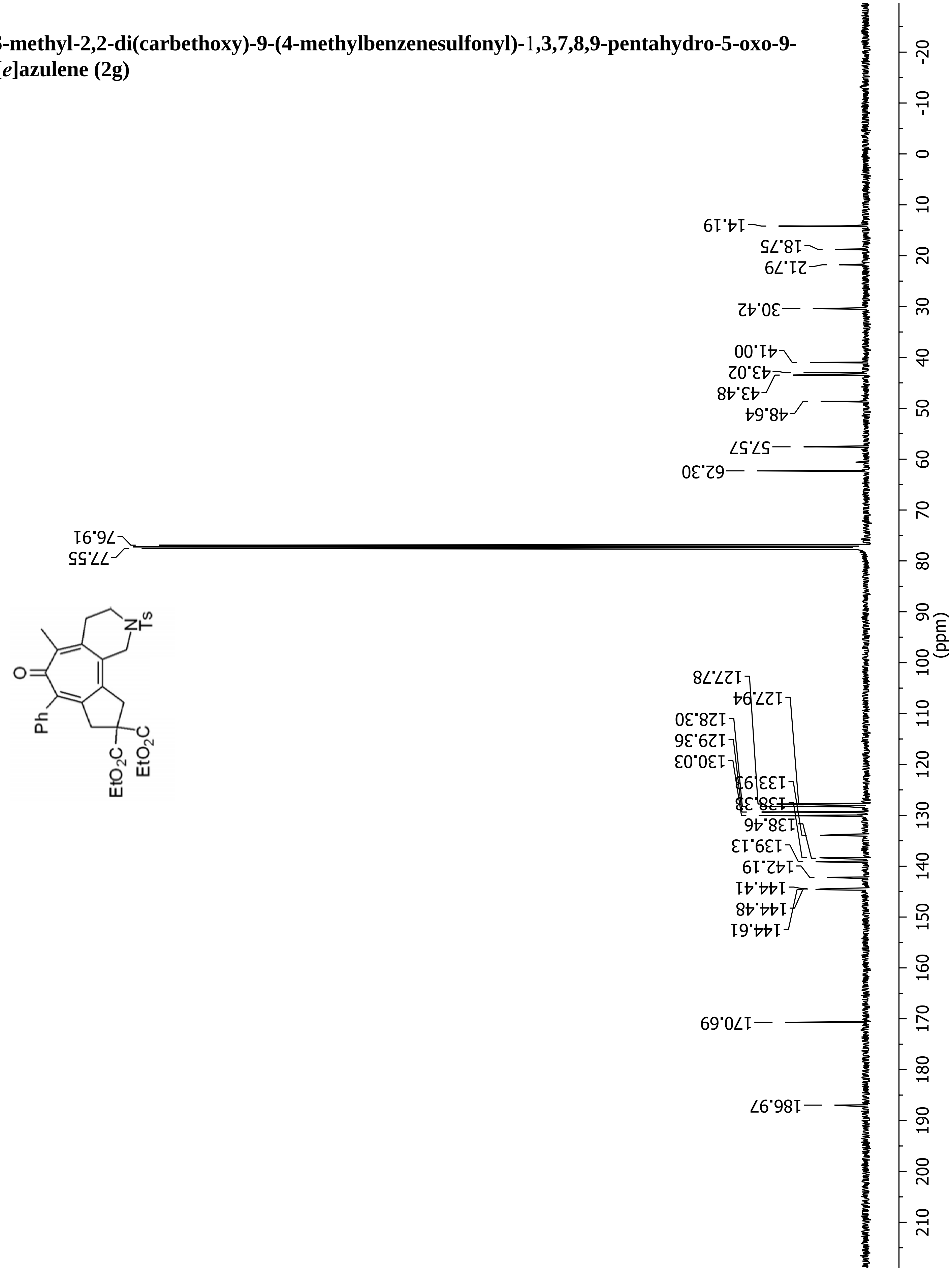

10,10-Di(carbethoxy)-6,8-dimethyl-7-oxo-2-(4-methylbenzenesulfonyl)-2,3,4,5,9,11-hexahydro-1*H*-azuleno[4,5-*c*]azepine (2h)

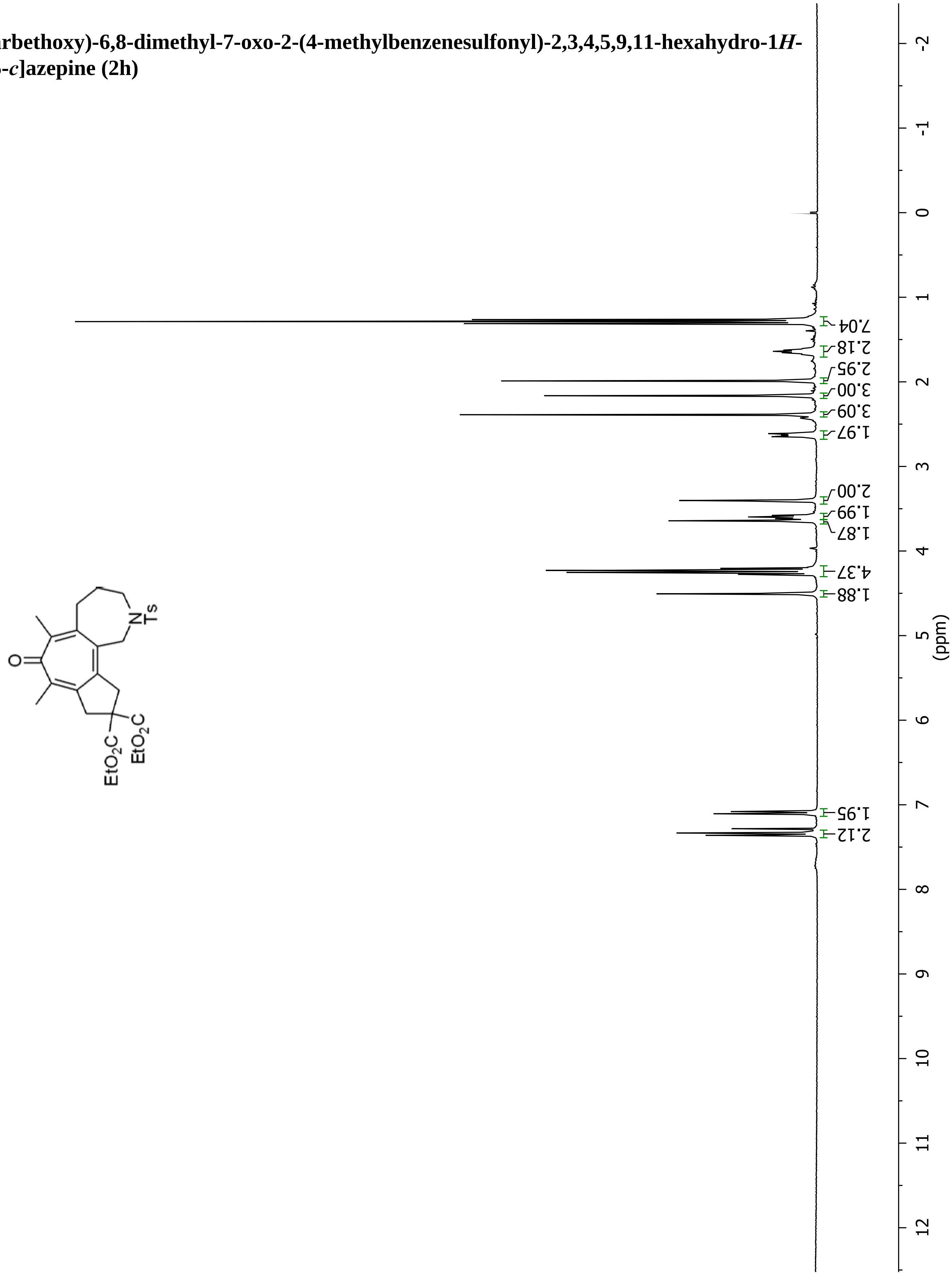

10,10-Di(carbethoxy)-6,8-dimethyl-7-oxo-2-(4-methylbenzenesulfonyl)-2,3,4,5,9,11-hexahydro-1*H*-azuleno[4,5-*c*]azepine (2h)

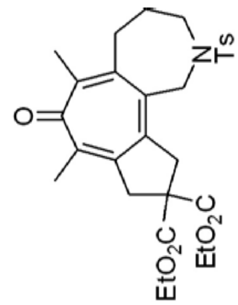

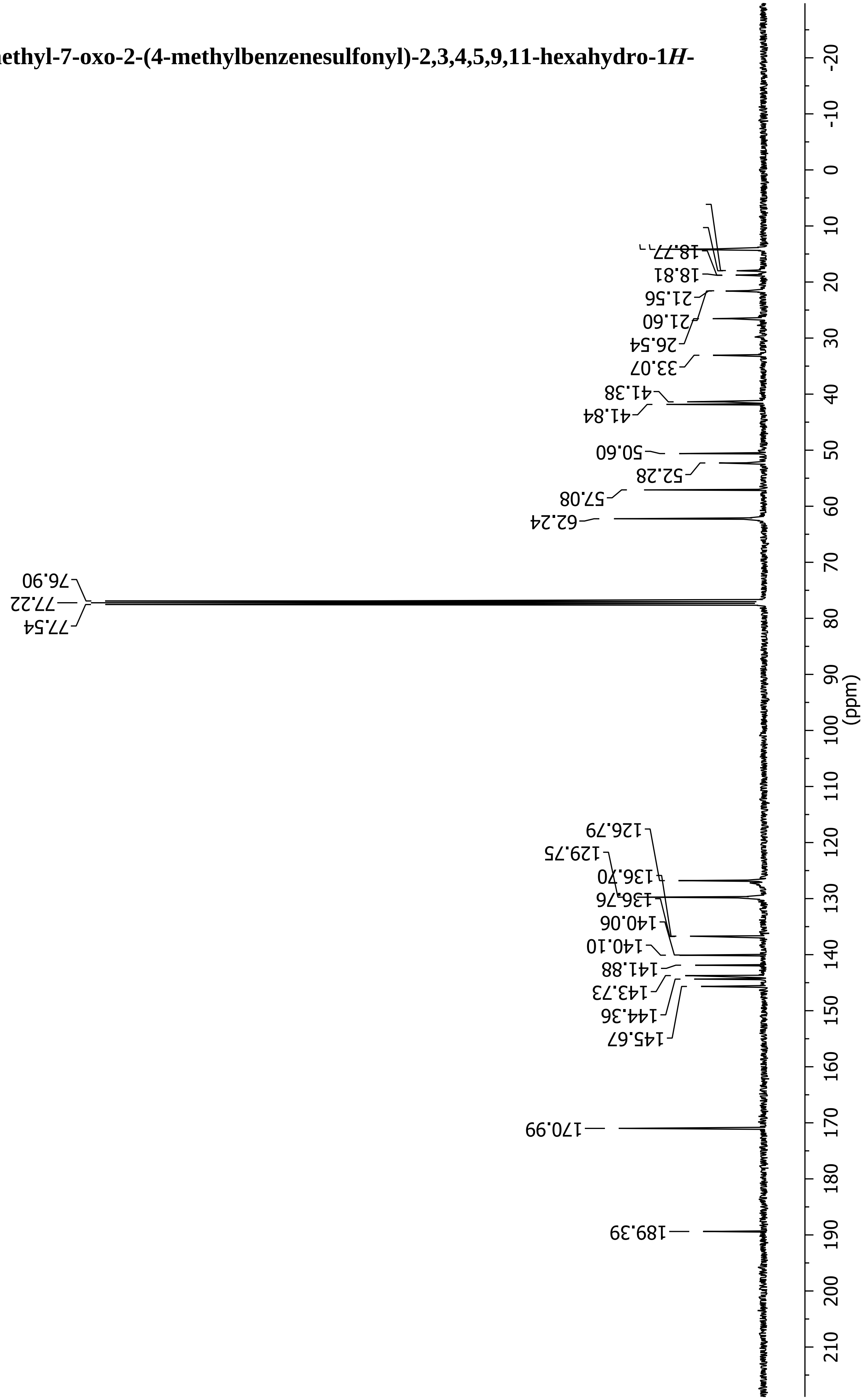

10,10-Di(carbethoxy)-6-methyl-8-phenyl-7-oxo-2-(4-methylbenzenesulfonyl)-2,3,4,5,9,11-hexahydro-1*H*-azuleno[4,5-*c*]azepine (2i)

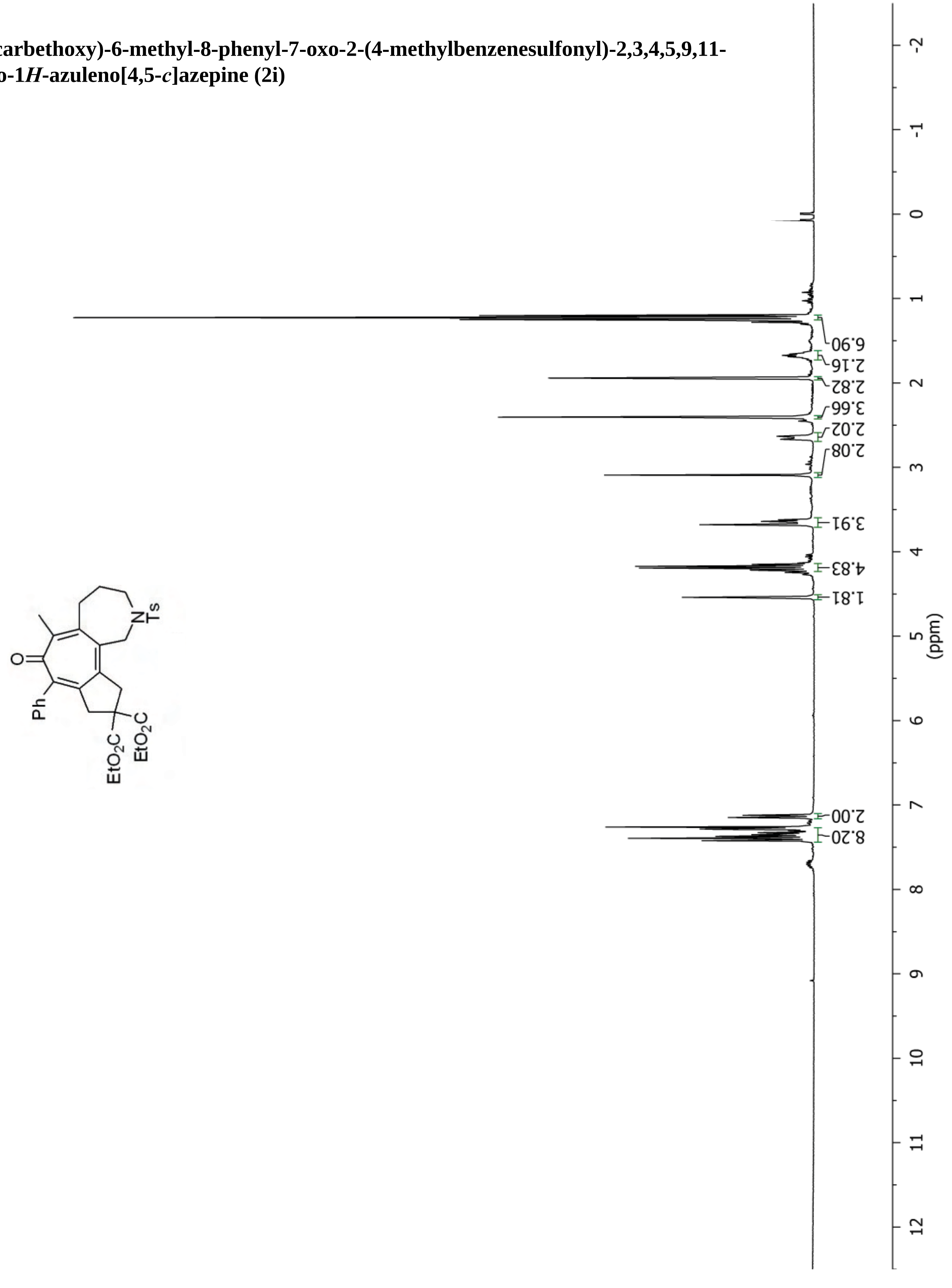

10,10-Di(carbethoxy)-6-methyl-8-phenyl-7-oxo-2-(4-methylbenzenesulfonyl)-2,3,4,5,9,11-hexahydro-1*H*-azuleno[4,5-*c*]azepine (2i)

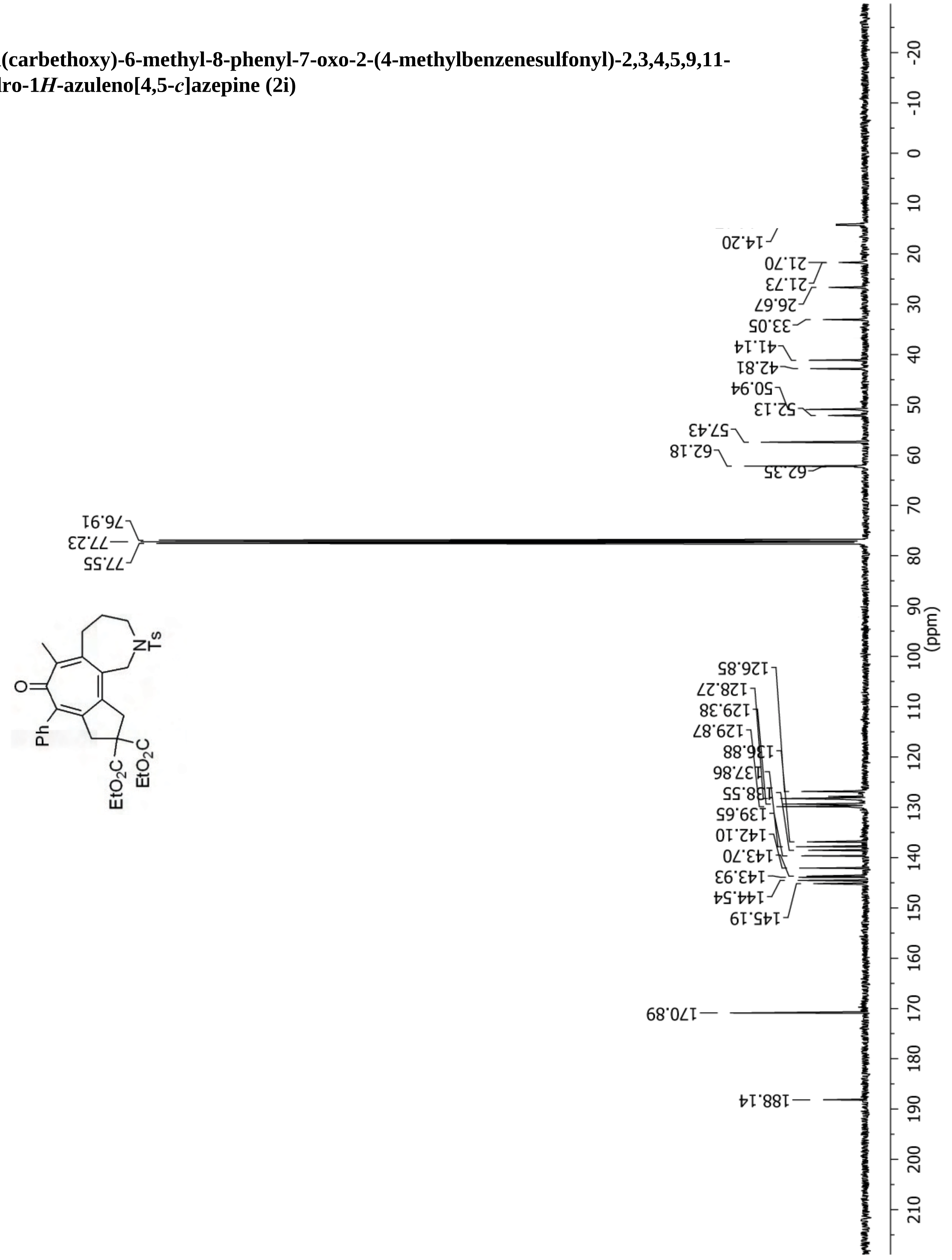

4,5-Dimethyl-2,2,7,7-tetra(carbethoxy)-1,3,6,8-tetrahydro-*as*-indacene (3b)

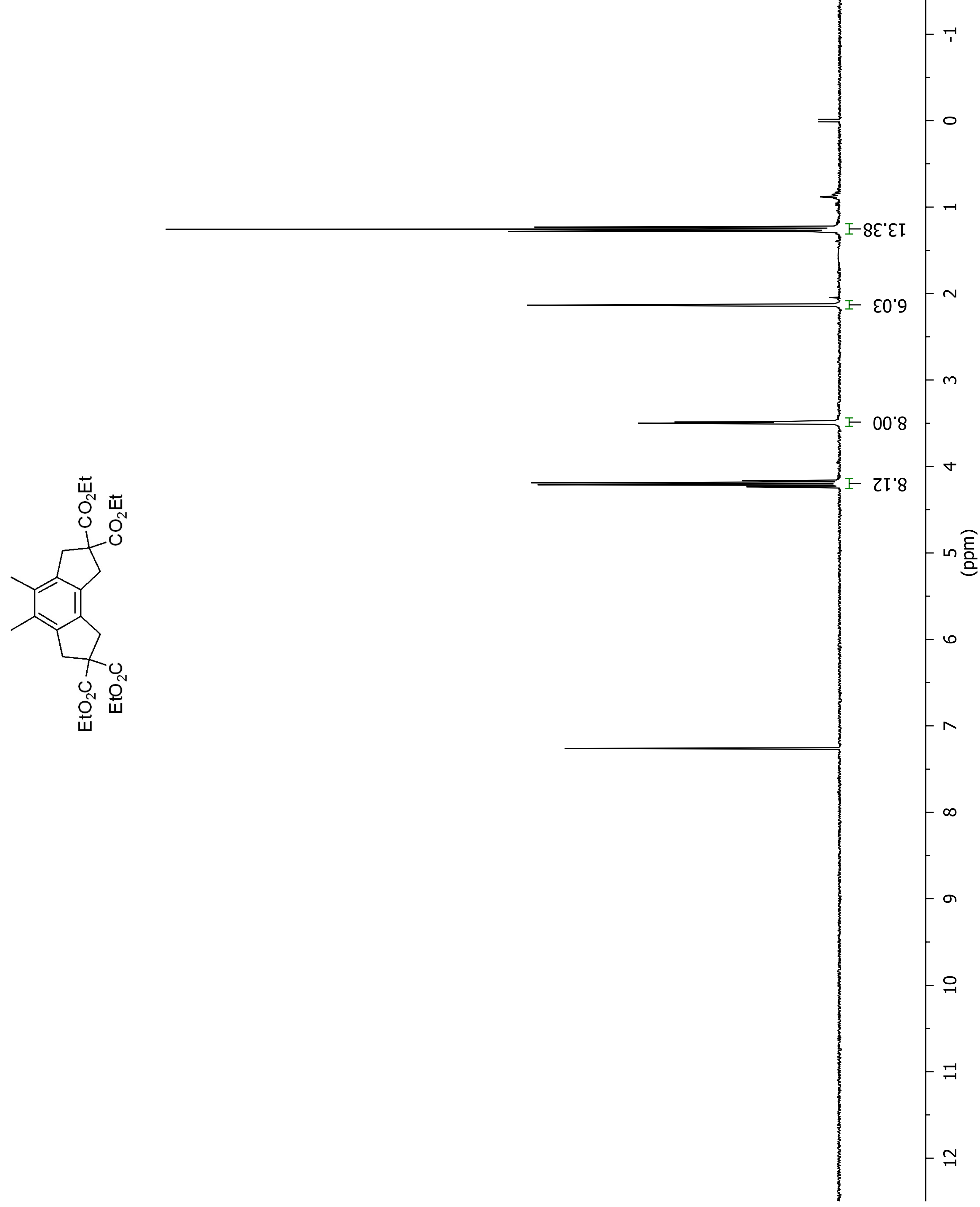

4,5-Dimethyl-2,2,7,7-tetra(carbethoxy)-1,3,6,8-tetrahydro-*as*-indacene (3b)

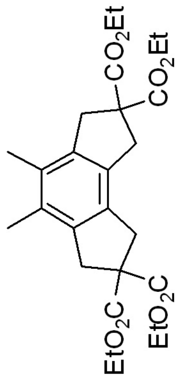

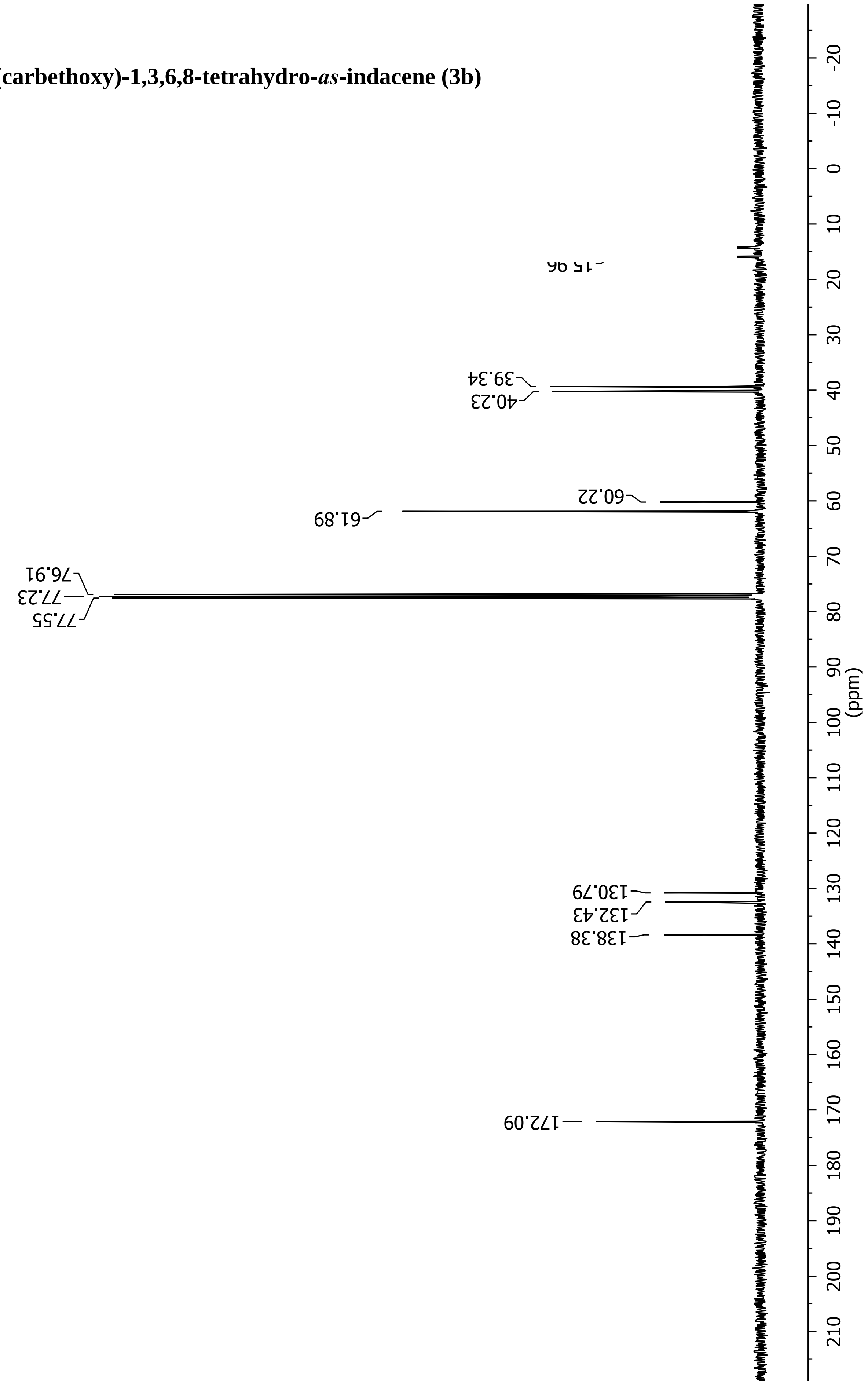

4,5-Dimethyl-1,3,6,8-tetrahydro-2,7-dioxa-*as*-indacene (3c)

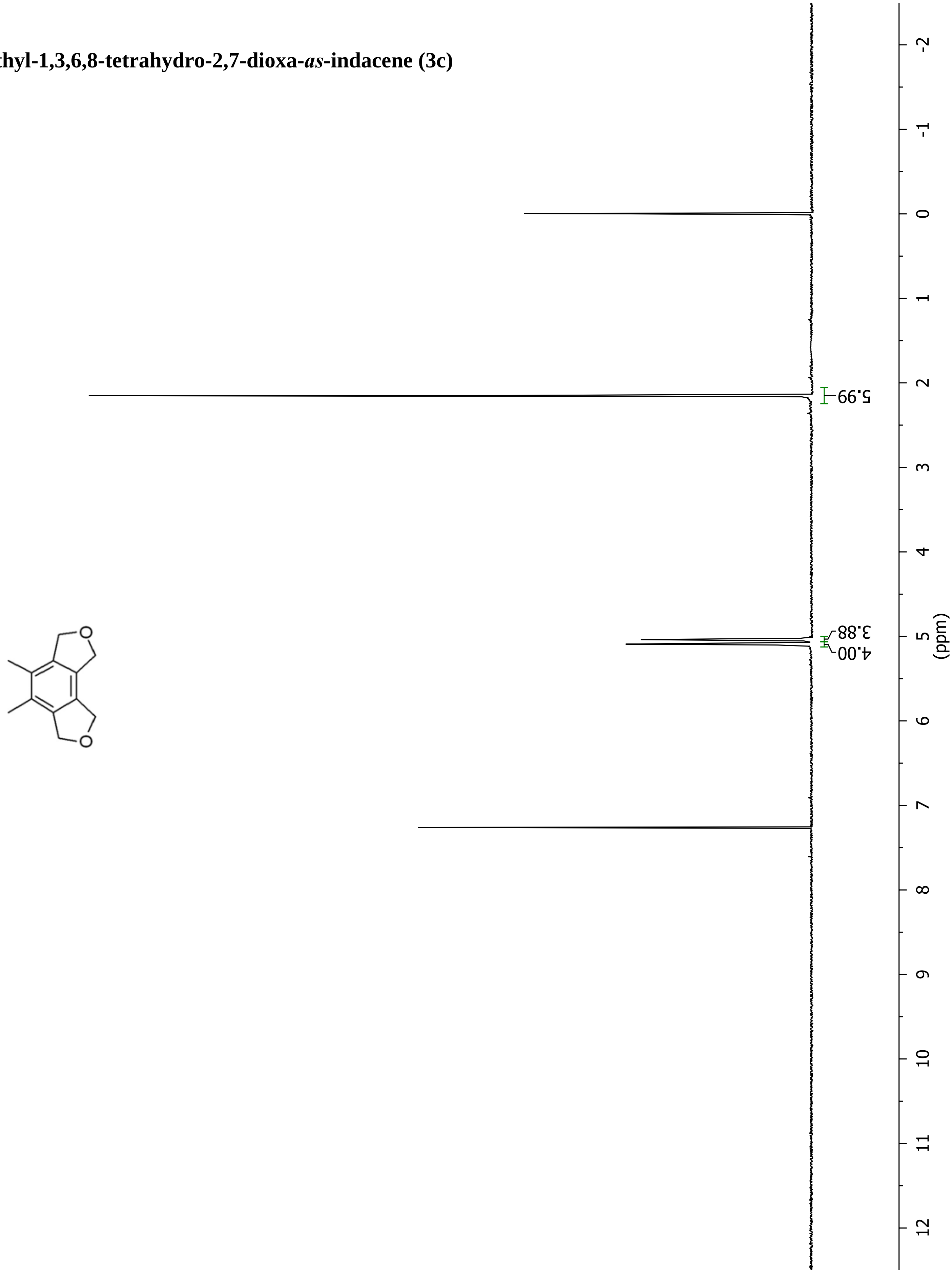

4,5-Dimethyl-1,3,6,8-tetrahydro-2,7-dioxa-*as*-indacene (3c)

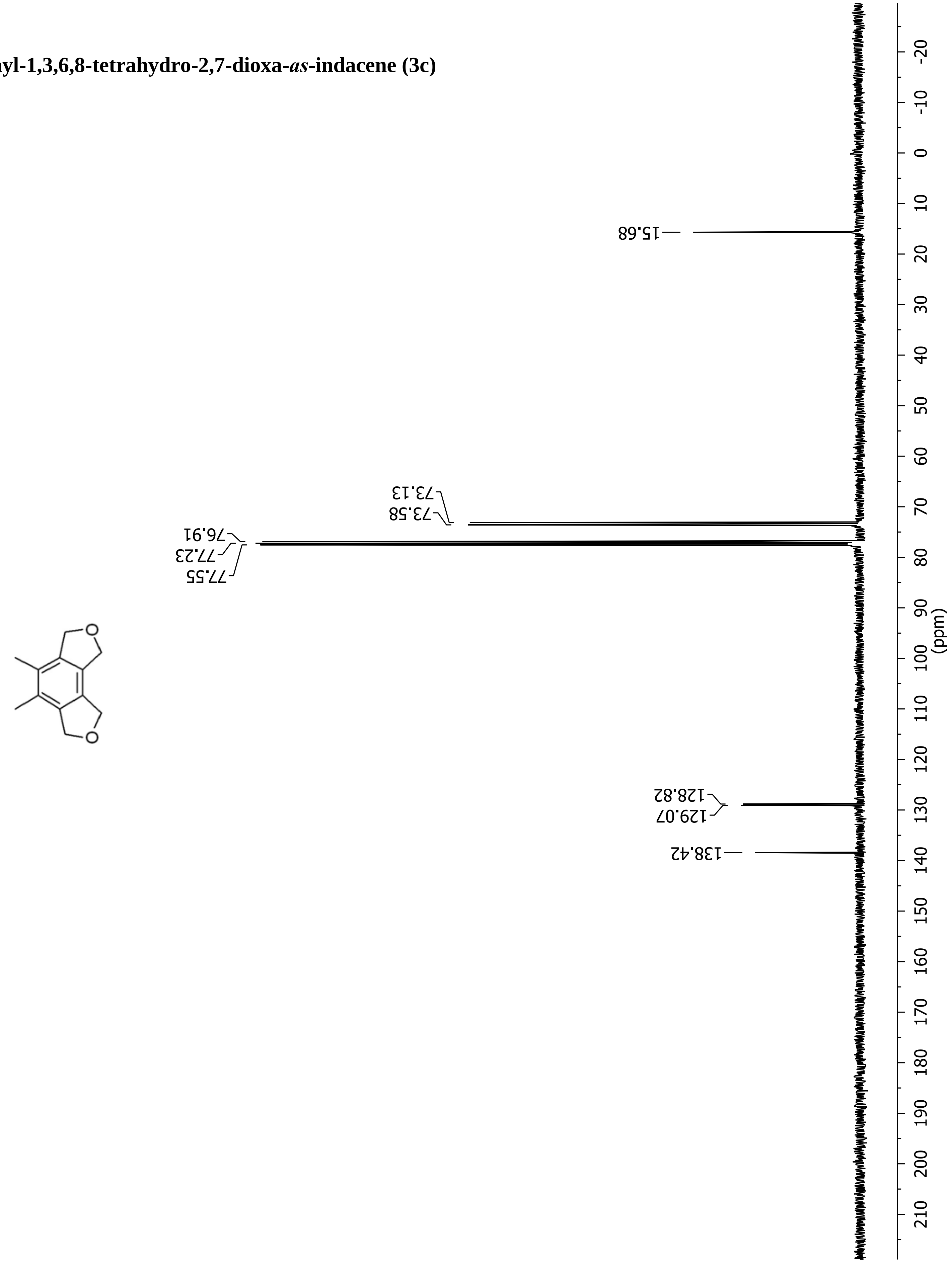

4,5-Dimethyl-7,7-di(carbethoxy)-1,3,6,8-tetrahydro-2-(4-Methylbenzenesulfonyl)-2-aza-*as*-indacene (3d)

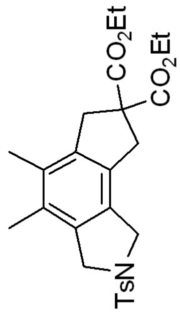

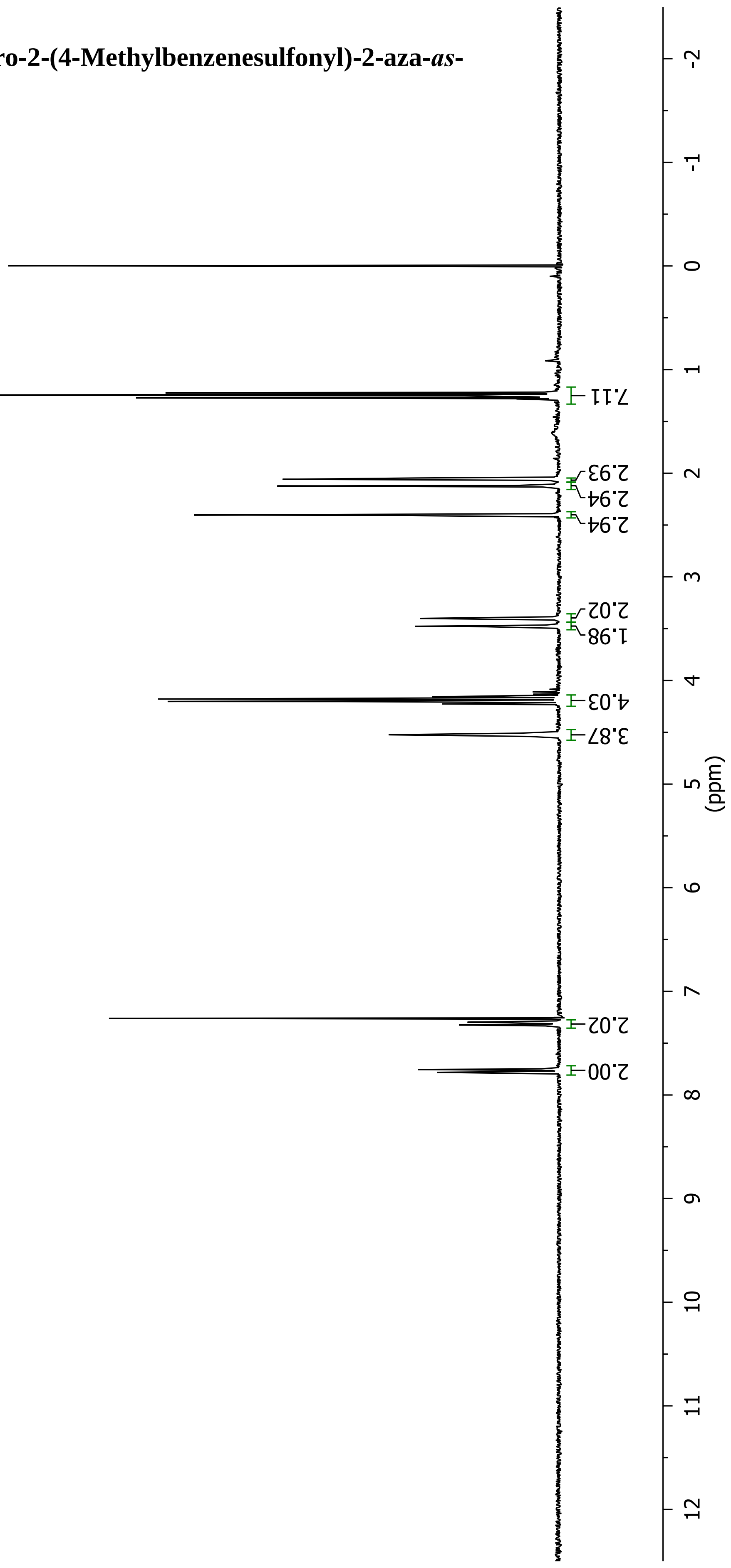

4,5-Dimethyl-7,7-di(carbethoxy)-1,3,6,8-tetrahydro-2-(4-Methylbenzenesulfonyl)-2-aza-*as*-indacene (3d)

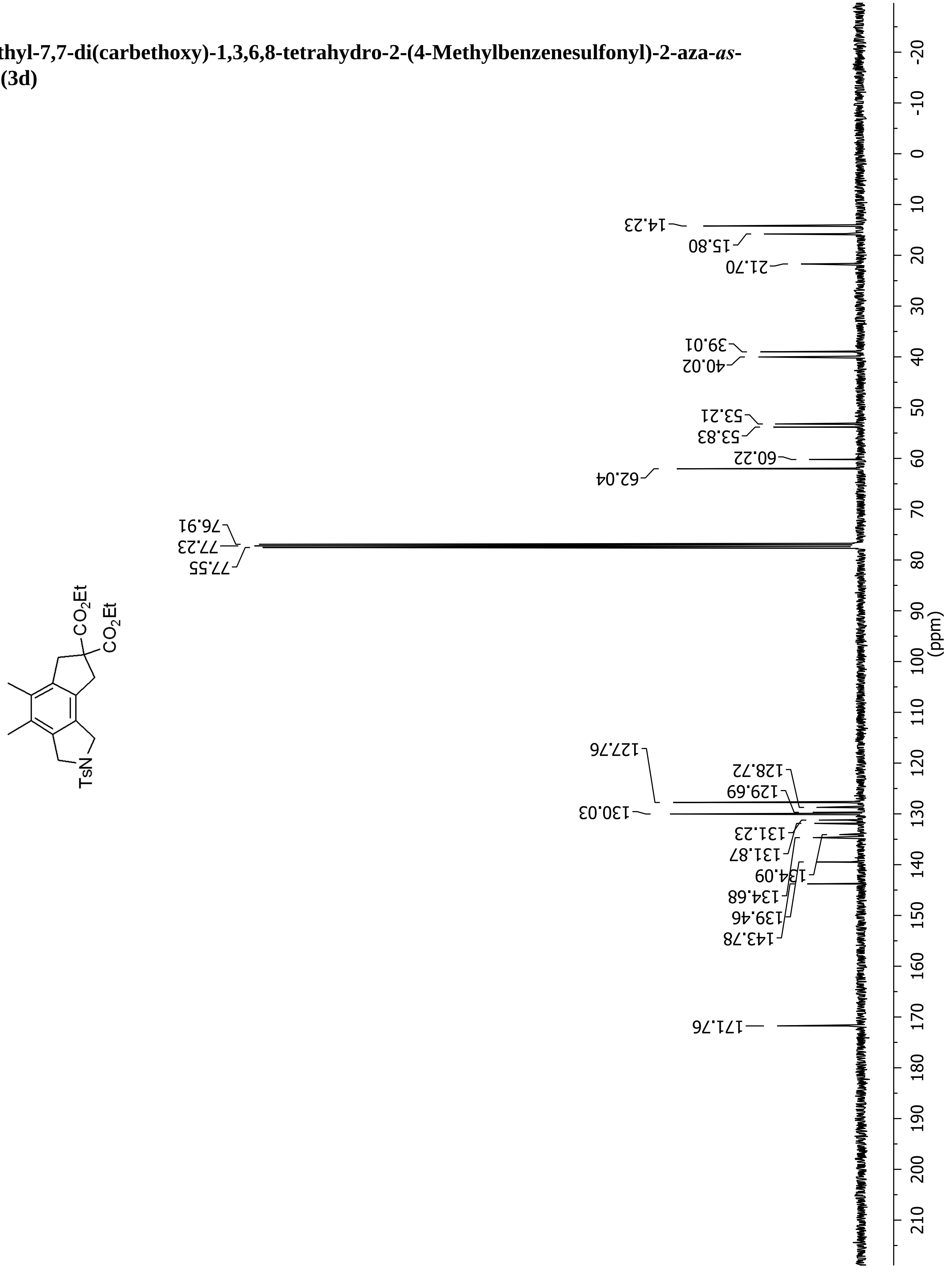

4,5-Dimethyl-2,2,8,8-tetra(carbethoxy)-3,6,7,8,9-pentahydro-1*H*-cyclopenta[*a*]naphthalene (3e)

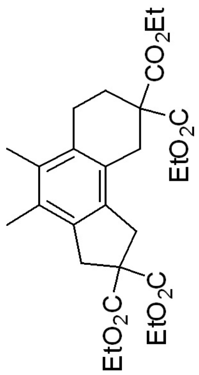

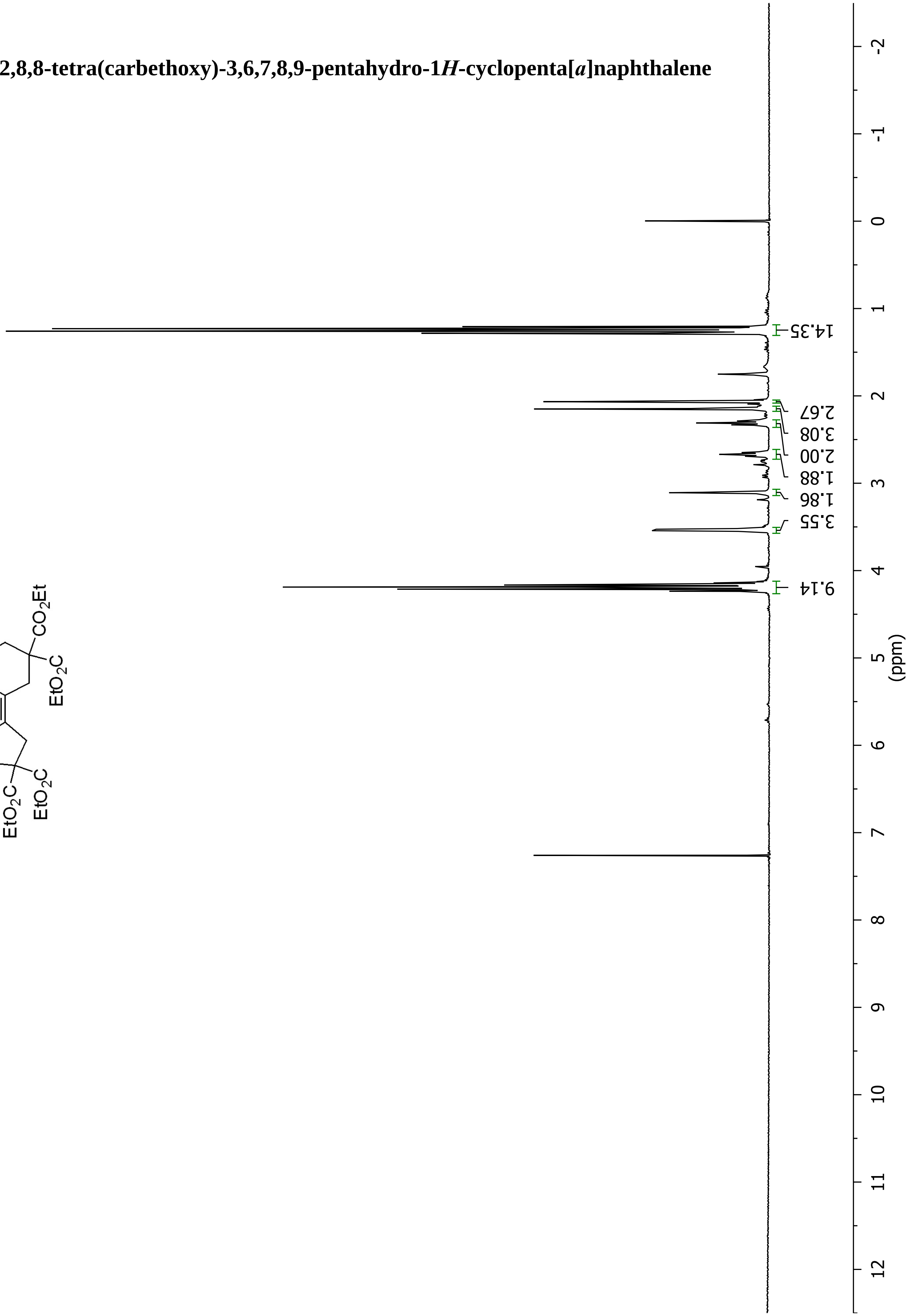

4,5-Dimethyl-2,2,8,8-tetra(carbethoxy)-3,6,7,8,9-pentahydro-1*H*-cyclopenta[*a*]naphthalene (3e)

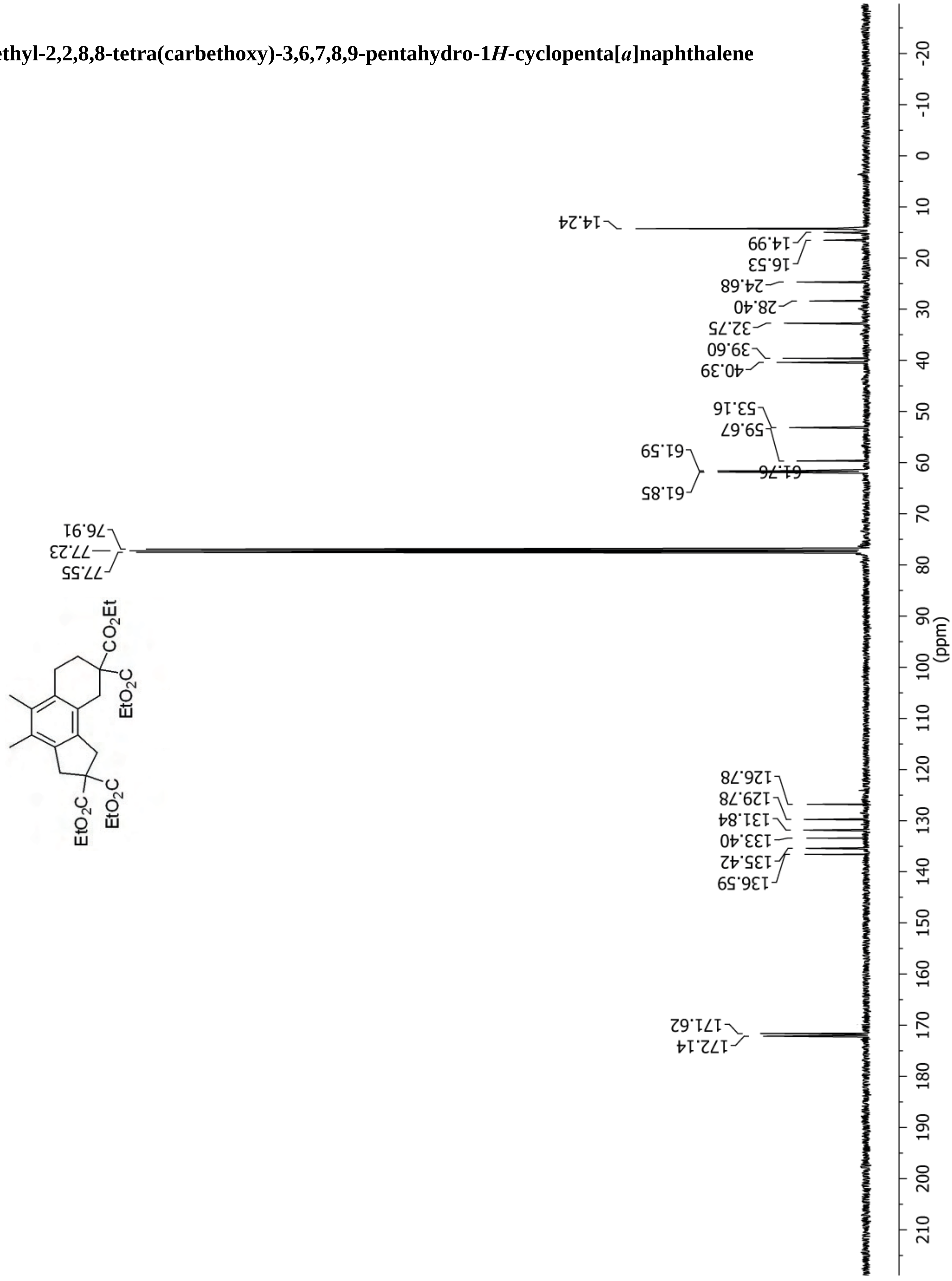

4-Phenyl-5-methyl-2,2,8,8-tetra(carbethoxy)-3,6,7,8,9-pentahydro-1*H*-cyclopenta[*a*] naphthalene (3f)

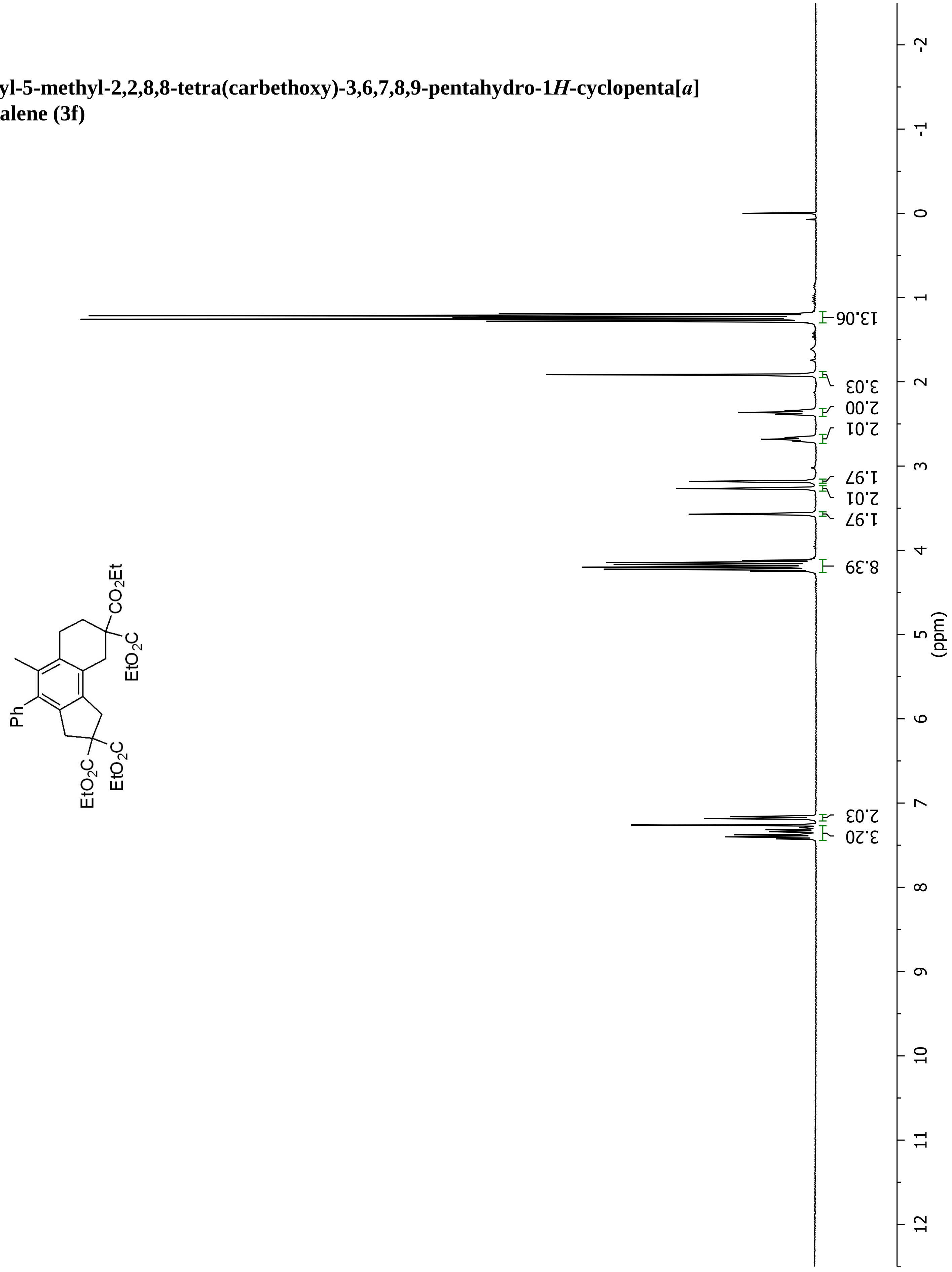

4-Phenyl-5-methyl-2,2,8,8-tetra(carbethoxy)-3,6,7,8,9-pentahydro-1*H*-cyclopenta[*a*] naphthalene (3f)

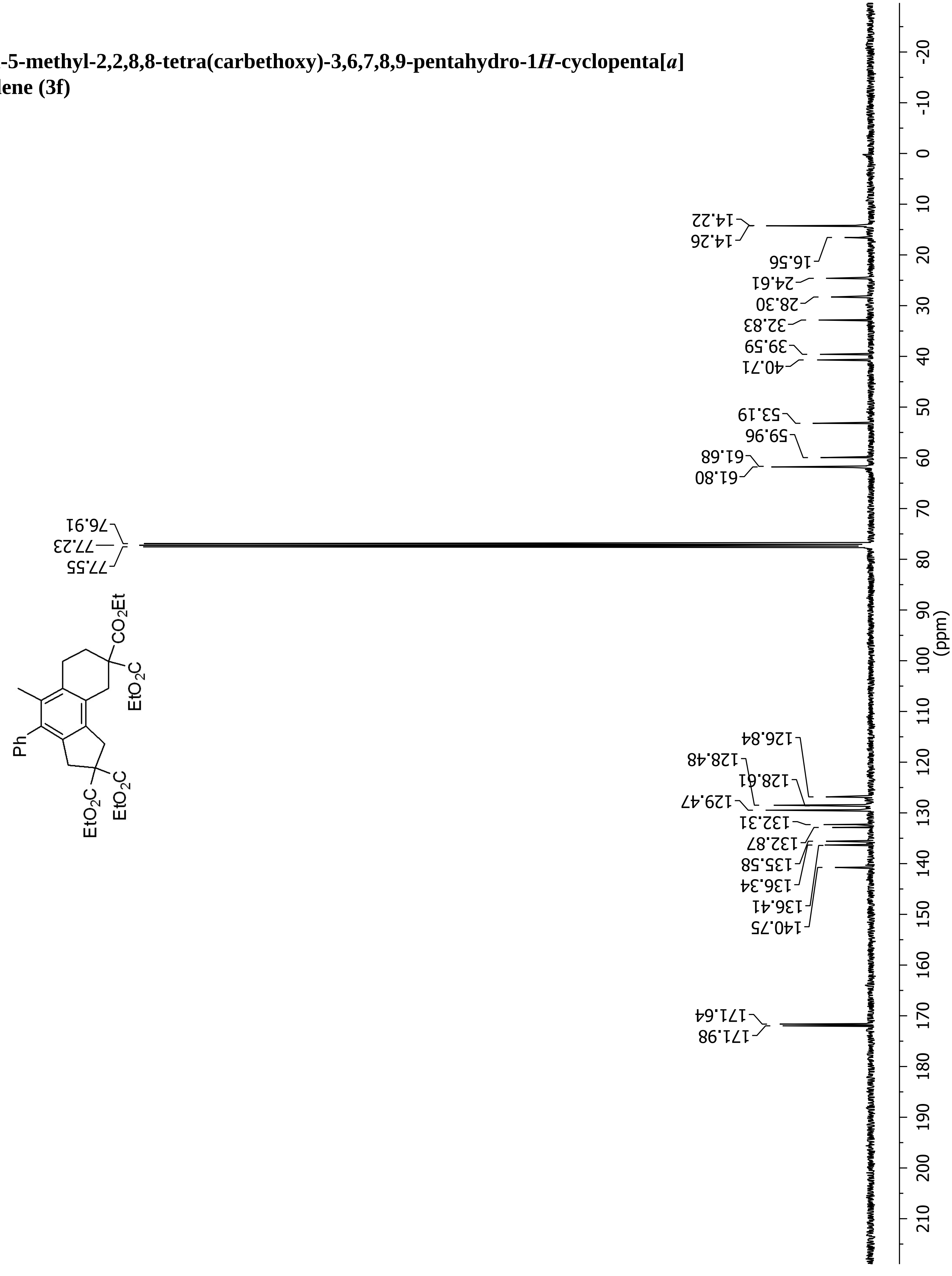

4-Phenyl-5-methyl-2,2-di(carbethoxy)-8-(4-methylbenzenesulfonyl)-3,6,7,8,9-pentahydro-8-aza-1*H*-cyclopenta[*a*]naphthalene (3g)

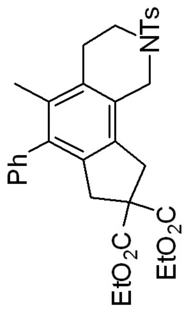

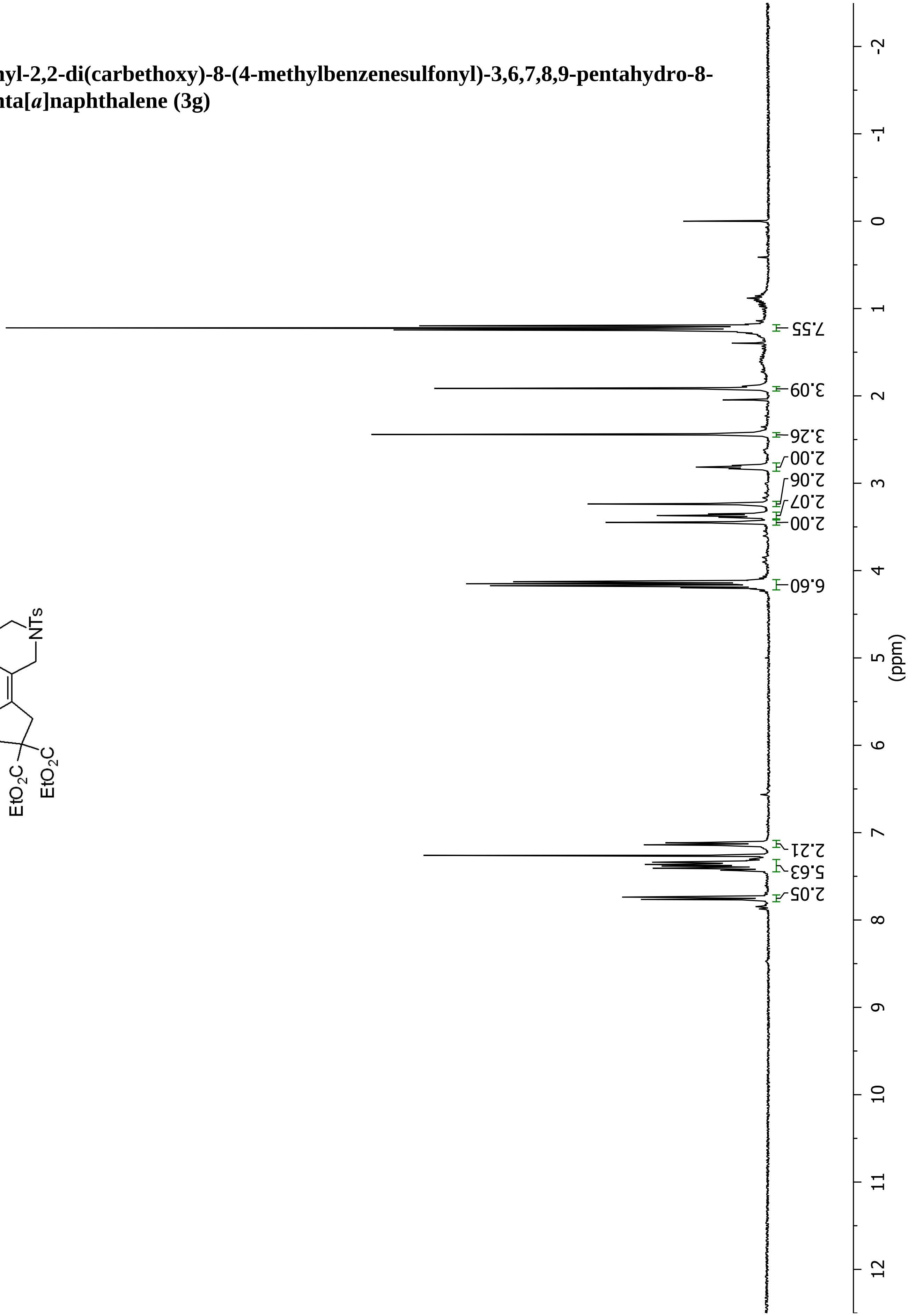

4-Phenyl-5-methyl-2,2-di(carbethoxy)-8-(4-methylbenzenesulfonyl)-3,6,7,8,9-pentahydro-8-aza-1*H*-cyclopenta[*a*]naphthalene (3g)

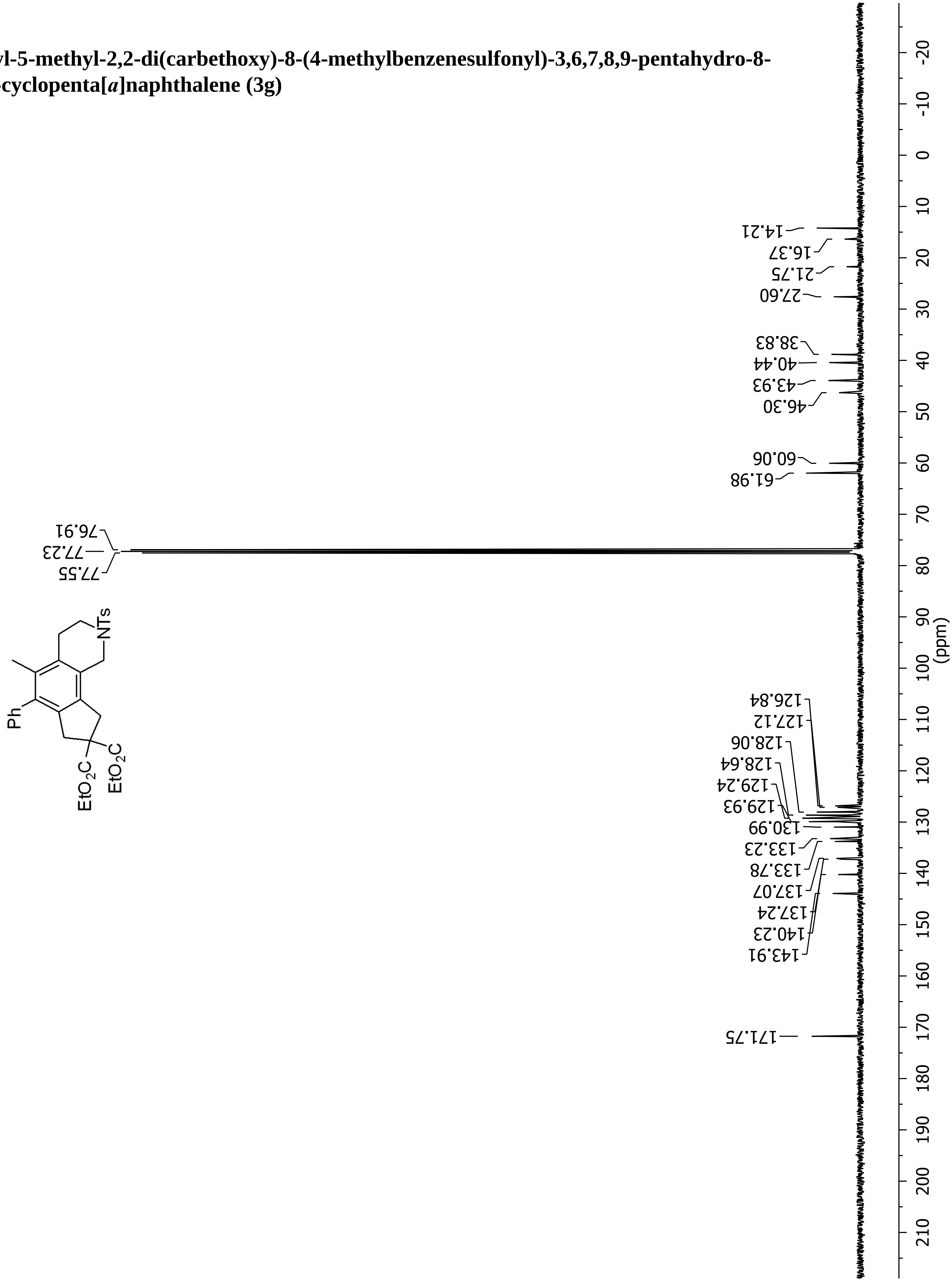

6,7-Dimethyl-9,9-di(carbethoxy)-2-(4-methylbenzenesulfonyl)-2,3,4,5,8,10-hexahydroindeno [4,5-*c*]azepine (3h)

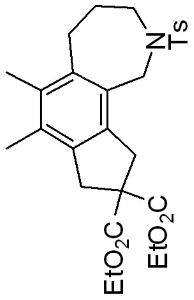

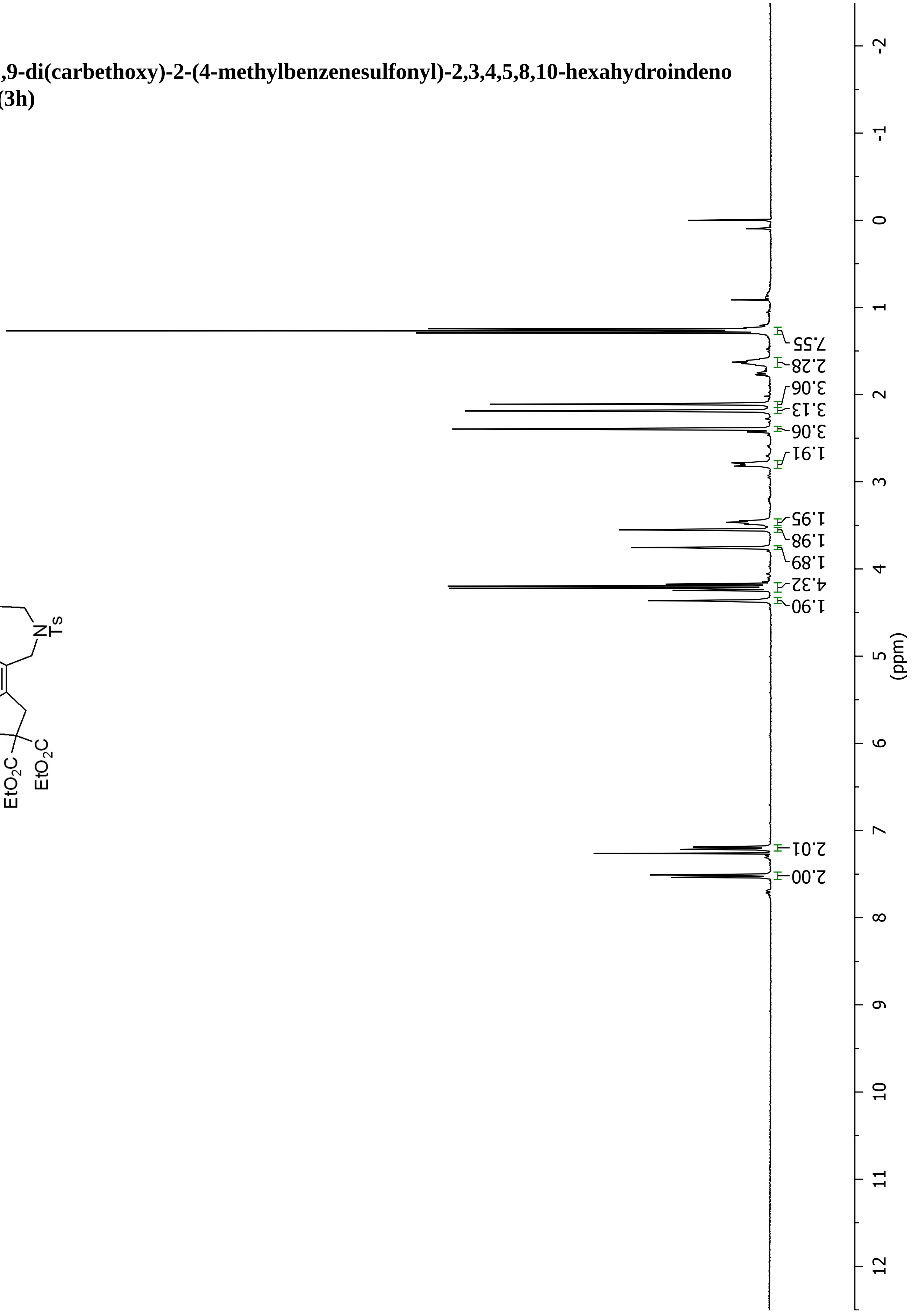

6,7-Dimethyl-9,9-di(carbethoxy)-2-(4-methylbenzenesulfonyl)-2,3,4,5,8,10-hexahydroindeno [4,5-*c*]azepine (3h)

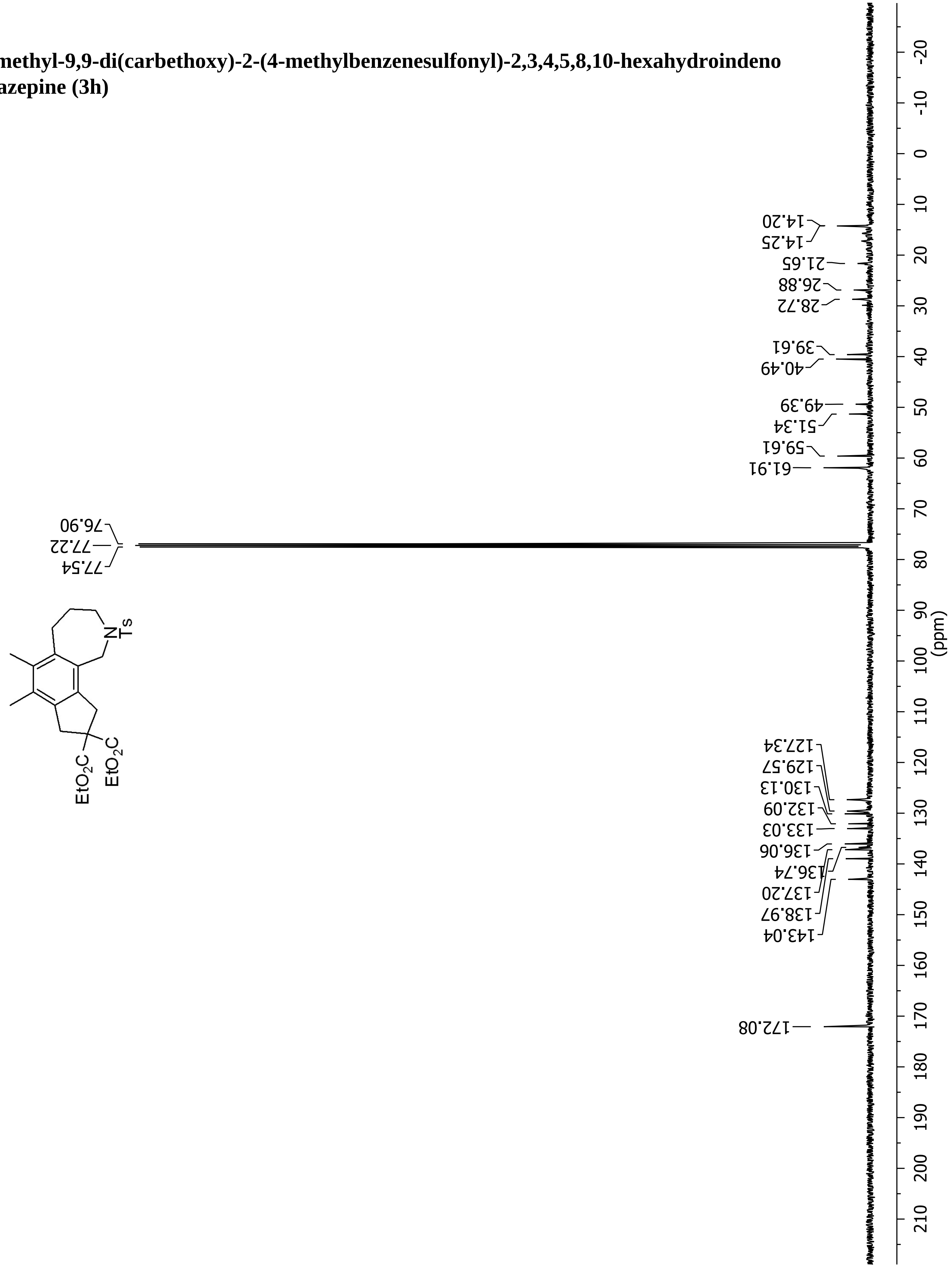

6-Methyl-7-phenyl-9,9-di(carbethoxy)-2-(4-methylbenzenesulfonyl)-2,3,4,5,8,10-hexahydroindeno[4,5-*c*]azepine (3i)

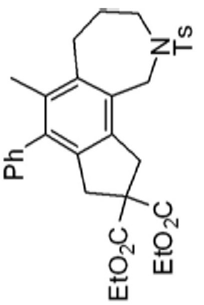

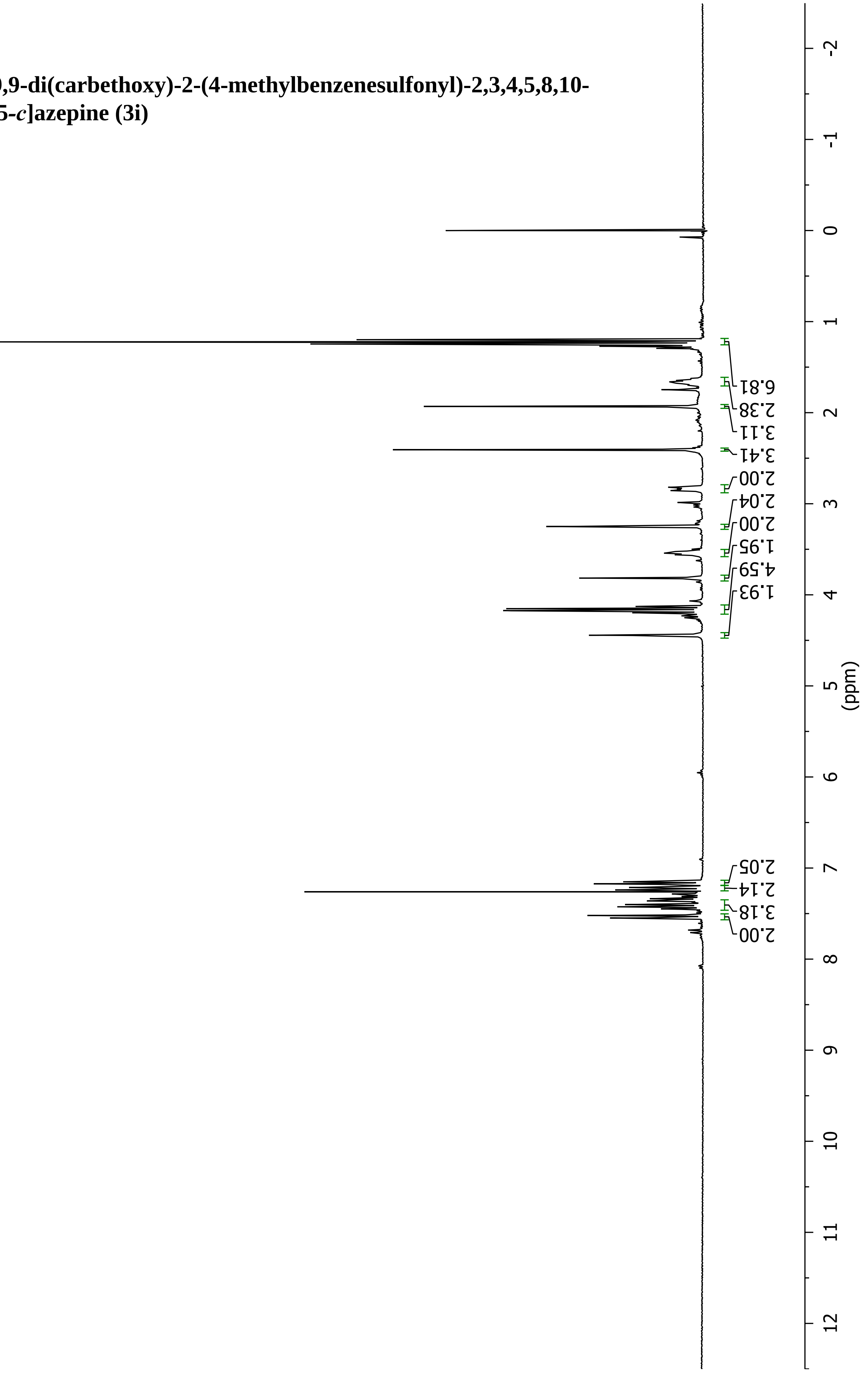

6-Methyl-7-phenyl-9,9-di(carbethoxy)-2-(4-methylbenzenesulfonyl)-2,3,4,5,8,10-hexahydroindeno[4,5-*c*]azepine (3i)

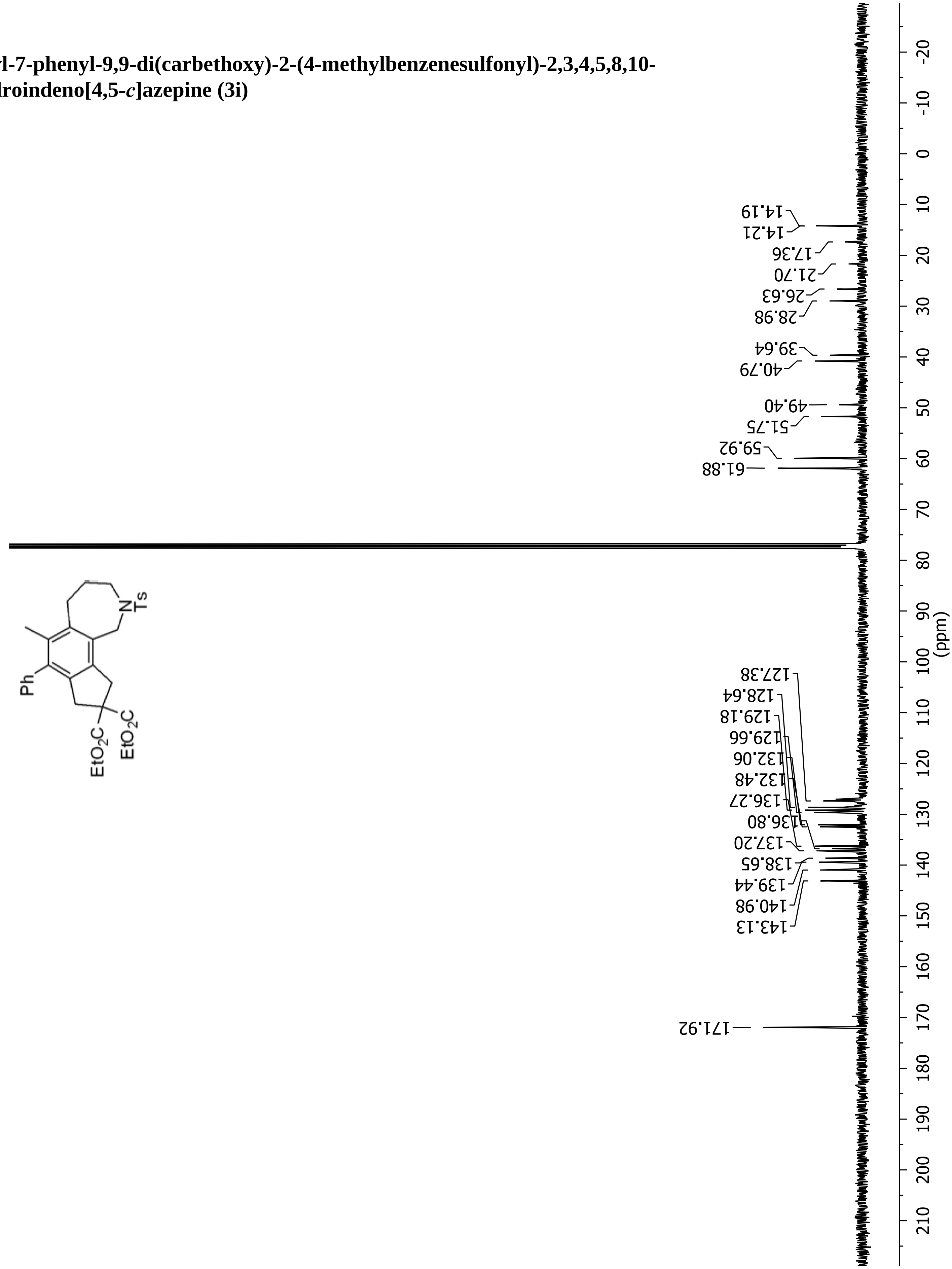

Diethyl 2-(but-2-ynyl)-2-{3-[2-(but-2-ynloxymethyl)phenyl]-prop-2-ynyl}malonate (4)

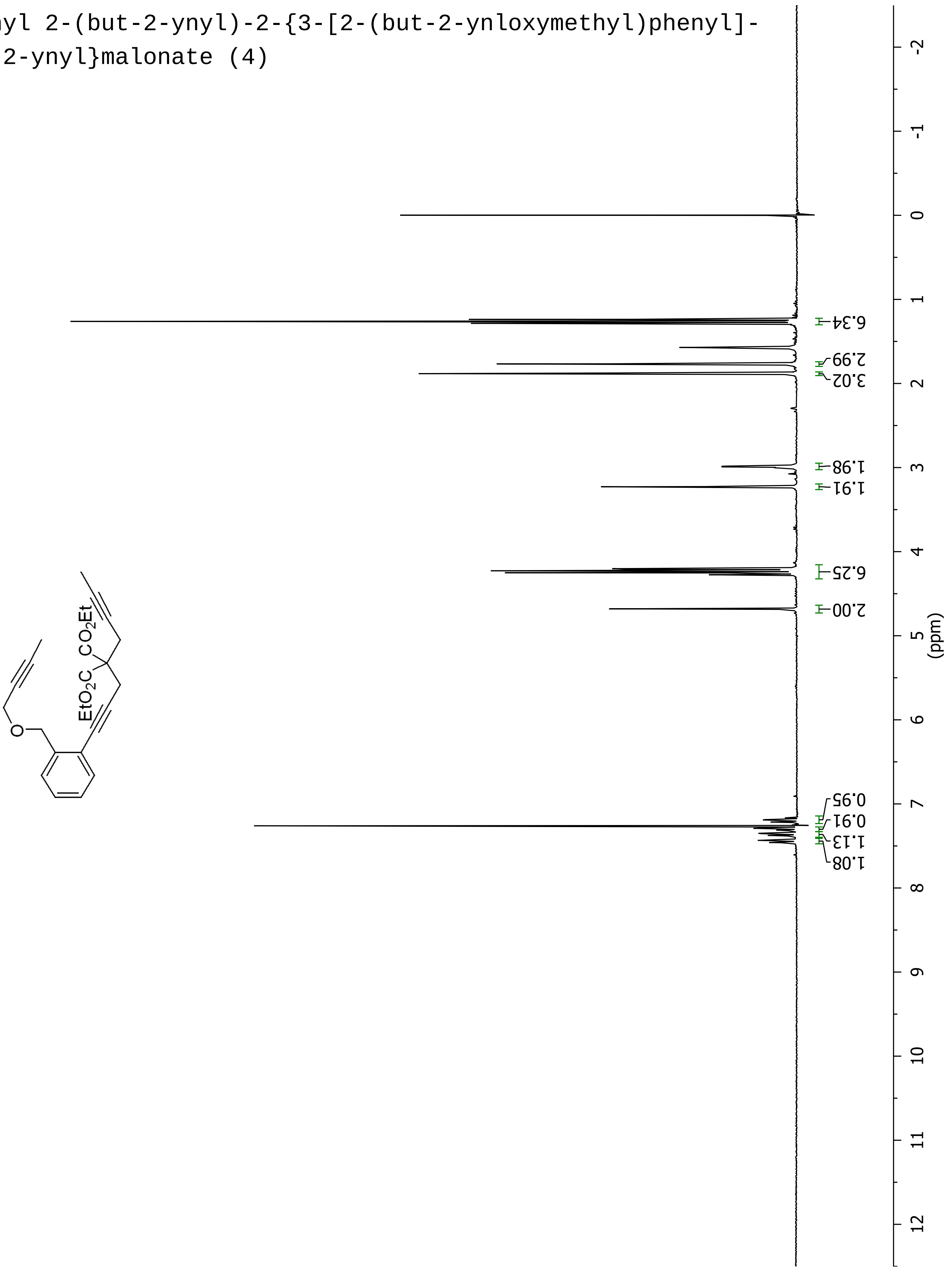

Diethyl 2-(but-2-ynyl)-2-{3-[2-(but-2-ynloxymethyl)phenyl]-prop-2-ynyl}malonate (4)

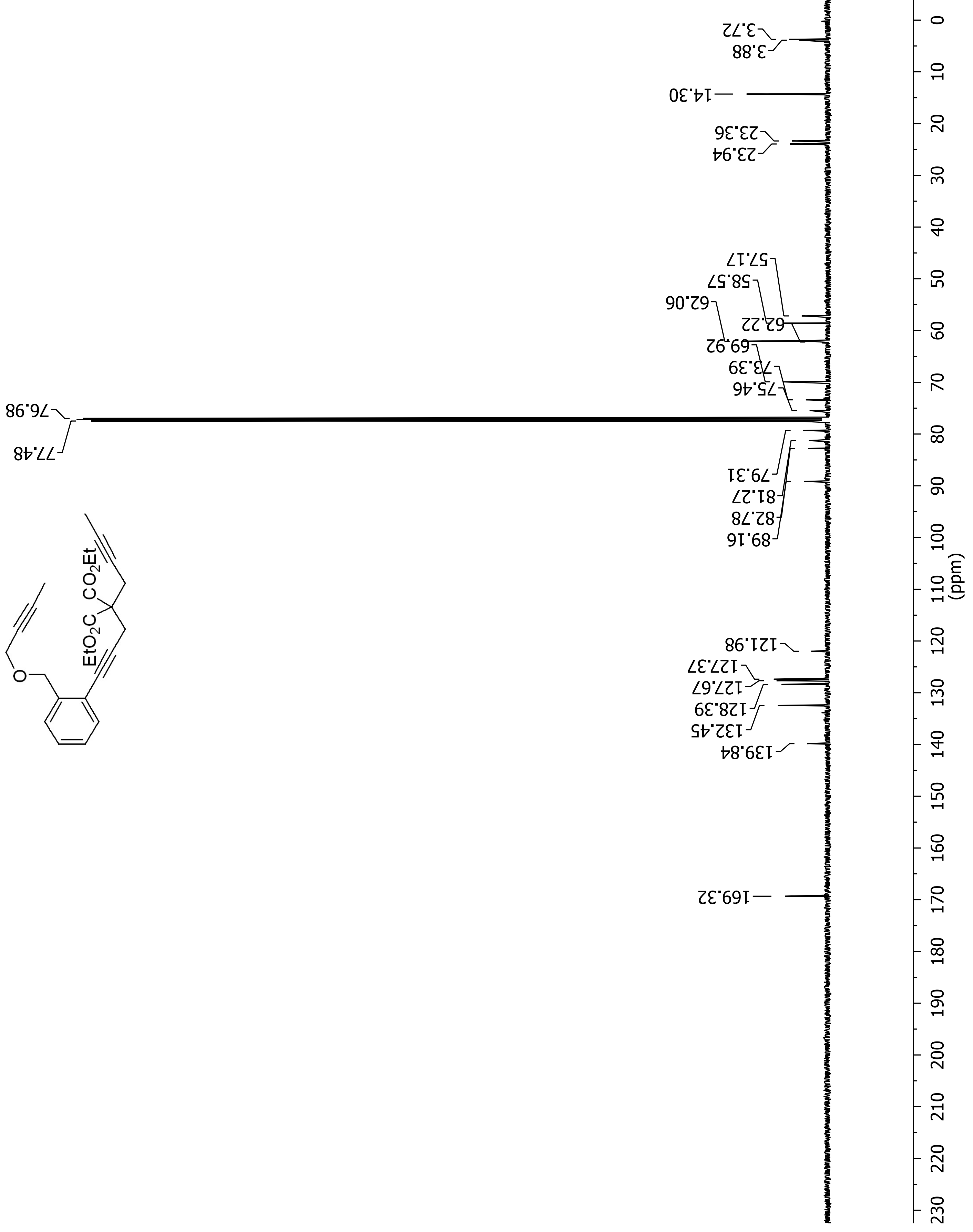

8,8-Di(carbethoxy)-4,6-dimethyl-5-oxo-2,3,7,9-tetrahydro-1H-azuleno[5,4-c]benzo[e]oxepine (5)

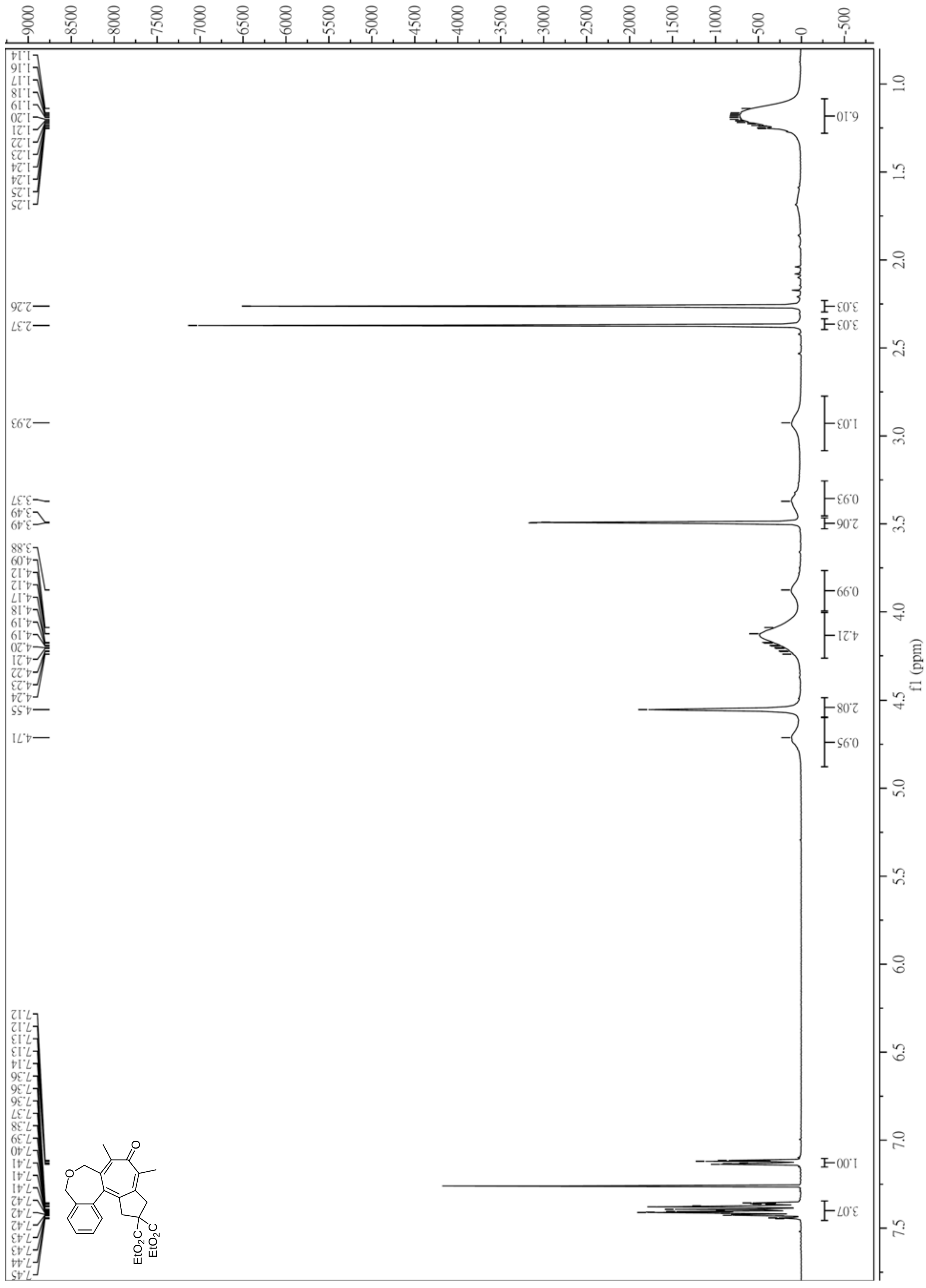

8,8-Di(carbethoxy)-4,6-dimethyl-5-oxo-2,3,7,9-tetrahydro-1H-azuleno[5,4-c]benzo[e]oxepine (5)

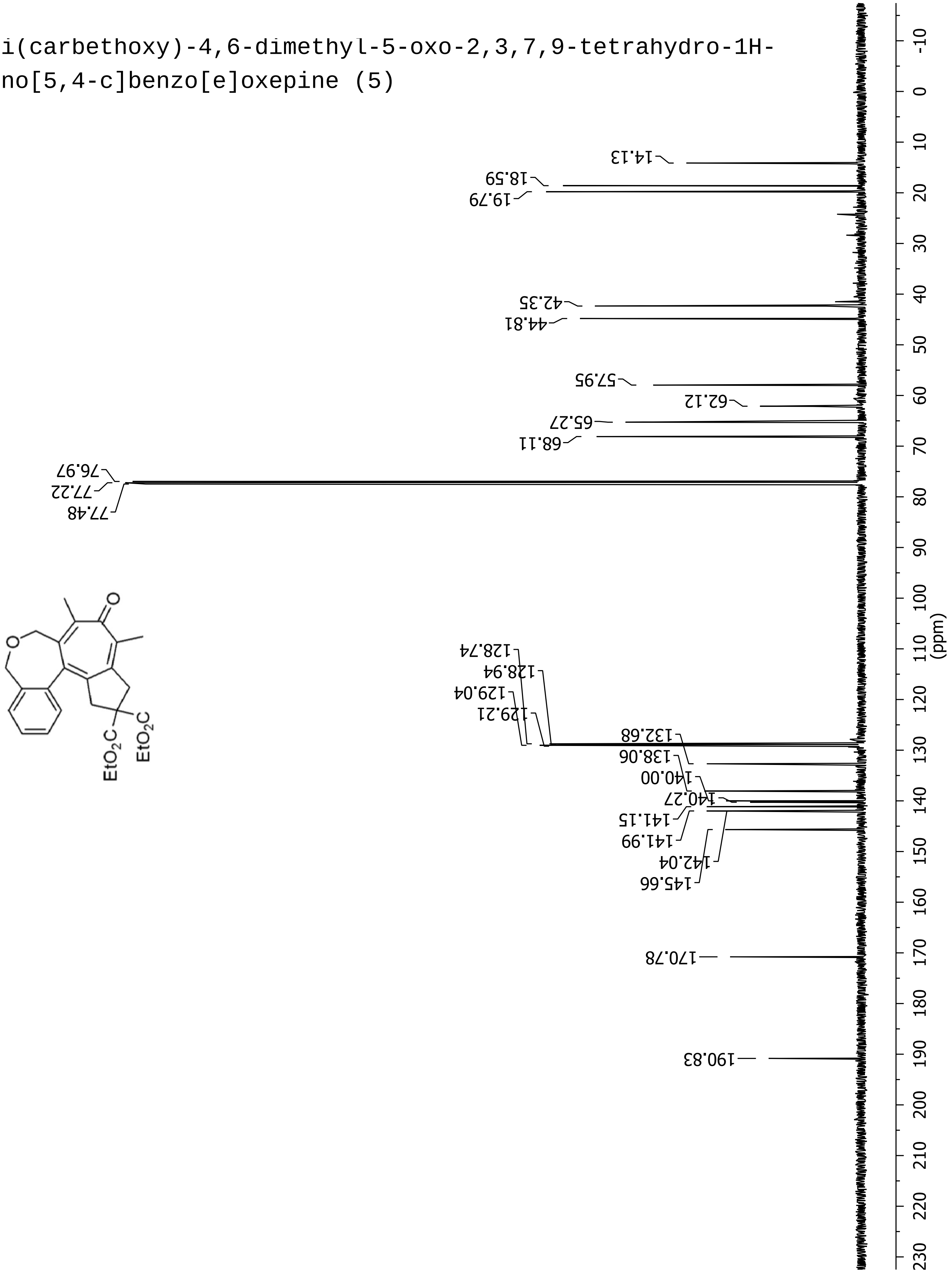

11,11-Di(carbethoxy)-8,9-dimethyl-7,10,11,12-tetrahydro-5H-benzo[e]indeno[5,4-c]oxepine (6)

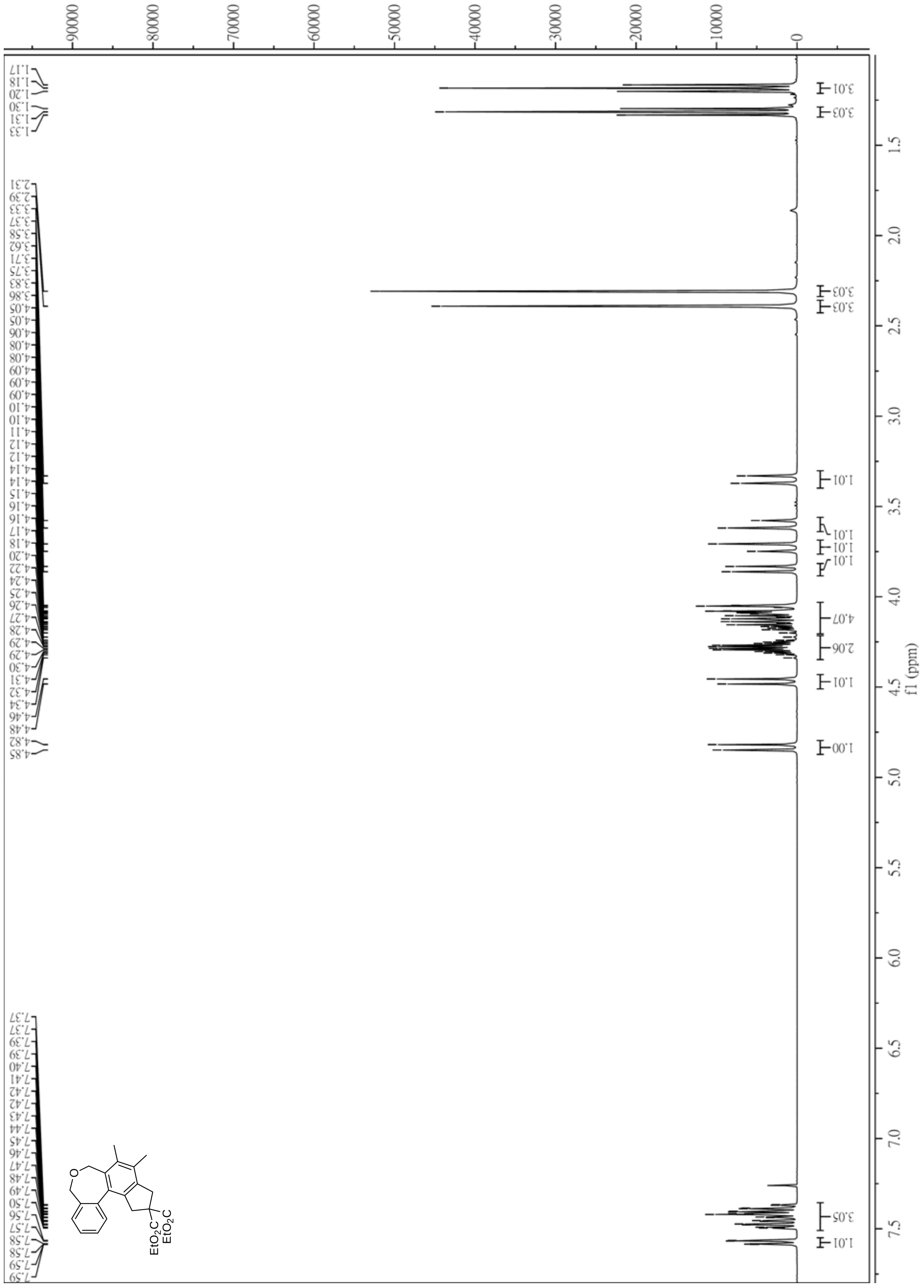

11,11-Di(carbethoxy)-8,9-dimethyl-7,10,11,12-tetrahydro-5H-benzo[e]indeno[5,4c]oxepine (6)

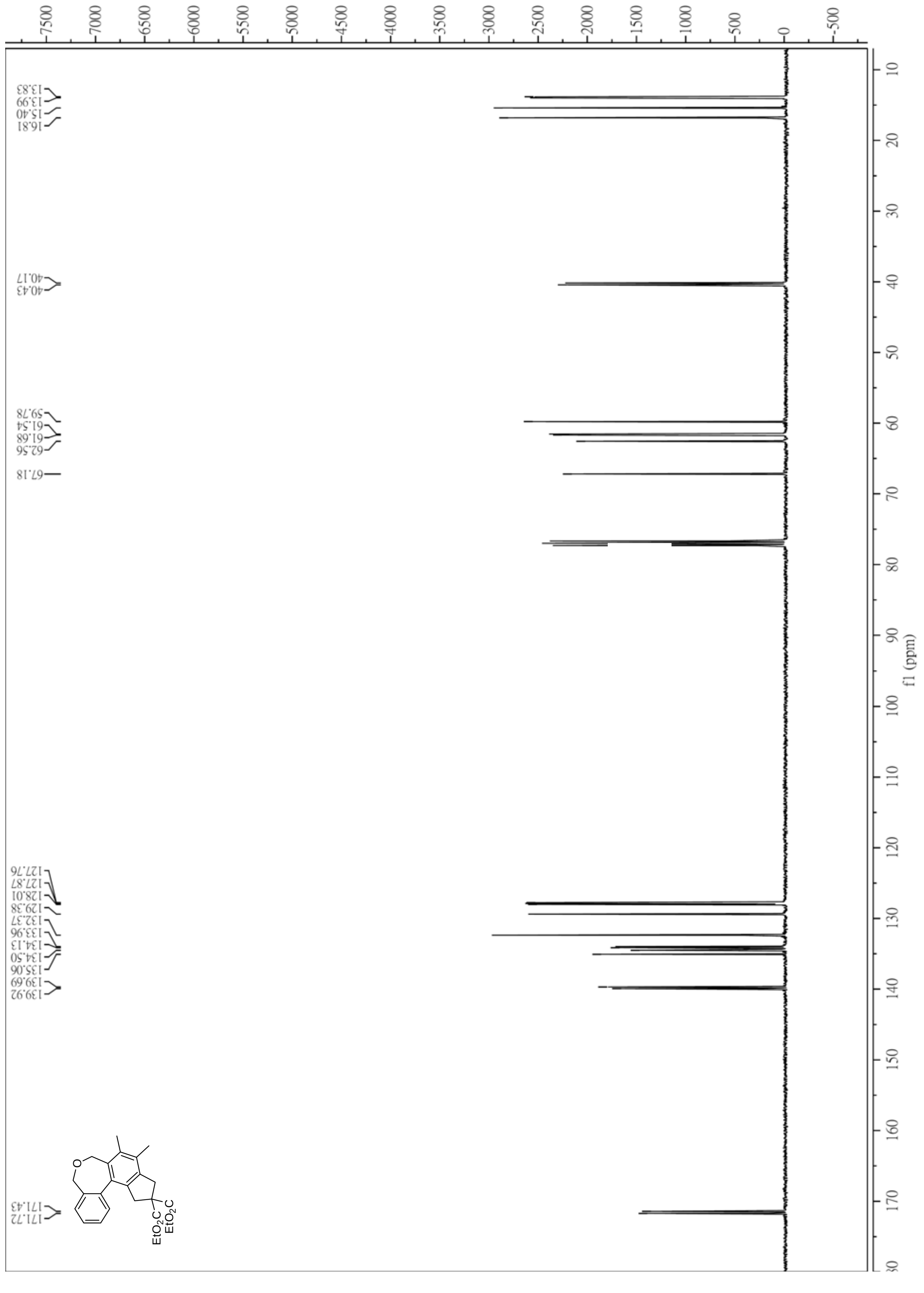

Supplement: Supplementary Data Sheet 1 — Energy diagrams based on DFT calculations with chemical structures for intermediates and transition states, 1H and 13C NMR spectra of triynes and fused tropone and benzene products. [file Data_Sheet_1.PDF]
